# Early over expression of messenger RNA for multiple genes, including insulin, in the Pancreatic Lymph Nodes of NOD mice is associated with Islet Autoimmunity

**DOI:** 10.1186/1755-8794-2-63

**Published:** 2009-10-02

**Authors:** Béatrice Regnault, José Osorio y Fortea, Dongmei Miao, George Eisenbarth, Evie Melanitou

**Affiliations:** 1 Institut Pasteur, 1 Genopole, Department of Genetics & Genomics 25-28 rue du Dr Roux, 75015 Paris, France; 2 Immunophysiology and Intracellular Parasitism Unit, Department of Parasitology and Mycology, 25-28 rue du Dr Roux, 75015 Paris, France; 3 Barbara Davis Center for Childhood Diabetes, University of Colorado, Aurora, CO 80010, USA

## Abstract

**Background:**

Autoimmune diabetes (T1D) onset is preceded by a long inflammatory process directed against the insulin-secreting β cells of the pancreas. Deciphering the early autoimmune mechanisms represents a challenge due to the absence of clinical signs at early disease stages. The aim of this study was to identify genes implicated in the early steps of the autoimmune process, prior to inflammation, in T1D. We have previously established that insulin autoantibodies (E-IAA) predict early diabetes onset delineating an early phenotypic check point (window 1) in disease pathogenesis. We used this sub-phenotype and applied differential gene expression analysis in the pancreatic lymph nodes (PLN) of 5 weeks old Non Obese Diabetic (NOD) mice differing solely upon the presence or absence of E-IAA. Analysis of gene expression profiles has the potential to provide a global understanding of the disease and to generate novel hypothesis concerning the initiation of the autoimmune process.

**Methods:**

Animals have been screened weekly for the presence of E-IAA between 3 and 5 weeks of age. E-IAA positive or negative NOD mice at least twice were selected and RNAs isolated from the PLN were used for microarray analysis. Comparison of transcriptional profiles between positive and negative animals and functional annotations of the resulting differentially expressed genes, using software together with manual literature data mining, have been performed.

**Results:**

The expression of 165 genes was modulated between E-IAA positive and negative PLN. In particular, genes coding for insulin and for proteins known to be implicated in tissue remodelling and Th1 immunity have been found to be highly differentially expressed. Forty one genes showed over 5 fold differences between the two sets of samples and 30 code for extracellular proteins. This class of proteins represents potential diagnostic markers and drug targets for T1D.

**Conclusion:**

Our data strongly suggest that the immune related mechanisms taking place at this early age in the PLN, correlate with homeostatic changes influencing tissue integrity of the adjacent pancreatic tissue. Functional analysis of the identified genes suggested that similar mechanisms might be operating during pre-inflammatory processes deployed in tissues i) hosting parasitic microorganisms and ii) experiencing unrestricted invasion by tumour cells.

## Background

Type 1 diabetes (T1D) is an autoimmune disease characterized by the absence of insulin due to the specific destruction of the insulin-producing β cells of the pancreatic islets. This is a progressive process taking over 20 weeks in the NOD mouse and several years in human patients to be completed [1]. The NOD animal model has been a valuable source of information for several aspects of disease pathogenesis [2]. Genetic studies have contributed to portray the complexity of the disease and have established that multiple loci are carrying genes implicated in T1D in human [3] and animal models [4]. In human, more than 6 genes contribute to the disease [5,6] while over 20 loci have been described in the NOD mouse [7] but only few possible candidate genes have been unequivocally defined [8] other than the H2^g7 ^*Idd1 *locus [7,9].

Despite intensive research, the initial causal events remain elusive since the selection of individual mice at early stages, prior to the overt clinical signs, represents a challenge. Indeed, even though the NOD mice are inbred not all animals develop the disease, with an incidence of 40-90% in females, depending on the colony [2]. Additional hindrances for selecting individual animals that will subsequently develop the disease with certainty are the low penetrance of the implicated genes and the influence of environmental factors. For these reasons, the exact mechanisms taking place prior to the onset of the pancreatic islet-damaging sustained inflammatory processes remain largely unknown.

The aim of our investigation was to evaluate the possibility of the existence of distinct gene expression profiles in order to eventually render possible the study of the early molecular changes, taking place before the onset of inflammation in autoimmune prone mice. One hypothesis is that during the post-weaning period in genetically autoimmune prone individuals (mouse or human), homeostatic changes prompt the immune system not to conform to physiological responses, but to instead trigger pathways that lead to the final autoimmune condition in later life. Exogenous factors including the more or less sustained presence of micro-organisms in otherwise microbe-free tissues may take place during early life stages [10]. This may influence the immune regulatory processes put in place to prevent, attenuate and/or repair the acute or sustained inflammation. It has been reported that early immune stimulation prevents autoimmune diseases, while infections later in life might exacerbate their advent in genetically prone individuals [11]. A single injection of BCG (the *Mycobacterium bovis *vaccine strain) is highly protective against T1D in the NOD mice [12] and BB rats [13] when administered early but it has no beneficial effect given after disease onset; similar to what has been observed in human [14]. These observations lead to the indication that the time "window" of opportunity to prevent disease in human may share similarities with the NOD mice and thus BCG or other immune-system signalling, safe biological preparation, should be given as therapy before the onset of inflammation of the pancreatic islets (insulitis). This early "window" coincides in the NOD mouse model with the early appearance of insulitis, occurring between 4 and 7 weeks [2] and led to the hypothesis that early initiating mechanisms should be evaluated at this age i.e. over the weaning period. We have previously established that IAA are present early between 3 and 5 weeks of age and represent a diabetes sub-phenotype since their presence in NOD mouse sera, at this early period, correlated with the final disease phenotype [15]. Thus Early-IAA (E-IAA) have been shown to mark the first measurable phenotypic checkpoint in T1D pathogenesis. Animals positive for E-IAA at 3-5 weeks after weaning, develop autoimmune diabetes earlier (between 16 and 20 weeks) than their E-IAA negative littermates [15]. We have used this E-IAA sub-phenotype to select individual mice as diabetes prone and search for changes in gene expression patterns in the PLN, correlating with the presence of E-IAA in the NOD mouse sera.

Overt T1D is considered to require the dominant presence of islet-destructive pro-inflammatory T lymphocytes [16]. The onset of T cell activation is known to deploy within organized secondary lymphoid tissues, draining the tissue that delivers the adequate signals for activation of T and B lymphocytes.

In the NOD mice, any early islet-disruptive remodelling process taking place, whether it implies β cell apoptosis or repair mechanisms [17], might result in delivery of immune cell-signalling to the PLN. The endpoint of such signalling might include deletion of autoreactive regulatory T cells, and/or expansion of autoreactive pro-inflammatory T lymphocytes as well as T cell-dependent autoreactive B lymphocytes. The later further differentiate as insulin auto-antibody secreting plasma cells [18]. Antigen specific T cells interact with dendritic cells (DC) within intact explanted lymph nodes (LN), and it has been observed that immunological synapse formation and prolonged interactions between these 2 cell types is followed by activation, dissociation and rapid migration of T cells away from the antigenic stimulus area [19]. Even more strikingly, excision of PLNs at 3 weeks of age protects NOD mice against IAA, insulitis and diabetes development, almost completely, but has no effect when performed at 10 weeks of age [20]. Therefore the time frame of 3 to 5 weeks seems to correspond to the initiation of the autoimmune process in the PLN and the related transcriptional profiling can be potentially sort out in this tissue.

We anticipated that at 5 weeks of age, differential gene expression correlated with the presence or absence of E-IAA, in the PLN of NOD mice, could be detected. This timing allowed for prior tests of E-IAA at 3 and 4 weeks. We used microarray analysis to determine gene expression patterns in the PLN. This approach has the potential i) to provide a global profiling of the modulated genes at this first measurable phenotypic checkpoint in T1D pathogenesis, ii) to allow novel hypothesis to be formulated concerning the initiation of the islet-destructive autoimmune process and eventually to further challenge these hypothesis. We identified ectopic high expression of insulin genes in the E-IAA positive PLN. This data confirm the possible cross talk between the pancreas and its adjacent lymph nodes *via *at least one pancreas specific autoantigen.

Functional annotations of up-regulated transcripts in the PLN of E-IAA positive NOD mice revealed a gene network otherwise found also to operate during inflammatory and tumour processes. Furthermore, we have identified several transcripts coding for extracellular proteins that have to be evaluated as potential disease pathogenesis associated markers and possibly therapeutic targets. This data represent a "freeze frame" of a first identifiable disease check point, associated with the presence of IAA and corresponding to the early steps of the autoimmune process.

## Methods

### Animals

Mice were purchased from Taconic farms (NOD/*tac*). Pregnant females were tested one week before delivery for the presence of IAA. To discard any possibility of trans-placental antibodies affecting our experimental design, based upon the spontaneous appearance of IAA, we have used, when possible, individual mice from progenies of negative females and from litters that they contained both positive and negative individuals.

Animals were tested as previously described [15], at 3, 4 and 5 weeks of age for the presence of IAA. PLN of IAA positive and negative animals were used for RNA preparation and pancreata have been also isolated for histology. All animals were kept under SPF conditions and all studies were performed under the recommended Laboratory Animal Care committee of the University of Colorado.

### IAA assay

IAA were measured in a standard radioimmunoassay as previously described [15,21] incorporating competition with unlabeled insulin and precipitation with Protein A/G sepharose in a 96 well filtration plate.

### Histology immunoassay

Tissues were formalin (10%) fixed and embedded in paraffin. Sections of paraffin-embedded tissues were stained with Hematoxylin & Eosin and with polyclonal guinea pig anti-insulin antibodies (Millipore) followed by incubation with a peroxidase-labeled anti-guinea pig IgG antibody (Kierkegaard & Perry Laboratories Inc.). The presence of insulitis has been evaluated by visualizing more than 10 islets in 3 different sections from the same individual. Similarly the presence of insulin expressed in the PLNs has been evaluated in more than 3 sections of each individual tissue.

### Tissues and RNA preparation

PLN have been dissected from six E-IAA negative and three E-IAA positive animals at 5 weeks of age. Tissues were immediately placed in RNA later containing RNases inhibitors (Qiagen) and processed for total RNA preparation on the same day with Qiagen mini-RNA commercial kits (RNeasy) with on-column DNase treatment following the manufacturer's protocol. Quantity, purity and integrity were confirmed initially by spectrophotometry (A_260_/A_280 _ratio) and capillary electrophoresis (2100 Bio analyzer; Agilent Technologies, Palo Alto, CA). 4.5 μg of total RNA were used for the target preparations and no additional step of cDNA amplification has been used in our studies.

### Microarray Analysis

Target preparations and chip hybridizations have been performed as recommended by the manufacturer MG_U74A_version 2 GeneChips (Affymetrix, Santa Clara, Ca). Each chip contained 12 486 probe sets (Supplemental Materials and Methods). All gene expression data discussed in this report have been deposited in NCBI's Gene Expression Omnibus [22] and are accessible through GEO Series accession number GSE15582 .

### Chromosomal location enrichment by Map genes to chromosomes

Genes used for hierarchical clustering have been also used to create the Genome view of our transcriptome data by using the dChip software (map of genes tool), as described in the dChip site .

*P*-values have been calculated for all stretches containing ≤20 selected genes to assess the statistical significance of gene proximity and the significant *P*-values have been reported in the analysis view. The "tightness" i.e. the rank distance of the genes on the two ends of the stretch was calculated by/n genes against that of n genes randomly put on the chromosome as described .

### Functional annotations

Gene Ontology analysis was carried out using DAVID NIAID/NIH online functional annotation tool and ontology terms were retained according to the significance of *P*-values for any particular GO term. This indicated that there were more genes associated with this GO term than would be expected by random chance [23]. The results were independently confirmed by applying the GO Browser tool in NetAffx to the same set of differentially expressed genes. The associations between altered genes or pathways were further evaluated using the Ingenuity Pathways Analysis software (Ingenuity^R ^Systems).

### Reverse transcription-coupled real-time PCR

Total RNAs from PLN of E-IAA positive and negative mice were used for Real Time PCR following the ABI software and method. To confirm gene expression levels identified in the arrays by RT-PCR, RNAs were aliquoted from the same samples used for the array hybridizations. Sequences corresponding to the investigated genes have been retrieved from the public database and aligned with Affymetrix target sequences using BLAST software . Primers and TaqMan probes [see Additional File 1] have been designed using the proprietary TaqMan software. We verified that the amplified fragments corresponded to the exact Affymetrix sequences. mRNA from an insulinoma cell line has been used as positive control and 18S rRNA for data normalization.

## Results

### E-IAA as a marker of autoimmune-related modifications in the PLN of NOD mice

We have established the following criteria for the selection of individual mice: autoimmune positive animals have been tested at 3, 4 and 5 weeks for the presence of IAA in the serum and selected when positive at least twice [24]. Negative phenotype has been considered when animals were negative for IAA at the same ages. A total of 9 animals have been selected, 6 negative for E-IAA and 3 E-IAA positive (Table 1). Even though the presence of E-IAA have been demonstrated to be an early marker of autoimmune diabetes, animals negative for IAA throughout their life may develop T1D, yet this remains a rare event (1 animal/15) [25]. In human, some cases of IAA long term positive individuals who do not develop T1D have been observed [26]. As seen on Table 1, regardless of the sub phenotypic attribution to the individual mice used in this study, one E-IAA negative sample (A36.4) was grouped, by clustering analysis, together with the E-IAA positive group, according to gene expression patterns (see below). This observation underlines the complexity of the autoimmune condition and indicates that the balance between pathogenic and physiological immune responses controls the outcome of disease. One possible explanation for the absence of E-IAA in the A36.4 PLN sample is that the selected sub-phenotype marks the disease stage without being *per se *the cause for the expression patterns identified. Therefore the initiation of the autoimmune process might be dependent upon the identified gene signatures and independent of the presence of IAA in the serum. In this case the E-IAA would be the consequence of an ongoing mechanism but not the cause.

**Table 1:** Subphenotypes of PLN used for transcriptome

**Sample Name**	**E-IAA Phenotype**	**Gene expression patterns**
A8.1	IAA neg	NEG
A12.1	IAA neg	NEG
A12.2	IAA neg	NEG
A36.1	IAA neg	NEG
A37.1	IAA neg	NEG
**A36.4**	**IAA neg**	**POS**
A9.6	IAA pos	POS
A15.4	IAA pos	POS
A15.6	IAA pos	POS

Correlation of E-IAA subphenotypes with gene expression patterns as shown by unsupervised hierarchical clustering on Fig. 2.

### E-IAA related gene expression patterns in PLN

Our interest in this study was to identify gene expression variations solely due to the presence or absence of IAA, in a set of identical animals of the same age and same genetic background, which however are not synchronized for the evolution of the disease. Albeit the NOD strain is inbred, homeostatic differences inherent to each animal might influence the physiopathological status and in consequence the disease stage. Therefore, RNAs have been prepared from PLN, isolated from individual mice and used without prior amplification. This allows identifying and controlling for inherent biological variability of gene expression, between individual animals that might not be related to the E-IAA sub-phenotype and allowed us avoiding the possibility for any preferential transcript amplification.

**Figure 1:**
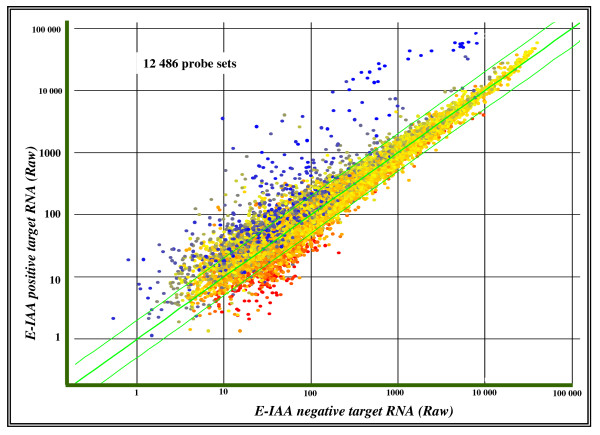
**Correlation of gene expression between E-IAA positive and negative PLN**. Scatter plot representation of all MU74Av2 array elements (GS6.0 software), after hybridization with the RNA probes. A subset of elements that are distinct between the two arrays and which deviate the most in signal intensity is depicted by the colour codes: blue for the highly expressed in the E-IAA positive PLNs, red for the highly expressed in the E-IAA negative samples and yellow for the probe sets that do not show statistical significant changes between the two sets of samples in both X and Y "fluorescence intensities".

The quality of whole gene expression data generated from each of the 9 individual animals has been checked using box plots metrics representation [see Additional File 2]. The mean fluorescence intensity values and the range of signal amplitudes were similar across all samples and consistent with a good hybridization quality of the arrays. A scatter plot of the raw data for all transcripts of the arrays from either E-IAA negative or positive samples clearly showed several probe sets to be differentially expressed between the 2 conditions (Figure 1). Expression levels for the up-regulated genes were higher in E-IAA positive PLN, while up-regulated variations in the E-IAA negative samples have been observed at a lower level (Figure 1).

**Figure 2:**
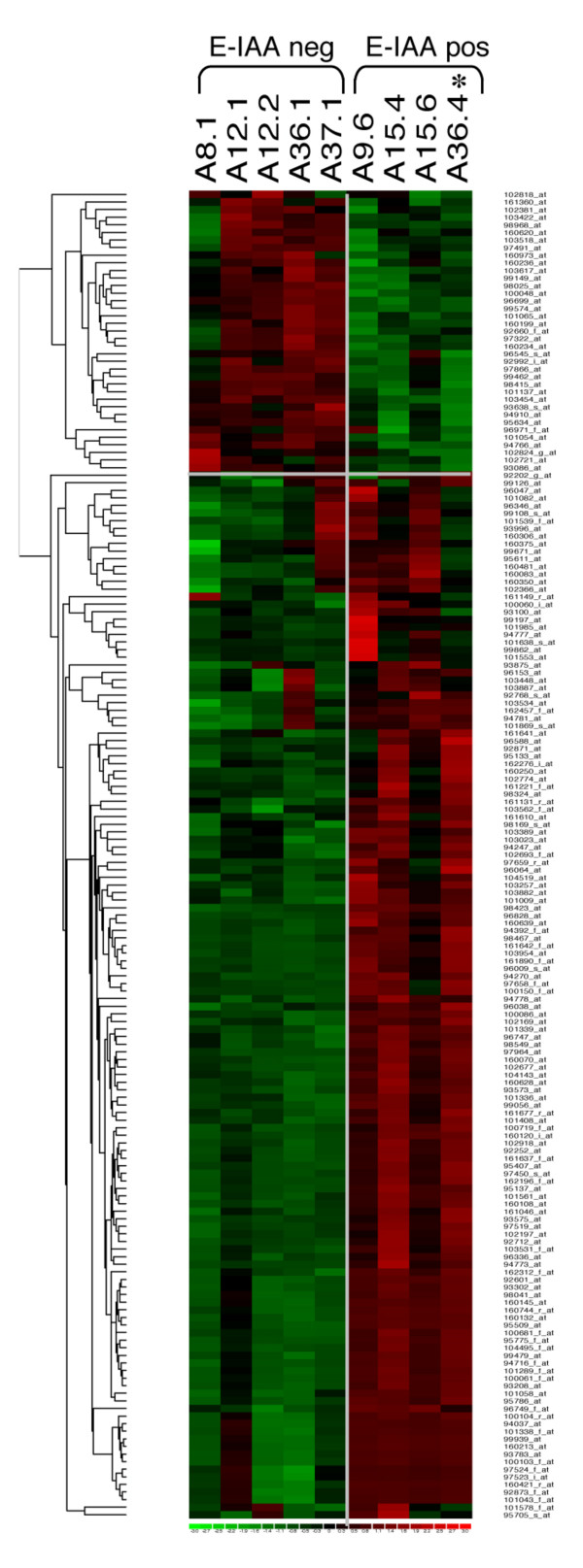
**Hierarchical clustering representation of differentially expressed probe sets between E-IAA positive and negative PLN samples**. Log_2 _transformed data from 177 probe sets are represented in a matrix format wherein each row displays expression results for a single gene across the arrays and each column shows the relative expression levels for all the genes in each sample. Red represents relative expression greater than the median expression level across all samples, and green represents an expression level lower than the median expression level. The colour intensity represents the magnitude of the deviation from the median. The dendrogram at the left lists the genes and provides a measure of the relatedness of their expression profile in each sample. *Sample A36.4 corresponds to E-IAA negative phenotype despite its clustering according gene expression with the E-IAA positive samples (see also Table 1).

Statistically significant differences in gene expression levels between the E-IAA positive and E-IAA negative lymph nodes were determined (adjusted *P*-values < 0.05 and log_2 _fold changes >0.42). A total of 177 probe sets were deemed statistically significant by these criteria and corresponded to 165 unique genes, whilst 12 transcripts were identified repeatedly because they were represented on the chip by more than one probe set [see Additional File 3]. Unsupervised hierarchical clustering of the modulated probe sets confirmed clear sample segregation in the two phenotypic groups (Figure 2). Gene expression patterns correlated well with phenotypic evaluation for all individual mice except one: sample A36.4 is classified with the positive group (Figure 2, noted by *) whilst its phenotypic evaluation, as described above (Table 1), has identified it as a negative sample. This discrepancy, as discussed above, correlates with previous findings in human and the NOD mouse, whereas autoantibody negative individuals have still a probability to develop the disease [25,27].

Interestingly, 77% (119 genes and 8 ESTs) of the differentially expressed transcripts were up regulated in the E-IAA samples while only 22% (34 genes and 3 ESTs) were down regulated. The majority of the up-regulated transcripts (69%, ie. 82 transcripts and 5 ESTs) have expression patterns varying over 2 fold while only 5 out of the 34 down-regulated genes and one out of the 3 ESTs (16%) showed over 2 fold differences between the two sets of samples [see Additional File 3].

The biological relevance of these trends in gene expression changes will obviously need to be tested on an individual gene basis.

### Validation of microarray data by Real time PCR and immunohistochemistry

**Figure 3:**
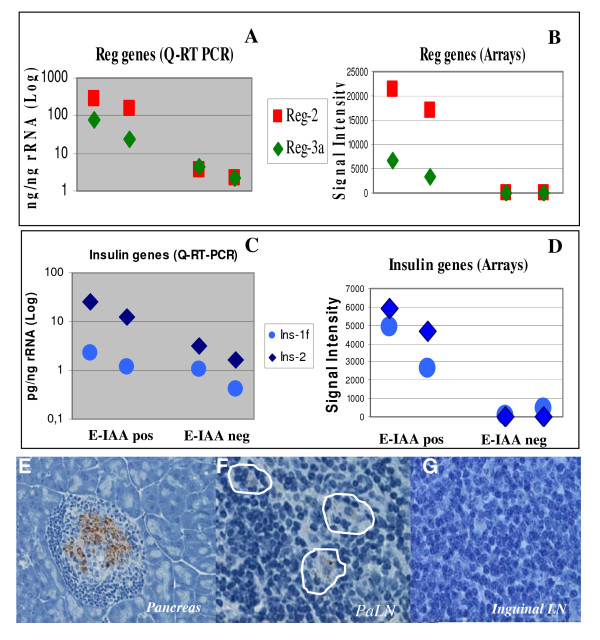
**Validation of expression patterns of selected genes differentially expressed in the PLN of NOD mice**. **A **and **B**: *Reg2 *and *Reg3a *genes (A: Real Time PCR and B: arrays row data). **C **and **D**: *Ins1 and Ins2 *genes (C: Real Time PCR; D: arrays row data). Samples are PLN RNA from E-IAApos mice (A9.6 & A15.4) and from E-IAA neg mice (A12.2 & A8.1). **E**, **F **&**G**. Staining of histological sections with anti-Insulin antibodies, from pancreas (**E**), PLN (**F**) and Inguinal lymph nodes (**G**) from NOD mouse at 5 weeks.

In order to validate the expression levels observed in the arrays, we selected 4 genes for quantitative real time PCR analysis (Q-RT-PCR): 2 regenerating islet-derived coding genes, *Reg*2 and *Reg3a *(Figure 3A &3B) and the 2 insulin genes, *Ins1 *and *Ins2 *(Figure 3C &3D) that have shown high gene expression differences in the arrays [see Additional File 3]. Similar trends of gene expression differences between the two sets of samples, in the arrays and in the Real time PCR were observed (Figure 3A-D).

Expression of insulin genes in the PLN was not expected since insulin is synthesized, stored and secreted by the β cells of the pancreatic islets in a highly regulated manner in human and mouse. However ectopic expression or so-called illegitimate transcription of self-antigens has been previously reported, mainly in the thymus [28] and spleen [29]. Cells expressing pancreatic autoantigens are also found in peripheral lymphoid tissues and the same cell can express more than one self antigen [15,30]. The functional significance of these observations has been questioned and evidence from studies in the mouse strongly suggested that self-antigen-expressing cells, in the thymus, may participate in negative selection and mediate tolerogenic signals, some of which may culminate in the apoptotic death of autoreactive lymphocytes [31].

Considering this data, we have stained NOD PLN with anti-insulin antibodies by immunohistochemistry (Figure 3F) and used pancreas as positive control (Figure 3E). Sparse distribution of insulin expressing cells was noted in the PLN (Figure 3F), while no staining has been obtained in inguinal lymph nodes used as negative controls (Figure 3G), thus confirming the ectopic insulin gene expression observed in our data. It is interesting to note that insulin genes have been also found expressed in the PLN of NOD mice at 4 weeks in a recent publication [32] although at lower levels.

Noteworthy that except insulin no other endocrine-specific transcripts (somatostatin, glucagon, somatostatin receptor or Islet amyloidal polypeptide) have been identified in our data set [see Additional File 4]. As expected and similarly to the endocrine specific transcripts, no other transcripts known to be specific either only to the exocrine pancreas or to the developing pancreas were expressed in the PLN at 5 weeks [see Additional File 4]. While expression of insulin genes in the PLN is not a novel finding, the high levels of expression found in the PLN of E-IAA positive NOD mice was not expected and is bewildering. One explanation might be that in the E-IAA positive PLN, APCs expressing insulin might act, locally in the PLN, as insulin-presenting cells. This would contribute to lack of tolerance to this molecule and consequently to the instigation of insulin autoreactive lymphocyte trafficking, leading to infiltration of the islets resulting to T1D. However a role of insulin in local tissue architecture, independent of its autoantigen function, cannot be excluded.

### Transcriptional signatures specific to E-IAA sub-phenotype

An important aspect of our experimental design concerns the early selection of NOD mice by the presence of E-IAA, used as a marker of early autoimmune activity, at an age whereas homeostatic changes in the pancreas might take place. It has been demonstrated that increased neonatal beta cell apoptosis is taking place with a peak at 2-3 weeks after birth, in T1D animal models including the BB rat and the NOD mouse [33]. The cross-talk between the pancreas and the adjacent LN during or immediately after this phase might be reflected by discrete gene expression changes in this later tissue.

We undertook a series of exploratory functional data analysis aiming to comprehend the roles of the identified gene expression variations in E-IAA-dependant islet autoimmunity. Forty-one out of the 119 up-regulated genes showed differential expressions over 5 fold [Additional File 3]. This indicates that the autoimmune process as reflected by the presence of IAA in the positive NOD mice is marked by local gene expression changes in the PLN. This might include changes in the immune process, occurring in this lymphoid tissue and/or changes in local tissue architecture and remodelling, not only of the lymph node but also of the pancreas, as mentioned above, assisted or mediated by the genes expressed in the PLN.

#### i. Cellular compartment localization

**Figure 4:**
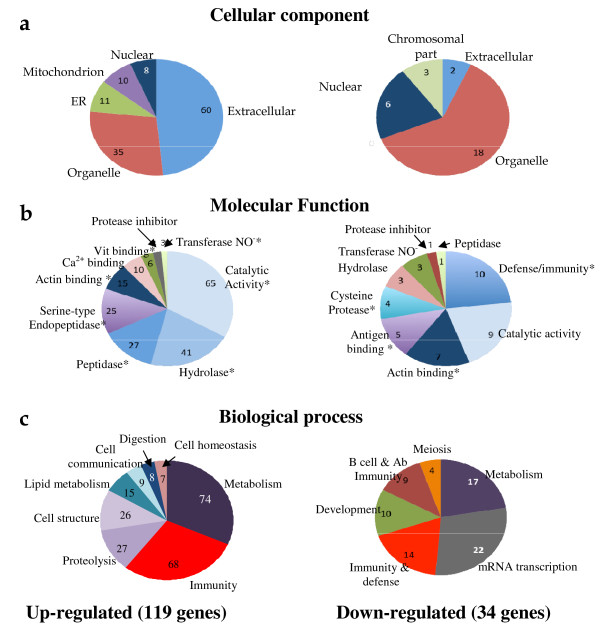
**Functional annotation for up- and down-regulated genes**. Functional categories distribution are according to GO and PANTHER annotations. **a. **Cellular component, **b. **Molecular function and **c. **Biological process. Significance for functional categories retained was 8.3E-19<*p *< 0.02 (* *P*-values ≤ 0.05). The gene list and corresponding *P*-values from each category are represented on Additional File 5.

An over representation of genes coding for extracellular proteins has been identified as 38% (62/165) modulated genes belonged to this category (*P*-value = 8.49E^-16^) (Table 2). This does not seem to be a random distribution since the first array of the MG-UG74Av2 series contains 2,057 transcripts coding for secreted proteins in a total of 12,488, representing 16% of the total number of genes on the arrays. Moreover this class of proteins is overrepresented in the E-IAA positive list of genes in the PLN (Figure 4a). The majority (30/41) of the over 5 fold expressed genes in the up-regulated samples, code for secreted proteins (GO *P*-value = 3.46E^-22^). This might reflect the cross talk taking place between the PLN and the pancreas, during the pre-inflammatory stages. Moreover secreted and membrane-associated proteins are an important class of proteins, as they have the potential to be detected in biological fluids and thus may be potential diagnostic markers or therapeutic targets.

**Table 2:** Annotations chart report for over-representation of functional categories.

	**UP & DOWN REGULATED**
**GO CATEGORY/Term**	**Gene Count (Ease score *P*-value)**

**Cellular compartment**	
Extracellular region	60 (9E-22) ↑, 2 ↓
Endoplasmic reticulum	11 (0.02) ↑
Nuclear	8↑ 6 (0.02) ↓
Organelle	35↑, 18 (0.03) ↓
Mitochondrion	10 (0.04) ↑

**Molecular function**	
Catalytic activity	65 (1.4E-5) ↑, 9 ↓
Hydrolase	41 (8.9E-8) ↑, 3 ↓
Peptidase activity	27 (2.9E-10) ↑ 1 ↓
Serine-type peptidase activity	25 (8.3E-19) ↑
Actin binding	15 (8.2E-4) ↑ 7 (0.02) ↓
Calcium binding **	10 (NS) ↑
Vitamin binding	6 (0.0008) ↑
Transferase NO-	3 (0.05) ↑, 3 ↓
Antigen binding**	5 (3.6E-4) ↓
Tissue kallikrein activity	8 (8.5E-12) ↑
Elastase activity	2 (2.5E-2) ↑

**Biological process**	
Metabolic process	74 (6.4E-4) ↑, 17 (0.02)
Immune-related process**	68^a ^↑ 14 (3.6E-6)
Proteolysis	27 (1.5E-11) ↑
Cell structure**	26 (0.02) ↑
Lipid, Fatty acid & steroid metabolism**	15 (1.31E-4) ↑
mRNA transcription regulation**	22(0.09) ↓
Cell communication**	9 (0.05) ↑
Digestion	8 (3.73E-8) ↑
Cellular homeostasis	7 (0.01) ↑

IMMUNE-RELATED PROCESS**	
B-cell & Antibody-mediated immunity**	9 (1.2E-4) ↓
Response to stress	18 (8.3E-5) ↑ 2.3E-2↓
MHC-I-mediated Immunity**	16 (0.09) ↑
Immunity & Defense**	16 (0.03) ↑ 4 (0.2) ↓
MHC-II-mediated Immunity**	15 (0.002) ↑
Response to external stimulus	14 (4.9E-5) ↑, 6 (0.5) ↓
Response to wounding	10 (5.09E-4) ↑
Inflammatory response	8 (0.001) ↑
Cytokine & chemokine mediated signaling	
pathway**	7 (0.06) ↑
Other Immune defense**	7 (0.05) ↑
Humoral Immune response	4 (2.2E-3) ↑
Antigen presentation	3 (1.1E-2) ↓
Lymphocyte-mediated immunity	3 (0.008) ↓
Adaptive Immunity^b^	3 (0.008) ↓

The most relevant GO [23] and PANTHER [38] terms associated with the list of 165 E-IAA modulated genes are shown. Gene counts are the number of genes in the list at the corresponding term and arrows indicate up and down-regulated gene expression. *P*-value is a modified Fisher exact *P*-value, Ease score. Graphical representations of functional categories are shown on Figure 4. The list of all genes is available in Additional File 5.

** PANTHER annotations

^a ^Significance for combined annotation from GO (EASE): 8.35E^-5^<p < 0.07 and from PANTHER tool: 0.002<p < 0.09.

^b ^Adaptive immune response based on somatic recombination of immune receptors built from immunoglobulin super family domains

Genes coding for secreted proteins include the *Spp1 *gene coding for osteopontin that has been found to play also a role in autoimmune demyelinating disease (MS) in human [34]. High levels of this pro-inflammatory cytokine have been reported in active relapsing-remitting MS patients [35]. Several other genes coding for secreted proteins with an immune/inflammatory function, include kallikreins genes (*Klk1b22, Klk1b9, Klk1b5, KlK1rb9, Klk1b1, Klk16*) that have been described as potential biomarkers for certain cancers [36]. Members of the kallikrein large family that belongs to serine-type proteases have been implicated in autoimmune diseases, in particular in a model of Sjögren's syndrome [37].

In contrast to the high representation of secreted proteins coding genes, nuclear proteins transcripts are more rare in our data set, representing 11% of the total number of the modulated genes (18/165) (Figure 4a). This could indicate that the high levels of mRNA found in the E-IAA positive PLN reflect a natural steady state process of gene transcription, initiated prior to 5 weeks and/or regulation of the mRNAs is taking place at the post transcriptional level.

#### ii. Functional significance of the PLN gene signatures

Aiming to correlate relevant functions of our transcriptome data with the E-IAA sub-phenotype and the end T1D phenotype, we assessed for functional significance of the identified genes. We applied two different approaches: **first **the identified differentially expressed genes between E-IAA positive and negative NOD mice have been subjected to Gene Ontology analysis (GO, EASE) [23] and evaluated for classification according to functional annotations for over-representation, in combination with assessment by PANTHER terms resource [38]. Thus in each category are included not only genes belonging to known functional categories by GO terms but also genes for which annotations are attributed by existing published experimental evidence together with consideration of evolutionary relationships (PANTHER).

In this analysis, terms with the highest levels of significance have been retained (for GO 8.35E^-5^<p < 0.07 and for PANTHER 1.2E^-4^<p < 0.02). Several genes have been annotated to Immune related process (Table 2) and several belong to the serine proteases group and include inflammation and immune functions (Table 2) and [see Additional File 5]. This group of proteins is characterized by the presence of a serine in the catalytic domain that is highly conserved between its members. Disorders of the dynamic balance of proteases and their inhibitors have been reported in the blood stream and in lymphocytes of diabetes patients and it has been proposed that increase of proteases concentrations represents an early marker of T1D [39]. Imbalance of the proteolytic system might also change the local homeostasis and interfere with the extracellular matrix of cells in the pancreas that become more permissive to lymphocyte invasion as it has been reported for tumour metastasis [40]. Further experimental evidence is required in order to comprehend the significance of the presence of serine proteases in the PLN of NOD mice.

Concerning the relevant distribution of functions between up- and down regulated gene lists, it is to be noted the presence of the serine-type proteases coding transcripts, found only in the up-regulated list (Figure 4b). In contrast, 5 antigen binding genes were all down-regulated in the E-IAA positive animals. Several immunoglobulin genes, including genes coding for the constant region of the kappa chains and the lambda variable region 1 are in this category as well as the Cd1d1 antigen (Table 3). Cd1d1 plays a critical role in the positive selection of T cells that produce Il-4 [41]. It has been reported that CD-1 reactive NKT cells are required for the development of systemic tolerance and defects of NKT cells affect autoimmunity [42,43]. NKT cells while they enhance the response to some bacterial, viral and parasitic infections and they play a similar role in some types of cancer, yet can suppress autoimmune disease, allograft rejection and graft-versus-host disease [44]. Therefore up-regulation of the Cd1d1 molecules in the E-IAA negative lymph nodes might prevent the development of autoimmunity *via *its binding to NKT-TCR. This can lead to expression of Il-4 by NKT cells [45]. Il-4 is known as a Th2 cytokine and therefore protective for T1D [2]. A similar protective mechanism has been described for experimental autoimmune encephalomyelitis (EAE), considered to be the prototype autoimmune disease mediated by type 1 helper T (Th1) cells, in the mouse [45].

**Table 3:** Putative functional categories of transcriptional signatures in E-IAA positive *versus *E-IAA negative PLN

**Putative Function**	Genes up-regulated*	Genes down-regulated*
Inflammation/Infection	***Reg2, Reg1, Try4, Pap, Tff2, Klk1, Try10/Try4, Reg3a, Klk1b22, Klk1b9, Klk1b5, KlK1rb9, Klk1b1, Cuzd1, Pla2g1b, Mt1, Nupr1, Mt2, Spp1, Klk1b16, Krt18, Ahsg, Krt8, Vtn, Klk1b5, Plg**, Klk1b26, Klk1b4, S100a9, Reg3γ, Hspa1a, Retn, S100a8, Slc23a3, Ghr*	
Immune function	***Spp1, Rnase 1, Sycn, Dmbt1, Ela1, Ela2A, Egfbp2, Ang, Fga, Crlf1, Crlf2, C1qb, Rnase4, Cfd, Ctrl, Gc**, Egf, Ngp*	**Ighg**, *Igl-V1*, AI324046/IgG1c, *Rap1a, Ms4a6b, Crlf3, Cd55, Ctla2b, Cd1d1, Myo5a, Cd74, Cr1*/*Igk-C*, LOC100046793 similar to *IgkV28*
Cell death and apoptosis	***Rnase1, Ins2, Alb, Mt1, Nupr1, Mt2, Spp1, Muc1, Krt18, Hba-a1, Ins1, Hpn, Krt8, Vtn, Plg, Alas2**, Gjb2, Egf, Lrpap1, Gstz1, Ptger3, Fzd3, S100a9, Dsp, Hspa1a, Aass, S100a8, Hbb-b2, Ghr, Zbtb16, Cyp2e1*	*Pcna, Cd55, Hnrpc, Cd1d1, Cd74, Oaz1, Eef1a1*
Cancer	***Prss3, Prss2, Dmbt1, Tff2, Mt1, Nupr1, Mt2, Spp1, Muc1, Ggh/LOC667301, Arhgdig, Tmem97, Itih4, Cyp3a11, Ang, Krt18, Gc, Ahsg, Sel1l, Hpn, Vtn, Actb, Plg**, Gjb2, Egf, Lrpap, Lpl, Asns, Fzd3, S100a9, Dsp, Lcat, Acta2, Tmem56, Rhou, Ndrg2 similar, Ces3, S100a8, Ghr, Zbtb16, Tmem30b, Cyp2e1*	***Hmgn1**, Top2b, Rap1a, Usp1, Pcna, Cd55, Cd1d1, Rps3, Cd74, Oaz1*
Cell-cell signalling	***Prss2, Ins2, Reg3a, Ins1, Alb, Spp1, Ang, Krt18, Gc, Fga, Krt8, Vtn, Plg**, Egf, Lrpap1, Lpl, Ptger3, S100a9, Acta2, S100a8, Ghr*	*Cd74, Cd1d1, Cd55*
Cell movement	***Prss3, Ins2, Tff2, Ins1, Cckar, Pla2g1b, Spp1, Muc1, Ang, Gc, Fga, Sel1l, Hpn, Vtn, Actb, Plg,, Ctrb1**, Gjb2, Egf, Lrpap1, Aldh1a7, S100a9, Lcat, S100a8, Zbtb16*	***Nde1**, Top2b, Cd55*
Diabetes	***Pnliprp1, Amy2-1, Ins2, Cel, Clps, Ins1, Amy1, Pnliprp2, Alb, Fkbp11, Rbp4, Mod1**, Cpn1*	
Regeneration & Remodelling	***Reg2, Reg1, Pap, Reg3a, Spp1, Itih4, Hbb-b2, Hba-a1, Itih2**, Reg3γ, Ptger3, Zbtb16*	*Evi2a, Oaz1*
Organizational injury	***Mt1, Nupr1, Mt2, Spp1, Krt18**, Hbb-b2, Krt8, Vtn, Plg, Egf, Lrpap1, Gstz1, Lpl, S100a9, Dsp, Acta2, Cyp2e1*	
Cell organization	***Ins2, Ins1, Pla2g1b, Mt1, Mt2, Spp1, Krt18, Krt8, Vtn**, Egf, Dsp, Rhou, Ghr*	***Nde1**, Rap1a, Myo5a*
Ubiquitination	***Fkbp11, Sel1l***	***Znrf2**, Hnrpc, Hnrpr, Usp1, Trim59, Ube2e1*
Transcription	***Nupr1, Ang, Pcbd1, Hbb-b2, Rnase4**, Foxa3, Zbtb16*	***Hmgn1**, Spic, Sp4*
Other	***Nucb2, Olfr93, Car3, Ero1lb, Pah, Gcat, Gv-1**, Copz2, Cdo1, Xist, Pck1*	***Slx/Xmr**, Slain1, Acsl4*
Unknown/EST	**AI593999**, **AE000663**, ***Cckar*, 1810009J0RIK, Arhgdig**, ***Gnmt*, *Pcbd1*, M26005**, **AV171666**, ***Gamt*, AE000663, *Gatm*, ***Thrsp*, AV250694, AW049643, AV067171	Gt(ROSA)26Sor, *Pcid2*, AA590345, AI842858, AI848107

Functional category annotations are considered significant with *P*-values < 0.05 as reported by Ingenuity Pathways Analysis software (Ingenuity^R ^Systems). Additional functions have been attributed to transcripts by literature data mining (see Additional File 6]. Transcripts are ordered according to levels of statistical significance for gene expression variation.* Genes with expression differences >2 fold are written in bold face font and normal font otherwise (1.5-2 fold).

In a **second **approach we used literature mining together with Ingenuity functional annotation tool (Ingenuity^® ^Systems). Combination of the resulting functional annotations for all the genes in the list [see Additional File 6] allowed assignment of more than one biological function to certain genes and gave an overall classification in twelve main functional categories (Table 3). Genes that did not clearly belong to any of the categories were classified together with ESTs, as '*unknown*' and comprised less than 12% of the modulated genes (Table 3). In our data set ESTs represented 7% of the identified genes, while in the chips, unknown expressed sequences represent approximately 50% of the 12,486 probe sets spotted (MG_U74A_v2 arrays).

Several transcripts belonged to more than one category (Table 3 and Additional File 6). Given the essential role attributed to the PLN in the activation of islet-specific immune response [46], it was assumed, at this pre- or peri-inflammatory stage (5 weeks of age) in the NOD mice, that several chemokines or adhesion molecules would be modulated in the autoantibody positive mice. However this has not been the case. While several inflammatory mediators were consistently expressed in all PLN from E-IAA positive and negative mice, few of these molecules were expressed in a significantly regulated manner (data not shown). This concerned genes coding for several chemokines (CCR7, CXCl12, CCR5, CXCl13, CCl21a and CXCl9) that although gave present calls (P) to all samples, did not meet statistical criteria for significant differences between the two groups (data not shown). One hypothesis might be that although all NOD mice possess the necessary requirements (i.e. presence of several chemokines in all samples) for the initiation of the inflammatory process against the pancreatic islets, the autoimmune-driven destructive events are probably dependent upon the levels of pancreatic tissue remodelling taking place. This in turn could be due to either antibody bearing B cells (IAA) or T lymphocyte specific trafficking against pancreatic signals.

### Genome view of the transcriptome signatures

**Figure 5:**
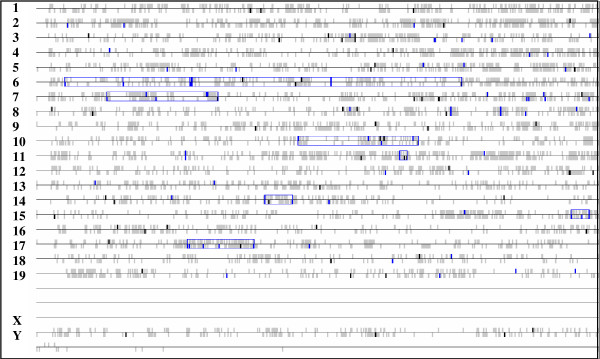
**Genome image view of genes identified in the PLN transcriptome**. Probe sets taken in consideration are the same as for hierarchical clustering (see Fig 2). In this "Genome view" genes in the gene list are coloured in black small vertical bars while the other genes are coloured in light-gray. The transcription starting site is used for gene position. The significant gene stretches are outlined in blue boxes and shorter stretches, when they exist, are contained within the longer ones that are indicated (methodology used is as described in ).

A genome view of our transcriptome data gave an unexpected image of chromosomal gene localization, pointing to mainly two chromosomes that showed stretches with the highest *P*-values containing >20 selected genes (Figure 5). This was the case for chromosome 6 (27 probe sets, p = 1.31E^-5^) and chromosome 7 (23 probe sets, p = 0.025). Two additional stretches on chromosome 10 and on chromosome 17 around the MHC locus were also detected but with a lower gene number.

Genes on the chromosome 6 are placed in 3 families including the lithostatin family, immunoglobulin light chain and several serine type proteases coding genes. Genes on chromosome 7 concerned mainly the kallikrein coding genes and the insulin-2 gene. Interestingly 4 T1D *Idd *loci have been described on these two chromosomes by genetic linkage analysis: two loci on mouse chromosome 6, the *Idd6 *[47] and *Idd19 *[48] and two *Idd *loci on mouse chromosome 7: the *Idd7 *[49] and the *Idd27 *[50].

## Discussion

Autoimmune diabetes in human is a heterogeneous disorder whereas several genes influence the apparent clinical phenotype together with environmental factors and homeostatic differences inherent to the organism. This leads to phenotypic variations of the disease observed not only in human but also in the NOD mouse, despite the genetic homogeneity of this inbred strain. Indeed autoantibody responses vary between individual mice by the titers and by the age of appearance. Similarly temporal variation in the severity of the islet-damaging inflammatory process is observed as well as variations in the onset of appearance of the end point disease.

In the present study we used the early presence of IAA in the NOD serum, as the earliest measurable sub-phenotype, aiming first to address for changes occurring in the pancreas-draining lymph nodes, when the autoimmune process has been just initiated. The PLN is a structured secondary lymphoid organ where pancreas derived cells and signals are expected to be delivered. The resulting output of the processing of these signals is either the maintenance or the loss of pancreas integrity. Our second aim was to attempt a stratification of disease check points by studying the first possibly identifiable disease stage, as selected by the presence of E-IAA.

This rational, potentially, allows identification of early genetic components that due to their specific expression in the PLN, at a precise time, may not be identified by genetic studies whereas the final T1D phenotype is usually taken in consideration.

### Biological significance of the identified transcriptional signatures and relevance to T1D phenotypes

Phenotypic stratification, as delineated by sub phenotypic attribution for the identification of gene expression variation in autoimmune diabetes, has not been used, to our knowledge, prior to our study. Two recent reports have also used PLN in a similar methodology approach, at early ages in the NOD mice, without however prior phenotypic evaluation [32,51]. One group studied 4 and 6 weeks old NOD/BDC 2.5 mice [51], the other group used NOD mice of various ages starting at 10 days and including 4 weeks, to compare with NOD.B10^H2b ^mice [32].

**Figure 6:**
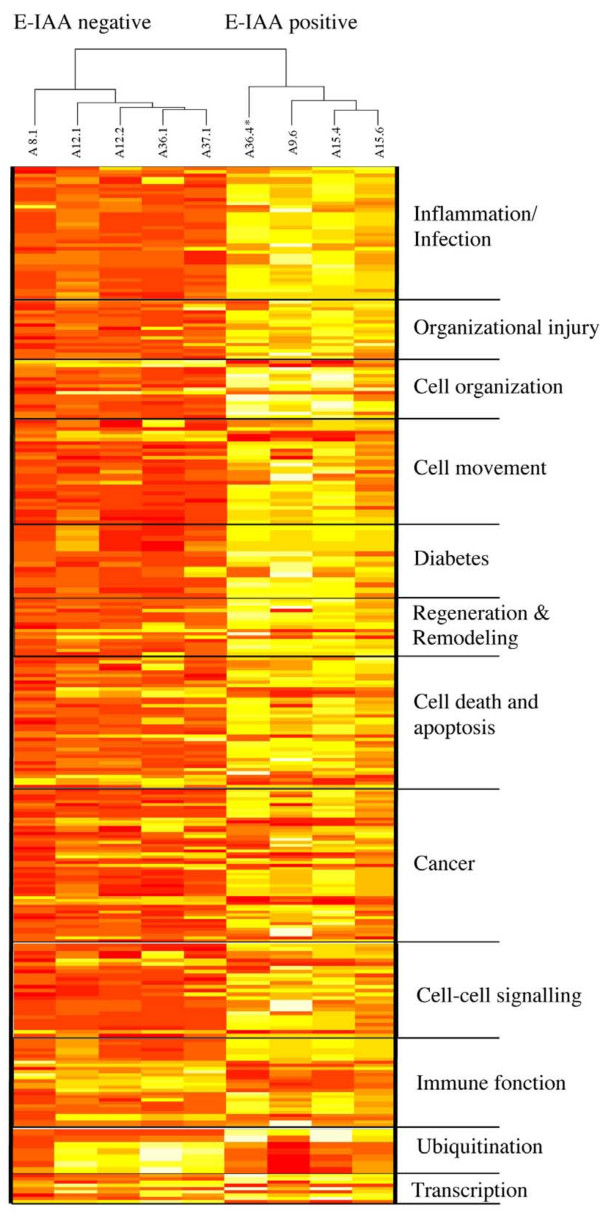
**Heat Map of signal intensities of gene expression patterns**. Genes found to be highly expressed in the E-IAA positive (yellow) negative (red) PLN according to functional annotations, as described on Table 3. The dendrogram, on the top of the figure, represents unsupervised hierarchical clustering of the samples, according to gene expression patterns. *Sample A36.4 corresponds to E-IAA negative phenotype despite its clustering according gene expression with the E-IAA positive samples (see also Table 1). Functional annotations have been ordered manually.

While our study has been carried out at 5 weeks of age it differs from these former investigations by the experimental design, based on the E-IAA sub phenotypic animal selection criteria. Accordingly, it can be assumed that gene expression changes solely correspond to the phenotype used for selection since the only perturbation, in our design, is the presence of E-IAA. While we cannot exclude that the identified gene profiles might correspond to the more rapid, though still infra-clinical progression to T1D, of the individual mice rather than to the E-IAA sub-phenotype, hierarchical unsupervised clustering (Figure 2) indicates however that samples are not randomly distributed. As the genetic background and the ages of the animals are indeed identical, this gives the opportunity to identify genes that might be activated in an autoimmune-dependent manner in the NOD mice, after or around the massive neonatal β cell apoptosis taking place [33]. It has been suggested that β cell death throughout the early ages in the NOD mice, may be an initial trigger for APC activation and the development of T1D [52]. Defects in silent clearance of apoptotic remnants before necrosis might prevent processes of the physiological maintenance of tissue integrity to operate [53]. In the rat intestine a discrete subpopulation of DCs has been found to transport apoptotic remnants to T cell areas of the mesenteric LN [54]. Interestingly several transcripts expected to contribute to physiological tissue remodelling were up-regulated in the E-IAA positive list (Table 3 & Figure 6).

### Tissue regeneration and remodelling

The expression of five genes belonging to the regenerating islet-derived gene family (*Pap*, *Reg1*, R*eg3a*, R*eg3g *and *Reg2*), implicated in tissue integrity, maintenance and regeneration were highly up-regulated in our data set [see Additional File 6]. They code for a set of related but distinct proteins [55]. The presence of these proteins in the E-IAA samples is intriguing, especially in the light of recent published reports demonstrating that adult human islets possess a remarkable degree of morphogenetic plasticity [56]. Similarly to *Reg *genes, *Itih*-*4*, a serine-type protease inhibitor with anti-apoptotic and matrix stabilizing functions, is also up-regulated and reported to play a role in liver development and regeneration [57]. These transcriptional signatures, if sorted out as such, might represent a new tool in the understanding of islet neogenesis and tissue preservation by inherent factors and could be valuable against β-cell destruction.

### Sustained inflammatory processes and tumour progression

Several of the modulated transcripts belong to complex networks of gene products acting in tissues during inflammatory sustained processes (ie. *Spp1*, *Klk*s, *Mt2*), or playing a role in tissue regeneration (ie. *Reg*s) and have been found also to be associated with neoplasia (Table 3), [see Additional File 6]. Certain kallikrein coding genes have been up-regulated in our data set and are considered possible biomarkers for cancer [58]. Similar expression patterns have been found for *Muc1 *gene that is highly expressed in gastric carcinoma and proposed to be involved in gastric carcinogenesis [59] as well as for the genes *Cckar*, *Ggh *[60,61] and *Ang *[62] that were reported to be implicated in the genesis of various tumours. *Ang*, in particular, is involved in the Notch signalling pathway related to neurovascular progression of pancreatic cancer and metastasis [63], as well as in multiple myeloma [64]. It has been reported that circulating levels of angiogenic cytokines can predict tumour progression and prognosis in neuroendocrine carcinomas of the gastro-entero-pancreatic system [62].

Finally, the gene coding for nucleobindin 2 (*Nucb*2) implicated in B cell lymphomas [65] was also up-regulated. It is a DNA binding protein, called also NEFA and its sequence contains a signal peptide, suggesting that it is a secreted or trans-membrane protein [66].

### Infection and inflammation

Several genes known to play a role in various inflammatory conditions were differentially modulated in our data set (Table 3). Such molecules include two elastase genes, *Ela1 *[67] and the neutrophil elastase *Ela2 *[68], known to play a role in peripheral tissues invaded by microbes [69], the *Spp1 *gene [70], and the secretory granule protein syncollin (*Sync*), expressed by neutrophils with a role in host defence to infections by invading bacteria, fungi, and protozoa [71]. *Sync *is also expressed in the exocrine pancreas and in the duodenum and colon [72]. Other gene products operating during the inflammatory process include the *Egfbp2*, part of the kallikrein gene family [73], the actin binding protein (*Actb*), the mentioned above *Muc1 *that is part of signalling proteins regulating cell adhesion/de-adhesion [74] and two genes coding for metallothionein, *Mt1 *and *Mt2*. Increased levels of *Muc1 *have been found in the small intestine of a cystic fibrosis mouse model [75]. Over expression of metallothioneins has been described in gastric cancer and in intestinal metaplasia and dysplasia [76]. Mice lacking metalothionein are more susceptible to *Helicobacter pylori *colonization and gastric inflammation, indicating a protective effect of these proteins against *H. pylori*-induced gastritis [77].

Interestingly, several lipases coding genes that have been associated with diabetes were up-regulated in the E-IAA positive PLN (Table 3). *Pla2g1b *gene has been described as a pro inflammatory molecule expressed in lung epithelium hosting *Pseudomonas aeruginosa *[78]. It is also implicated in glucose uptake by the liver, heart and muscle tissues under high glucose diet [79]. Inhibition of lipase activity was shown to reduce the incidence of type 2 diabetes mellitus and genetic variations in the *Clps *and *Pnlip *genes are associated with type 2 diabetes [80].

Two additional genes coding for pancreatic lipase related proteins *Pnliprp1 *and *Pnliprp2 *were highly up-regulated in the E-IAA PLN (Table 3) and [see Additional File 3]. Pnliprp2 protein plays a crucial role in the digestion of dietary fats in milk suckling mammals [81] while it seems to participate in T cell cytotoxicity. A T cell lipase shares 74% amino acid identity with the pancreatic Pnliprp2 [82].

### Could novel hypothesis be further explored in NOD mice at early post birth period?

Overall the data reported herein suggest that the events leading to islet-damaging autoimmune destruction might be rooted to early processes, some sharing common characteristics with several other inflammatory conditions found also in microbes-hosting tissues (*i.e. Pla2g1b, Mt1*) and in certain cancers (*i.e. Klk *genes, *Muc1*, *Reg *genes). Noticeably among the cytokine receptor-like factor 1 and 3 (*Crlf1, Crlf3*) that belong to the same family [83], *Crlf3 *has been found to be up-regulated in skin cancer [84]. While *Crlf1 *expression is up-regulated in the E-IAA positive samples, expression of the *Crlf3 *gene is down-regulated. *Crlf1 *mRNA has been reported to be up-regulated by TNF-α, IL-6, and IFN-γ [83]. Polymorphisms in the regulatory region of *C1qb *gene have been correlated with down-regulation of the murin c1q protein levels and linked to lupus nephritis [85].

The exact order of events in autoimmune initiation requires additional studies in order to be elucidated. Our hypothesis implies that defects in pancreas remodelling processes, taking place at the weaning period, might be a trigger for immune deregulation leading to autoimmune tissue destruction. This might include tissue regeneration or damage. The developmental stage of the organism as well as agents exogenous to the organism, such as invasive microbes, potentially can contribute to local homeostatic changes. It can be assumed that the identified genes have the potential to re-address the question of the link between environmental triggers, such as sustained microbial signalling processes, with the autoimmune process, at least in T1D.

This hypothesis requires certainly additional integrative studies and experimental evidence to be validated. It is noticeable that none of the common pro-inflammatory molecules such as tumour necrosis factor or interferons have been found in our group of genes. It has been recently reported 8 IFN-α inducible genes to be differentially up-regulated in CD4^+ ^T cells isolated from the PLN of 6 wks, when compared with 2 weeks old NOD/BDC 2.5 mice [51]. These authors concluded that IFN-α initiates T1D in the NOD mice and suggested that pDCs and TLRs play a role in T1D initiation. Though NOD mice differ from strains unable to initiate islet autoimmunity in terms of their response to IFN, our data indicate that such differences are not associated, within the NOD strain, with early expression of IAA, which in its turn is associated with greater risk of diabetes progression, at least at the age studied in this report.

## Conclusion

The autoimmune-related transcriptional early landscape of the PLN, in a first glance, seems difficult to be contained due to its complexity. The stepwise nature of the process resulting in T1D, is illustrated in our study by the difficulty to sort out a clear mechanism describing a possible scenario for disease initiation and implicating, preferably, a small number of molecules. Nevertheless, the gene signatures identified by this global analysis of the PLN in the autoantibody positive NOD mice at 5 weeks, together with the functional annotations described herein, represent a valuable tool in designing additional experiments for understanding the irreversible initiation of the autoimmune process in T1D. Experimental investigation of the functions of the identified genes together with further integrative analysis studies have the potential to sear light into the exact autoimmune-related processes that take place at this early age, in autoimmune prone, insulin autoantibody positive animals.

## Abbreviations

T1D: type 1 diabetes; NOD: Non Obese Diabetic; PLN: pancreatic lymph node; DC: Dendritic Cell; APC: Antigen Presenting Cells; BCG: Bacillus Calmette-Guérin; *Idd*: insulin-dependent diabetes locus.

## Competing interests

All authors are free of any commercial affiliation or consultancy or other arrangement that could be considered to pose a conflict of interest regarding this submitted article.

## Authors' contributions

EM and DM performed experiments, BR and EM analyzed data, JOyF participated in functional annotations analysis; EM conceived and coordinated the study, EM and GE designed the study, EM wrote the manuscript, GE supported the experimental part of the study in his laboratory.

All authors read and approved the final version of this manuscript.

## Pre-publication history

The pre-publication history for this paper can be accessed here:



## Supplementary Material

Additional file 1 **Sequences corresponding to probes used as primers for amplification of selected genes by Real Time Q-RT-PCR.** Click here for file

Additional file 2 **Quality evaluation of the arrays by box plots metrics**. RMA-normalized data distribution for the E-IAA positive and the E-IAA negative groups Click here for file

Additional file 3 **Genes differentially expressed in the PLN of 5 weeks old NOD mice according to E-IAA sub-phenotype and adjusted P-values.** Click here for file

Additional file 4 **Genes specifically expressed in the pancreas but not found to be expressed in the E-IAA PLN transcriptome.** Click here for file

Additional file 5 **Functional categories of genes and ESTs as annotated by GO and PANTHER tools.** Molecular functions and Biological process terms are as described in the text for Table 2. Click here for file

Additional file 6 **Assignment of functional categories as annotated by literature data mining, for genes and ESTs differentially expressed in the PLN of E-IAA NOD sub-phenotype, as described in the text (Table **3**).** References, corresponding to bibliographic search for functions for each gene, are given. Click here for file
