# Supplementary material for: Early over expression of messenger RNA for multiple genes, including insulin, in the Pancreatic Lymph Nodes of NOD mice is associated with Islet Autoimmunity
Source: BMC Med Genomics. 2009 Oct 2;2:63. doi: 10.1186/1755-8794-2-63 (PMC2763872; doi:10.1186/1755-8794-2-63)
Supplement: Additional file 1 — Sequences corresponding to probes used as primers for amplification of selected genes by Real Time Q-RT-PCR. [file 1755-8794-2-63-S1.PDF]

Sequences corresponding to probes used as primers for amplification of selected genes by Real Time Q-RT-PCR

Gene	Forward	TaqMan probe	Reverse
Ins-1f (97658-f)	5'-GAA GTG GAG GAC CCA CAA GTG-3'	AACAACTGGAGCTGGGAGGAA GCCC	5'-GCC AAG GTC TGA AGG TCC C-3'
Ins-1r (97659-r)	5'-TGG TGC ACT TCC TAC CCC TG-3'	TGGCCCTGCTTGCCCTCTGG	5'-TTG ACA AAA GCC TGG GTG G-3'
Ins-2 (100150-f):	5'-GGG AGC AGG TGA CCT TCA GA-3'	TTG GCA CTG GAG GTG GCC CAG	5'-TGA TCT ACA ATG CCA CGC TTC T-3'
Reg-1 (160213)	5'-CAA TGC CTA CAG CTC CTA TTG TTA CT-3'	CTTCACTGAAGACCGTTTAACT TGGGCTA	5'-CCC CTA CTT GCT CAC ATC TGC-3'
Reg-2 (95786)	5'-CCC CTT GGC TGA AAA AGA CC-3'	TCCTTCTGCCAAAATCAACTGC CCA	5'-CAT AGG CGT TGG CAC CCT-3'
Reg-3a (103954)	5'-GGT GAG GCT TCC TTT GTG TGT CC-3'	CCTTGGTGAACGGCAGAGTGG ACAA	5'-CAG CCC AAT CCA GAT GTC TTG-3'
RNA-18S/Control	5'-CGA TGG TAG TCG CCG TGC-3'	TGGTGACCACGGGTGACGGG	5'-TCC GGA ATC GAA CCC TGA-3'
