# Supplementary material for: Early over expression of messenger RNA for multiple genes, including insulin, in the Pancreatic Lymph Nodes of NOD mice is associated with Islet Autoimmunity
Source: BMC Med Genomics. 2009 Oct 2;2:63. doi: 10.1186/1755-8794-2-63 (PMC2763872; doi:10.1186/1755-8794-2-63)
Supplement: Additional file 2 — Quality evaluation of the arrays by box plots metrics. RMA-normalized data distribution for the E-IAA positive and the E-IAA negative groups [file 1755-8794-2-63-S2.PDF]

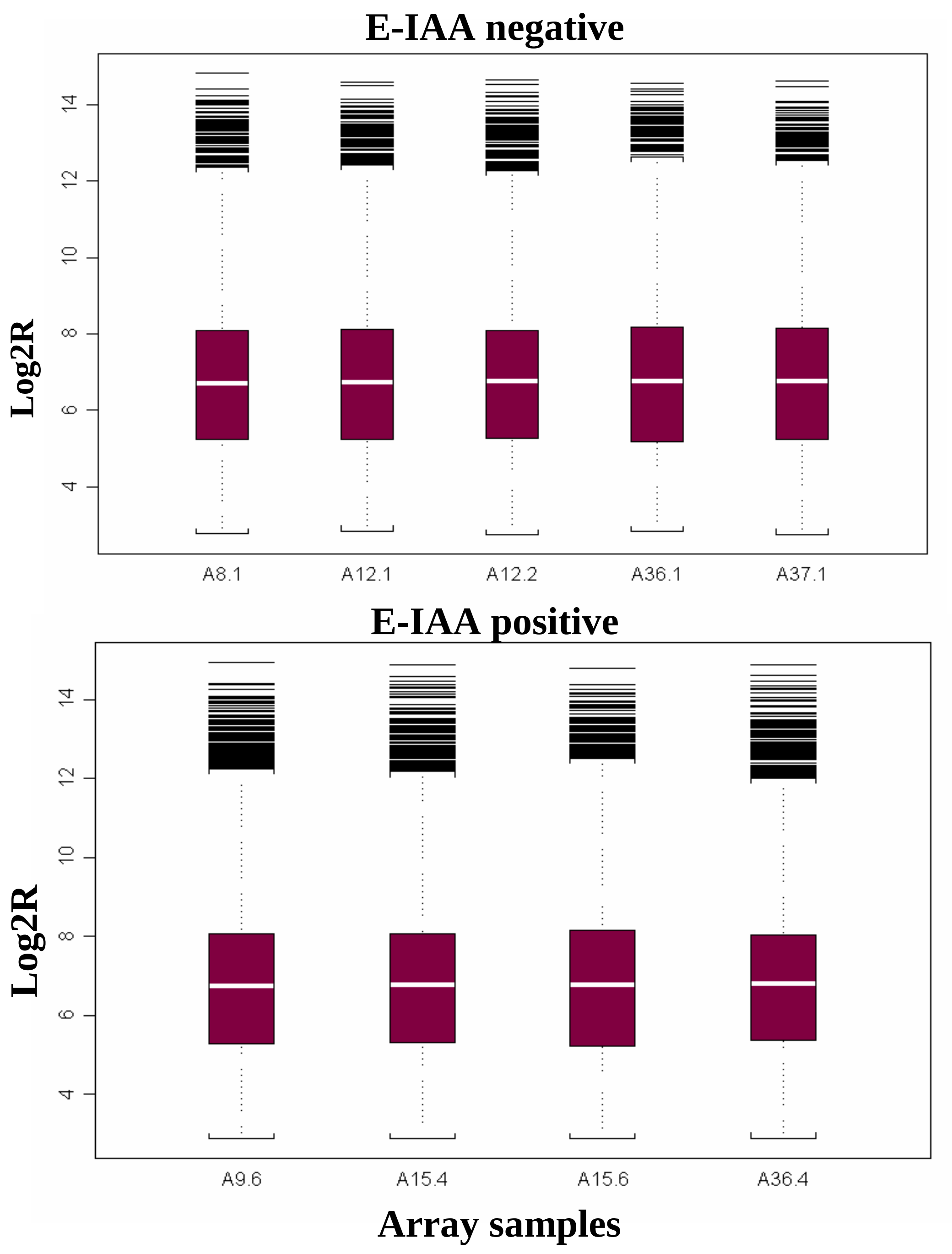

Quality evaluation of the arrays by box plots metrics.

RMA-normalized data distribution for the E-IAA positive and the E-IAA negative groups (Bolstad *et al*, 2003). The *boxes* depict the mean values of intensities, and the height of the *boxes* shows the range of signal amplitudes for 50% of data in each sample. The *vertical bars* illustrate the magnitude of variation within each group (neg and pos) for the remaining 50% of the data.
