# Supplementary material for: Early over expression of messenger RNA for multiple genes, including insulin, in the Pancreatic Lymph Nodes of NOD mice is associated with Islet Autoimmunity
Source: BMC Med Genomics. 2009 Oct 2;2:63. doi: 10.1186/1755-8794-2-63 (PMC2763872; doi:10.1186/1755-8794-2-63)
Supplement: Additional file 3 — Genes differentially expressed in the PLN of 5 weeks old NOD mice according to E-IAA sub-phenotype and adjusted P-values. [file 1755-8794-2-63-S3.PDF]

Genes differentially expressed in the PLN of 5 weeks old NOD mice according to E-IAA sub phenotype and adjusted *P*-values.

**A. Genes up-regulated between E-IAA positive and negative PaLN**

| Entrez ID | Affymetrix Probeset | Gene name | Description | Log2 change | Fold change | Adj *p-Value* |
|---|---|---|---|---|---|---|
| 19752 | 98041_at | Rnase1 | Ribonuclease, RNase A family, 1 (pancreatic) | 6,55 | 93,56 | 0 |
| 18946 | 92601_at | Pnliprp1 | pancreatic lipase related protein 1 | 6,14 | 70,76 | 0 |
| 100040233 | 93208_at | Prss3 | protease, serine, 3 | 6,09 | 68,26 | 0 |
| 19693 | 95786_at | Reg2 | regenerating islet-derived 2 | 5,87 | 58,44 | 0 |
| 68416 | 95509_at | Sycn | syncollin | 5,84 | 57,36 | 0 |
| 19692 | 162312_f_at, 160213_at | Reg1 | regenerating islet-derived 1 | 5,83 | 57,02 | 0 |
| 109959 | 97523_i_at; 97524_f_at | Amy2-1 | amylase 2-1, pancreatic | 5,82 | 56,49 | 0 |
| 22074 | 101043_f_at | Try4 | trypsin 4 | 5,73 | 52,90 | 0 |
| 22072 | 92873_f_at | Prss2 | protease, serine, 2 | 5,61 | 48,73 | 0 |
| 16334 | 100150_f_at | Ins2 | insulin II | 5,52 | 45,73 | 0 |
| 18489 | 161890_f_at, 96009_s_at | Pap | pancreatitis-associated protein | 5,51 | 45,63 | 0 |
| 12945 | 99479_at | Dmbt1 | deleted in malignant brain tumors 1 | 5,37 | 41,28 | 0 |
| 12613 | 99939_at | Cel | carboxyl ester lipase | 5,33 | 40,20 | 0 |
| 21785 | 93302_at | Tff2 | trefoil factor 2 (spasmolytic protein 1) | 5,32 | 39,97 | 0 |
| 16612 | 100061_f_at | Klk1 | kallikrein 1 | 5,24 | 37,92 | 0 |
| 109901 | 93783_at | Ela1 | elastase 1, pancreatic | 5,13 | 34,91 | 0 |
| 109791 | 160132_at | Clps | colipase, pancreatic | 5,09 | 33,99 | 0 |
| 109660 | 160744_r_at | Ctrl | chymotrypsin-like | 5,09 | 33,97 | 0 |
| 66473 | 160421_r_at | Ctrb1 | chymotrypsinogen B1 | 4,87 | 29,23 | 0 |
| 436522 | 101338_f_at | Try10 | trypsin 10 | 4,83 | 28,39 | 0 |
| 19694 | 103954_at; 161642_f_at | Reg3a | regenerating islet-derived 3 alpha | 4,81 | 28,09 | 0 |
| 13646 | 101289_f_at | Klk1b22 | kallikrein 1-related peptidase b22 | 4,43 | 21,55 | 0 |
| 13648 | 94716_f_at | Klk1b9 | kallikrein 1-related peptidase b9 | 4,30 | 19,71 | 0 |
| 16622 | 104495_f_at | Klk1b5 | kallikrein 1-related peptidase b5 | 4,12 | 17,34 | 0 |
| 16333 | 97658_f_at, 97659_r_at | Ins1 | insulin I | 4,03 | 16,28 | 0 |
| 11722 | 101058_at | Amy1 | amylase 1, salivary | 3,77 | 13,63 | 0 |
| 16623 | 95775_f_at | Klk1b1 | kallikrein 1-related peptidase b1 | 3,71 | 13,04 | 0 |
| 12425 | 92252_at | Cckar | cholecystokinin A receptor | 3,57 | 11,91 | 0 |
| 13706 | 94037_at | Ela2A | elastase 2A | 3,56 | 11,80 | 0 |
| 16433 | 162196_f_at | Cuzd1 | CUB and zona pellucida-like domains 1 | 3,55 | 11,73 | 0 |
| 18947 | 160070_at | Pnliprp2 | pancreatic lipase-related protein 2 | 3,48 | 11,15 | 0 |
| 13647 | 161637_f_at | Egfbp2 | epidermal growth factor binding protein type B | 3,36 | 10,26 | 0 |

| | | | | | | |
|---|---|---|---|---|---|---|
| 18478 | 95407_at | Pah | phenylalanine hydroxylase | 3,28 | 9,73 | 0 |
| 18778 | 160120_i_at | Pla2g1b | phospholipase A2, group IB, pancreas | 3,28 | 9,69 | 0 |
| 11657 | 94777_at | Alb | albumin | 2,91 | 7,52 | 0 |
| 17748 | 93573_at | Mt1 | metallothionein 1 | 2,77 | 6,81 | 0 |
| 56312 | 160108_at | Nupr1 | nuclear protein 1 | 2,70 | 6,49 | 0 |
| 17750 | 101561_at | Mt2 | metallothionein 2 | 2,61 | 6,11 | 0 |
| 20750 | 97519_at | Spp1 | secreted phosphoprotein 1 | 2,51 | 5,68 | 0 |
| 17829 | 102918_at | Muc1 | mucin 1, transmembrane | 2,39 | 5,23 | 0 |
| 14590 | 93575_at | Ggh | gamma-glutamyl hydrolase | 2,25 | 4,76 | 0 |
| 14570 | 102677_at | Arhgdig | Rho GDP dissociation inhibitor (GDI) gamma | 1,95 | 3,85 | 0 |
| 69071 | 95137_at | Tmem97 | transmembrane protein 97 | 1,92 | 3,78 | 1,5E-10 |
| 16427 | 98467_at | Itih4 | inter alpha-trypsin inhibitor, heavy chain 4 | 1,90 | 3,73 | 0 |
| 14711 | 96828_at | Gnmt | glycine N-methyltransferase | 1,86 | 3,62 | 0 |
| 16615 | 100719_f_at | Klk1b16 | kallikrein 1-related peptidase b16 | 1,78 | 3,44 | 0 |
| 13112 | 101638_s_at | Cyp3a11 | cytochrome P450, family 3, subfamily a, polypeptide 11 | 1,78 | 3,42 | 0,00637 |
| 11727 | 94392_f_at | Ang | angiogenin, ribonuclease, RNase A family, 5 | 1,77 | 3,42 | 3,1E-11 |
| 13180 | 99056_at | Pcbd1 | pterin 4 alpha carbinolamine dehydratase/dimerization cofactor of hepatocyte nuclear factor 1 alpha (TCF1) 1 | 1,75 | 3,37 | 6,8E-13 |
| 16668 | 94270_at | Krt18 | keratin 18 | 1,66 | 3,15 | 0 |
| 66120 | 97964_at | Fkbp11 | FK506 binding protein 11 | 1,64 | 3,11 | 1,2E-09 |
| 14473 | 99197_at | Gc | group specific component | 1,62 | 3,07 | 0,00096 |
| 15130 | 103534_at; 101869_s_at | Hbb-b2; Hbb-b1 | hemoglobin, beta adult minor chain | 1,59 | 3,02 | 1,4E-09 |
| 11625 | 99862_at | Ahsg | alpha-2-HS-glycoprotein | 1,59 | 3,00 | 0,00807 |
| 110257 | 94781_at; 162457_f_at | Hba-a1 / Hba-a2 | hemoglobin alpha, adult chain 1 | 1,49 | 2,82 | 9,9E-14 |
| 14161 | 101553_at | Fga | fibrinogen, alpha polypeptide | 1,41 | 2,66 | 0,00035 |
| 100041156 | 103562_f_at | Gv-1 | murine leukemia retrovirus | 1,40 | 2,64 | 0,00647 |
| 14431 | 101408_at | Gamt | guanidinoacetate methyltransferase | 1,33 | 2,52 | 2,4E-06 |
| 53322 | 102197_at | Nucb2 | nucleobindin 2 | 1,31 | 2,48 | 3,8E-05 |
| 12931 | 161046_at | Crlf1 | cytokine receptor-like factor 1 | 1,29 | 2,45 | 2,2E-05 |
| 435889 | 101339_at | Prss3 | protease, serine, 3 | 1,28 | 2,43 | 3,4E-05 |
| 110695 | 97450_s_at | Aldh7a1 | aldehyde dehydrogenase family 7, member A1 | 1,27 | 2,42 | 2,7E-05 |
| 26912 | 160628_at | Gcat | glycine C-acetyltransferase (2-amino-3-ketobutyrate-coenzyme A ligase) | 1,27 | 2,40 | 0,0004 |
| 258051 | 102169_at | Olfr93 | olfactory receptor 93 | 1,21 | 2,31 | 3,7E-05 |

| | | | | | | |
|---|---|---|---|---|---|---|
| 16425 | 104519_at | Itih2 | inter-alpha trypsin inhibitor, heavy chain 2 | 1,21 | 2,31 | 0,00111 |
| 20338 | 92871_at | Sel1l | sel-1 suppressor of lin-12-like (C. elegans) | 1,18 | 2,27 | 2,3E-06 |
| 15451 | 160639_at | Hpn | hepsin | 1,17 | 2,25 | 1,6E-06 |
| 17436 | 101082_at | Mod1 | malic enzyme, supernatant | 1,17 | 2,25 | 9E-07 |
| 12350 | 160375_at | Car3 | carbonic anhydrase 3 | 1,16 | 2,23 | 0,00377 |
| 12260 | 162276_i_at | C1qb | complement component 1, q subcomponent, beta polypeptide | 1,16 | 2,23 | 0,00035 |
| 67092 | 96336_at | Gatm | glycine amidinotransferase (L-arginine:glycine amidinotransferase) | 1,14 | 2,20 | 0,00416 |
| 67475 | 103531_f_at | Ero1lb | ERO1-like beta (S. cerevisiae) | 1,13 | 2,20 | 0,00018 |
| 19662 | 96047_at | Rbp4 | retinol binding protein 4, plasma | 1,13 | 2,18 | 0,0144 |
| 16691 | 101009_at | Krt8 | Keratin 8 | 1,11 | 2,15 | 0 |
| 22370 | 98549_at | Vtn | vitronectin | 1,09 | 2,13 | 0,00065 |
| 11461 | 101578_f_at, 95705_s_at | Actb | actin, beta, cytoplasmic | 1,09 | 2,13 | 7,2E-09 |
| 58809 | 96038_at | Rnase4 | ribonuclease, RNase A family 4 | 1,08 | 2,12 | 0,0057 |
| 16612 | 100060_i_at | Klk1 Klk1b5 | kallikrein 1 | 1,07 | 2,10 | 2,9E-07 |
| 18815 | 101985_at | Plg | plasminogen | 1,06 | 2,08 | 0,0113 |
| 11656 | 92768_s_at | Alas2 | aminolevulinic acid synthase 2, erythroid | 1,05 | 2,06 | 0,00139 |
| 11537 | 99671_at | Cfd | complement factor D (adipsin) | 1.00 | 2.00 | 0.0255 |
| 16618 | 102693_f_at | Klk1b26 | kallikrein 1-related petidase b26 | 0.99 | 1.98 | 0.00000338 |
| 56358 | 104143_at | Copz2 | coatomer protein complex, subunit zeta 2 | 0.97 | 1.96 | 0.00282 |
| 14619 | 98423_at | Gjb2 | gap junction membrane channel protein beta 2 | 0.97 | 1.96 | 0.00356 |
| 13645 | 102774_at | Egf | epidermal growth factor | 0.96 | 1.94 | 0.00493 |
| 16976 | 100086_at | Lrpap1 | low density lipoprotein receptor-related protein associated protein 1 | 0.96 | 1.94 | 0.0207 |
| 18534 | 160481_at | Pck1 | phosphoenolpyruvate carboxykinase 1, cytosolic | 0.95 | 1.93 | 0.00000148 |
| 18048 | 100060_i_at; 94773_at | Klk1b4 | kallikrein 1-related pepidase b4 | 0.93 | 1.90 | 0.00000105 |
| 93721 | 103882_at | Cpn1 | carboxypeptidase N, polypeptide 1 | 0.92 | 1.90 | 0.00181 |
| 21835 | 160306_at | Thrsp | thyroid hormone responsive SPOT14 homolog (Rattus) | 0.91 | 1.88 | 0.000000584 |
| 14874 | 160350_at | Gstz1 | glutathione transferase zeta 1 (maleylacetoacetate isomerase) | 0.91 | 1.87 | 0.0171 |
| 16956 | 95611_at, 160083_at | Lpl | lipoprotein lipase | 0.90 | 1.87 | 0.00144 |

| | | | | | | |
|---|---|---|---|---|---|---|
| 26358 | 94778_at | Aldh1a7 | aldehyde dehydrogenase family 1, subfamily A7 | 0.90 | 1.86 | 0.00198 |
| 19218 | 96588_at | Ptger3 | prostaglandin E receptor 3 (subtype EP3) | 0.89 | 1.86 | 0.0000157 |
| 12583 | 96346_at | Cdo1 | cysteine dioxygenase 1, cytosolic | 0.88 | 1.84 | 0.0000323 |
| 27053 | 161221_f_at, 95133_at | Asns | asparagine synthetase | 0.88 | 1.84 | 0.0268 |
| 14365 | 98169_s_at | Fzd3 | frizzled homolog 3 (Drosophila) | 0.88 | 1.84 | 0.0447 |
| 20202 | 103887_at | S100a9 | S100 calcium binding protein A9 (calgranulin B) | 0.86 | 1.81 | 0.000316 |
| 109620 | 94247_at | Dsp | desmoplakin | 0.85 | 1.80 | 0.0000157 |
| 16816 | 103023_at | Lcat | lecithin cholesterol acyltransferase | 0.84 | 1.78 | 0.00318 |
| 19695 | 96064_at | Reg3g | regenerating islet-derived 3 gamma | 0.83 | 1.77 | 0.0113 |
| 11475 | 93100_at | Acta2 | actin, alpha 2, smooth muscle, aorta | 0.82 | 1.77 | 0.00329 |
| 15377 | 98324_at | Foxa3 | forkhead box A3 | 0.82 | 1.76 | 0.0056 |
| 99887 | 103257_at | Tmem56 | transmembrane protein 56 | 0.82 | 1.76 | 0.00182 |
| 193740 | 93875_at | Hspa1a | heat shock protein 1A | 0.81 | 1.75 | 0.00693 |
| 69581 | 96747_at | Rhou | ras homolog gene family, member U | 0.80 | 1.74 | 0.0187 |
| 57264 | 102366_at | Retn | resistin | 0.79 | 1.73 | 0.0253 |
| AV349686 | 161610_at | Ndrg2 similar | N-myc Downstream regulated 2 | 0.70 | 1.62 | 0.0123 |
| 213742 | 99126_at | Xist | inactive X specific transcripts | 0.68 | 1.60 | 0.000767 |
| 104158 | 101539_f_at | Ces3 | carboxylesterase 3 | 0.66 | 1.58 | 0.000143 |
| 30956 | 103389_at | Aass | aminoadipate-semialdehyde synthase | 0.61 | 1.52 | 0.0113 |
| 18054 | 96153_at | Ngp | neutrophilic granule protein | 0.58 | 1.50 | 0.0389 |
| 20201 | 103448_at | S100a8 | S100 calcium binding protein A8 (calgranulin A) | 0.58 | 1.49 | 0.0434 |
| 22626 | 161149_r_at | Slc23a3 | solute carrier family 23 (nucleobase transporters), member 3 | 0.56 | 1.48 | 0.0255 |
| 14600 | 99108_s_at | Ghr | growth hormone receptor | 0.52 | 1.44 | 0.0389 |
| 235320 | 92202_g_at | Zbtb16 | zinc finger and BTB domain containing 16 | 0.51 | 1.42 | 0.0216 |
| 238257 | 96749_f_at | Tmem30b | transmembrane protein 30B | 0.47 | 1.38 | 0.0243 |
| 13106 | 93996_at | Cyp2e1 | cytochrome P450, family 2, subfamily e, polypeptide 1 | 0.42 | 1.33 | 0.0337 |

**B. Genes down-regulated in E-IAA positive PaLN**

| | | | | | | |
|---|---|---|---|---|---|---|
| 387524 | 99574_at | Znrf2 | zinc and ring finger 2 | -1.43 | -2.69 | 0.000000305 |

| | | | | | | |
|---|---|---|---|---|---|---|
| 380794 | 102824_g_at | Ighg | Immunoglobulin heavy chain (gamma polypeptide) | -1.11 | -2.17 | 0.00867 |
| 664944 | 102818_at | Slx, Xmr | Xmr protein | -1.10 | -2.14 | 0.0142 |
| 67203 | 94910_at | Nde1 | nuclear distribution gene E homolog 1 (A nidulans) | -1.08 | -2.12 | 0.00264 |
| 15312 | 96699_at | Hmgn1 | high mobility group nucleosomal binding domain 1 | -1.00 | -2.00 | 0.0469 |
| 16142 | 93638_s_at | Igl-V1 | immunoglobulin lambda chain, variable 1 | -0.97 | -1.96 | 0.0164 |
| 380795 | 102721_at | IgG1c | expressed sequence AI324046 | -0.89 | -1.85 | 0.0123 |
| 21974 | 99462_at | Top2b | topoisomerase (DNA) II beta | -0.86 | -1.81 | 0.000353 |
| 668838 | 100048_at | Rap1a | RAS-related protein-1a | -0.86 | -1.81 | 0.0418 |
| 230484 | 160234_at | Usp1 | ubiquitin specific peptdiase 1 | -0.85 | -1.80 | 0.0123 |
| 14017 | 98025_at | Evi2a | ecotropic viral integration site 2a | -0.84 | -1.79 | 0.0374 |
| 69774 | 97322_at | Ms4a6b | membrane-spanning 4-domains, subfamily A, member 6B | -0.83 | -1.78 | 0.00389 |
| 66949 | 99149_at | Trim59 | similar to mouse RING finger 1 | -0.83 | -1.77 | 0.0216 |
| 105439 | 160236_at | Slain1 | SLAIN motif family, member 1 | -0.82 | -1.76 | 0.0152 |
| 74326 | 97491_at | Hnrpr | heterogeneous nuclear ribonucleoprotein R | -0.81 | -1.76 | 0.0179 |
| 22194 | 92660_f_at | Ube2e1 | ubiquitin-conjugating enzyme E2E 1, UBC4/5 homolog (yeast) | -0.80 | -1.74 | 0.0156 |
| 100046793 | 96971_f_at | Similar to IgG | Immunoglobulin kappa chain variable 28 (V28) | -0.76 | -1.73 | 0.0116 |
| 18538 | 101065_at | Pcna | proliferating cell nuclear antigen | -0.79 | -1.73 | 0.0408 |
| 20728 | 103454_at | Spic | Spi-C transcription factor (Spi-1/PU.1 related) | -0.76 | -1.69 | 0.0064 |
| 54394 | 98415_at | Crlf3 | cytokine receptor-like factor 3 | -0.76 | -1.69 | 0.0439 |
| 14910 | 160620_at | Gt(ROSA)26Sor | gene trap ROSA 26, Philippe Soriano | -0.75 | -1.68 | 0.00501 |
| 234069 | 96545_s_at | Pcid2 | PCI domain containing 2 | -0.73 | -1.65 | 0.0141 |
| 13136 | 103617_at | Cd55 | CD55 antigen | -0.72 | -1.65 | 0.0251 |
| 15381 | 160199_at | Hnrpc | heterogeneous nuclear ribonucleoprotein C | -0.69 | -1.62 | 0.0355 |
| 50790 | 102381_at | Acsl4 | acyl-CoA synthetase long-chain family member 4 | -0.67 | -1.59 | 0.0355 |
| 13025 | 103518_at | Ctla2b | cytotoxic T lymphocyte-associated protein 2 beta | -0.64 | -1.56 | 0.0355 |
| 12479 | 103422_at | Cd1d1 | CD1d1 antigen | -0.62 | -1.53 | 0.0398 |
| 17918 | 98968_at | Myo5a | myosin Va | -0.58 | -1.49 | 0.0056 |

| | | | | | | |
|---|---|---|---|---|---|---|
| 27050 | 101137_at | Rps3 | ribosomal protein S3 | -0.52 | -1.44 | 0.0243 |
| 16149 | 101054_at | Cd74 | CD74 antigen (invariant polypeptide of major histocompatibility complex, class II antigen-associated) | -0.50 | -1.42 | 0.00298 |
| 20688 | 92992_i_at | Sp4 | trans-acting transcription factor 4 | -0.44 | -1.36 | 0.000425 |
| 100046552 | 93086_at | Cr1 | immunoglobulin kappa chain, constant region | -0.44 | -1.36 | 0.0000559 |
| 100041965 | 161360_at | Oaz1 | ornithine decarboxylase antizyme | -0.40 | -1.32 | 0.0305 |
| 13627 | 94766_at | Eef1a1 | eukaryotic translation elongation factor 1 alpha 1 | -0.33 | -1.25 | 0.00858 |

**C. Expressed Sequence Tags (EST)**

| | | | | | | |
|---|---|---|---|---|---|---|
| 67373 | 100103_f_at; 100104_r_at | RIKEN 2210010C04 | Trypsinogen 7 similar | 4.32 | 20.03 | 0 |
| 69036 | 160145_at | Splign similar EST unknown | RIKEN cDNA 1810010M01 gene | 5.40 | 42.36 | 0 |
| V00829 | 100681_f_at | MGK2 fragment--- | Similar to KlKb9--- | 3.84 | 14.27 | 0 |
| 73626 | 92712_at | TCRVb, 1810009J06Rik | RIKEN cDNA 1810009J06 gene | 1.99 | 3.96 | 0 |
| AV171666 | 161677_r_at | EST Unknown- | cDNA sequence BC003494 | 1.39 | 2.63 | 0.000000256 |
| AE000663 | 101336_at | EST unknown | TCR V beta locus EST or dopamine beta-hydroxylase type 1 | 1.18 | 2.26 | 0.000058 |
| AV250694 | 161131_r_at | EST | Unknown | 0.90 | 1.87 | 0.00528 |
| AW049643 | 160250_at | Sec11c Homologue EST | RIKEN 1810029G24 | 0.79 | 1.73 | 0.0384 |
| AV067171 | 161641_at | EST Unknown | cDNA sequence BC008155 | 0.65 | 1.57 | 0.0384 |
| 224171 | 160973_at | RTRtv | RIKEN C330027C09 | -0.53 | -1.44 | 0.0173 |
| 67922 | 97866_at | EST Unknown | RIKEN cDNA 2510049I19 gene | -0.72 | -1.64 | 0.0275 |
| 104457 | 95634_at | EST Unknown | RIKEN cDNA 0610010K14 gene | -1.08 | -2.11 | 0.00245 |
