# Supplementary material for: Early over expression of messenger RNA for multiple genes, including insulin, in the Pancreatic Lymph Nodes of NOD mice is associated with Islet Autoimmunity
Source: BMC Med Genomics. 2009 Oct 2;2:63. doi: 10.1186/1755-8794-2-63 (PMC2763872; doi:10.1186/1755-8794-2-63)
Supplement: Additional file 4 — Genes specifically expressed in the pancreas but not found to be expressed in the E-IAA PLN transcriptome. [file 1755-8794-2-63-S4.PDF]

Genes specifically expressed in the pancreas and not found to be expressed in the E-IAA PLN transcriptome.

Site of expression	Gene symbol	Probe set (Affymetrix)
**Membranes of β cells**<br>*Glucose transporters*	*Slc2a4, Slc2a2, Slc2a3*	102314_at, 103357_at, 92292_at
**Exocrine pancreas**	*Gata-4, otc, nestin, Isl-1*	102713_at; 94414_at & 114938_at, 103549_at, 92515_at
**Developing pancreas**<br>*Neurogenin*	*Ngn1, Ngn2, Ngn3*	161069_at & 93230_at, 97792_at, 99814_at
**Endocrine pancreas**	*Gcgr, Gcg, Smst, Iapp*	103498_at, 94633_at, 95436_at, 99488_at
