# Supplementary material for: Early over expression of messenger RNA for multiple genes, including insulin, in the Pancreatic Lymph Nodes of NOD mice is associated with Islet Autoimmunity
Source: BMC Med Genomics. 2009 Oct 2;2:63. doi: 10.1186/1755-8794-2-63 (PMC2763872; doi:10.1186/1755-8794-2-63)
Supplement: Additional file 5 — Functional categories of genes and ESTs as annotated by GO and PANTHER tools. Molecular functions and Biological process terms are as described in the text for Table 2. [file 1755-8794-2-63-S5.PDF]

Functional categories of genes and ESTs as annotated by GO and PANTHER tools. Molecular functions and Biological process terms are as described in the text for Table 2 in the text. Genes may belong to more than one category.

Up-regulated genes

Molecular Function

Catalytic activity

AFFY ID	LOCUSLINK ENTREZ_ID	GENENAME	GENE_SYMBOL
100060_I_AT	16622	KALLIKREIN 1-RELATED PEPTIDASE B5	KLK1B5
100061_F_AT	16612	KALLIKREIN 1	KLK1
100103_F_AT; 100104_R_AT	67373	RIKEN CDNA 2210010C04 GENE	2210010C04RIK
100719_F_AT	16615	KALLIKREIN 1-RELATED PEPTIDASE B16	KLK1B16
101009_AT	16691	KERATIN COMPLEX 2, BASIC, GENE 8	KRT8
101043_F_AT	435889	TRYPSIN 4	1810049H19RIK
101058_AT	11722	AMYLASE 1, SALIVARY	AMY1
101082_AT	17436	MALIC ENZYME, SUPERNATANT	ME1
101289_F_AT	13646	KALLIKREIN 1-RELATED PEPTIDASE B22	KLK1B22
101338_F_AT	386551	TRY10-LIKE TRYPSINOGEN	EG386551
101339_AT	114228	PROTEASE, SERINE, 1 (TRYPSIN 1)	PRSS1
101408_AT	14431	GUANIDINOACETATE METHYLTRANSFERASE	GAMT
101539_F_AT	104158	CARBOXYLESTERASE 3	CES3
101638_S_AT	13114	CYTOCHROME P450, FAMILY 3, SUBFAMILY A, POLYPEPTIDE 16	CYP3A16; CYP3A11
101985_AT	18815	PLASMINOGEN	PLG
102693_F_AT	16618	KALLIKREIN 1-RELATED PETIDASE B26	KLK1B26
103023_AT	16816	LECITHIN CHOLESTEROL ACYLTRANSFERASE	LCAT
103389_AT	30956	AMINOADIPATE-SEMIALDEHYDE SYNTHASE	AASS
103531_F_AT	67475	ERO1-LIKE BETA (S. CEREVISIAE)	ERO1LB
103562_F_AT	433762	SIMILAR TO RETROVIRUS-RELATED POL POLYPROTEIN (ENDONUCLEASE)	LOC433762
103882_AT	93721	CARBOXYPEPTIDASE N	CPN1
104495_F_AT	16622	KALLIKREIN 1-RELATED PEPTIDASE B5	KLK1B5
160070_AT	18947	PANCREATIC LIPASE-RELATED PROTEIN 2	PNLIPRP2
160083_AT	16956	LIPOPROTEIN LIPASE	LPL
160120_I_AT	18778	PHOSPHOLIPASE A2, GROUP IB, PANCREAS	PLA2G1B
160250_AT	66286	SEC11-LIKE 3 (S. CEREVISIAE)	SEC11C
160350_AT	14874	GLUTATHIONE TRANSFERASE ZETA 1 (MALEYLACETOACETATE ISOMERASE)	GSTZ1
160375_AT	12350	CARBONIC ANHYDRASE 3	CAR3
160421_R_AT	66473	CHYMOTRYPSINOGEN B1	CTRB1
160481_AT	18534	PHOSPHOENOLPYRUVATE CARBOXYKINASE 1, CYTOSOLIC	PCK1
160628_AT	26912	GLYCINE C-ACETYLTRANSFERASE (2-AMINO-3-KETOBUTYRATE-COENZYME A LIGASE)	GCAT
160639_AT	15451	HEPSIN	HPN
160744_R_AT	109660	RIKEN CDNA 1810004D15 GENE	CTRL
161131_R_AT	228889	DEAD (ASP-GLU-ALA-ASP) BOX	DDX27

		POLYPEPTIDE 27	
161221_F_AT	27053	ASPARAGINE SYNTHETASE	ASNS
161637_F_AT	16618	KALLIKREIN 1-RELATED PETIDASE B26	KLK1B26; KLK1; KLK1B5
161677_R_AT	80294	RIKEN CDNA 2310011G23 GENE	POFUT2
92601_AT	18946	PANCREATIC LIPASE RELATED PROTEIN 1	PNLIPRP1
92712_AT	73626	RIKEN CDNA 1810009J06 GENE	1810009J06RIK
92768_S_AT	11656	AMINOLEVULINIC ACID SYNTHASE 2, ERYTHROID	ALAS2
92873_F_AT	22072	PROTEASE, SERINE, 2	PRSS2
93208_AT	114228	PROTEASE, SERINE, 1 (TRYPSIN 1)	PRSS1; PRSS3
93575_AT	14590	GAMMA-GLUTAMYL HYDROLASE	GGH
93783_AT	109901	ELASTASE 1, PANCREATIC	ELA1
93996_AT	13106	CYTOCHROME P450, FAMILY 2, SUBFAMILY E, POLYPEPTIDE 1	CYP2E1
94037_AT	13706	ELASTASE 2A	ELA2
94392_F_AT	11727	ANGIOGENIN, RIBONUCLEASE A FAMILY, MEMBER 1	ANG
94716_F_AT; 94773_AT; 95775_F_AT	16623	KALLIKREIN 1-RELATED PEPTIDASE B1	KLK1B1; KLK1B4; KLK1B9; KLK1
94778_AT	26358	ALDEHYDE DEHYDROGENASE FAMILY 1, SUBFAMILY A7	ALDH1A7
95133_AT	27053	ASPARAGINE SYNTHETASE	ASNS
95407_AT	18478	PHENYLALANINE HYDROXYLASE	PAH
95611_AT	16956	LIPOPROTEIN LIPASE	LPL
96038_AT	58809	RIBONUCLEASE, RNASE A FAMILY 4	RNASE4
96336_AT	67092	GLYCINE AMIDINOTRANSFERASE (L-ARGININE:GLYCINE AMIDINOTRANSFERASE)	GATM
96346_AT	12583	CYSTEINE DIOXYGENASE 1, CYTOSOLIC	CDO1
96747_AT	69581	RAS HOMOLOG GENE FAMILY, MEMBER U	RHOU
96828_AT	14711	GLYCINE N-METHYLTRANSFERASE	GNMT
97450_S_AT	110695	DNA SEGMENT, CHR 18, WAYNE STATE UNIVERSITY 181, EXPRESSED	ALDH7A1
97523_I_AT; 97524_F_AT	11723	AMYLASE 2, PANCREATIC	AMY2
97964_AT	66120	FK506 BINDING PROTEIN 11	FKBP11
98041_AT	19752	RIBONUCLEASE, RNASE A FAMILY, 1 (PANCREATIC)	RNASE1
98549_AT	22370	VITRONECTIN	VTN
99056_AT	13180	PTERIN 4 ALPHA CARBINOLAMINE DEHYDRATASE/DIMERIZATION COFACTOR OF HEPATOCYTE NUCLEAR FACTOR 1 ALPHA (TCF1) 1	PCBD1
99671_AT	11537	COMPLEMENT FACTOR D (ADIPSIN)	CFD
99939_AT	12613	CARBOXYL ESTER LIPASE	CEL

Serine-type peptidase activity

AFFY ID	LOCUSLINK ENTREZ_ID	GENENAME	GENE_SYMBOL
100060_i_at, 161637_f_at, 104495_f_at	16622	KALLIKREIN 1-RELATED PEPTIDASE B5	KLK1B5; KLK1
100060_i_at, 161637_f_at,	16612	KALLIKREIN 1	KLK1

94716_f_at, 100061_f_at 100103_f_at, 100104_r_at	67373	RIKEN CDNA 2210010C04 GENE	2210010C04RIK
100719_f_at	16615	KALLIKREIN 1-RELATED PEPTIDASE B16	KLK1B16
101043_f_at, 101339_at, 101338_f_at	435889	TRYPSIN 4	1810049H19RIK
101289_f_at	13646	KALLIKREIN 1-RELATED PEPTIDASE B22	KLK1B22
101338_f_at	386551	TRY10-LIKE TRYPSINOGEN	EG386551
101339_at, 93208_at	114228	PROTEASE, SERINE, 3	PRSS1; PRSS3
101985_at	18815	PLASMINOGEN	PLG
102693_f_at, 161637_f_at	16618	KALLIKREIN 1-RELATED PETIDASE B26	KLK1B26; KLK1; KLK1B5
160421_r_at	66473	CHYMOTRYPSINOGEN B1	CTRB1
160639_at	15451	HEPSIN	HPN
160744_r_at	109660	RIKEN CDNA 1810004D15 GENE	CTRL
92712_at	73626	RIKEN CDNA 1810009J06 GENE	92712_AT
92873_f_at	22072	PROTEASE, SERINE, 2	1810009J06RIK
93783_at	109901	ELASTASE 1, PANCREATIC	ELA1
94037_at	13706	ELASTASE 2A	ELA2
94716_f_at, 94773_at	16623	KALLIKREIN 1-RELATED PEPIDASE B4	KLK1B1; KLK1B4; KLK1B9; KLK1
94716_f_at, 95775_f_at	18048	KALLIKREIN 1-RELATED PEPTIDASE B1	KLKB4
99671_at	11537	COMPLEMENT FACTOR D (ADIPSIN)	CFD

Actin Binding

AFFY ID	LOCUSLINK ENTREZ_ID	GENENAME	GENE_SYMBOL
100086_AT	16976	LOW DENSITY LIPOPROTEIN RECEPTOR-RELATED PROTEIN ASSOCIATED PROTEIN 1	LRPAP1
101638_S_AT	13114	CYTOCHROME P450, FAMILY 3, SUBFAMILY A, POLYPEPTIDE 16	CYP3A16
101638_S_AT	13112	CYTOCHROME P450, FAMILY 3, SUBFAMILY A, POLYPEPTIDE 11	CYP3A11
160350_AT	14874	GLUTATHIONE TRANSFERASE ZETA 1 (MALEYLACETOACETATE ISOMERASE)	GSTZ1
161149_R_AT	22626	SOLUTE CARRIER FAMILY 23 (NUCLEOBASE TRANSPORTERS), MEMBER 3	SLC23A3
92873_F_AT	22072	PROTEASE, SERINE, 2	PRSS2
93575_AT	14590	GAMMA-GLUTAMYL HYDROLASE	GGH
93783_AT	109901	ELASTASE 1, PANCREATIC	ELA1
93875_AT	193740	HEAT SHOCK PROTEIN 1B	HSPA1B
94777_AT	11657	ALBUMIN 1	ALB
95407_AT	18478	PHENYLALANINE HYDROXYLASE	PAH
96064_AT	19695	REGENERATING ISLET-DERIVED 3 GAMMA	REG3G
96153_AT	18054	NEUTROPHILIC GRANULE PROTEIN	NGP
96747_AT	69581	RAS HOMOLOG GENE FAMILY, MEMBER U	RHOU
98549_AT	22370	VITRONECTIN	VTN
99862_AT	11625	ALPHA-2-HS-GLYCOPROTEIN	AHSG

Calcium binding

AFFY ID	LOCUSLINK ENTREZ_ID	GENENAME	GENE_SYMBOL
103448_AT	20201	S100 CALCIUM BINDING PROTEIN A8 (CALGRANULIN A)	S100A8
103887_AT	20202	S100 CALCIUM BINDING PROTEIN A9 (CALGRANULIN B)	S100A9
160083_AT	16956	LIPOPROTEIN LIPASE	LPL
160481_AT	18534	PHOSPHOENOLPYRUVATE CARBOXYKINASE 1, CYTOSOLIC	PCK1
95407_AT	18478	PHENYLALANINE HYDROXYLASE	PAH
95611_AT	16956	LIPOPROTEIN LIPASE	LPL
97964_AT	66120	FK506 BINDING PROTEIN 11	FKBP11

Vitamin binding

AFFY ID	LOCUSLINK ENTREZ_ID	GENENAME	GENE_SYMBOL
160628_AT	26912	GLYCINE C-ACETYLTRANSFERASE (2-AMINO-3-KETOBUTYRATE-COENZYME A LIGASE)	GCAT
92768_S_AT	11656	AMINOLEVULINIC ACID SYNTHASE 2, ERYTHROID	ALAS2
94777_AT	11657	ALBUMIN 1	ALB
96047_AT	19662	RETINOL BINDING PROTEIN 4, PLASMA	RBP4
96828_AT	14711	GLYCINE N-METHYLTRANSFERASE	GNMT
99197_AT	14473	GROUP SPECIFIC COMPONENT	GC

Transferase activity, transferring nitrogenous groups

AFFY ID	LOCUSLINK ENTREZ_ID	GENENAME	GENE_SYMBOL
160628_AT	26912	GLYCINE C-ACETYLTRANSFERASE (2-AMINO-3-KETOBUTYRATE-COENZYME A LIGASE)	GCAT
92768_S_AT	11656	AMINOLEVULINIC ACID SYNTHASE 2, ERYTHROID	ALAS2
96336_AT	67092	GLYCINE AMIDINOTRANSFERASE (L-ARGININE:GLYCINE AMIDINOTRANSFERASE)	GATM

Tissue Kallikrein activity

AFFY ID	LOCUSLINK ENTREZ_ID	GENENAME	GENE_SYMBOL
104495_F_AT; 100060_I_AT; 161637_F_AT	16622	KALLIKREIN 1-RELATED PEPTIDASE B5	KLK1B5
100060_I_AT; 100061_F_AT; 94716_F_AT	16612	KALLIKREIN 1	KLK1
101289_F_AT	13646	KALLIKREIN 1-RELATED PEPTIDASE B22	KLK1B22
102693_F_AT; 161637_F_AT	16618	KALLIKREIN 1-RELATED PETIDASE B26	KLK1B26
94716_F_AT; 95775_F_AT	16623	KALLIKREIN 1-RELATED PEPTIDASE B1	KLKB1
94716_F_AT; 94773_AT	18050	KALLIKREIN 1-RELATED PEPIDASE B4	KLK1B4
94716_F_AT	13648	KALLIKREIN 1-RELATED PEPTIDASE B9	KLK1B9
94716_F_AT	76999	RIKEN CDNA 1700127D06 GENE	1700127D06RIK
94773_AT	18048	KALLIKREIN 1-RELATED PEPIDASE B4	KLK1B4

Biological Process

Metabolic process

AFFY ID	LOCUSLINK	GENENAME	GENE_SYMBOL

	ENTREZ_ID		
94778_AT	26358	ALDEHYDE DEHYDROGENASE FAMILY 1, SUBFAMILY A7	ALDH1A7
103389_AT	30956	AMINOADIPATE-SEMIALDEHYDE SYNTHASE	AASS
92768_S_AT	11656	AMINOLEVULINIC ACID SYNTHASE 2, ERYTHROID	ALAS2
101058_AT	11722	AMYLASE 1, SALIVARY	AMY1
97523_I_AT; 97524_F_AT	11723	AMYLASE 2, PANCREATIC	AMY2
94392_F_AT	11727	ANGIOGENIN, RIBONUCLEASE A FAMILY, MEMBER 1	ANG
161221_F_AT; 95133_AT	27053	ASPARAGINE SYNTHETASE	ASNS
160375_AT	12350	CARBONIC ANHYDRASE 3	CAR3
99939_AT	12613	CARBOXYL ESTER LIPASE	CEL
101539_F_AT	104158	CARBOXYLESTERASE 3	CES3
103882_AT	93721	CARBOXYPEPTIDASE N	CPN1
160421_R_AT	66473	CHYMOTRYPSINOGEN B1	CTRB1
104143_AT	56358	COATOMER PROTEIN COMPLEX, SUBUNIT ZETA 2	COPZ2
162276_I_AT	12260	COMPLEMENT COMPONENT 1, Q SUBCOMPONENT, BETA POLYPEPTIDE	C1QB
99671_AT	11537	COMPLEMENT FACTOR D (ADIPSIN)	CFD
162196_F_AT	16433	CUB AND ZONA PELLUCIDA-LIKE DOMAINS 1	CUZD1
96346_AT	12583	CYSTEINE DIOXYGENASE 1, CYTOSOLIC	CDO1
93996_AT	13106	CYTOCHROME P450, FAMILY 2, SUBFAMILY E, POLYPEPTIDE 1	CYP2E1
101638_S_AT	13112	CYTOCHROME P450, FAMILY 3, SUBFAMILY A, POLYPEPTIDE 11 or 16	CYP3A11, CYP3A16
97450_S_AT	110695	DNA SEGMENT, CHR 18, WAYNE STATE UNIVERSITY 181, EXPRESSED	ALDH7A1
93783_AT	109901	ELASTASE 1, PANCREATIC	ELA1
94037_AT	13706	ELASTASE 2A	ELA2
102774_AT	13645	EPIDERMAL GROWTH FACTOR	EGF
103531_F_AT	67475	ERO1-LIKE BETA (S. CEREVISIAE)	ERO1LB
161677_R_AT	80294	EXPRESSED SEQUENCE AI256847	POFUT2
97964_AT	66120	FK506 BINDING PROTEIN 11	FKBP11
98324_AT	15377	FORKHEAD BOX A3	FOXA3
93575_AT	14590	GAMMA-GLUTAMYL HYDROLASE	GGH
160350_AT	14874	GLUTATHIONE TRANSFERASE ZETA 1 (MALEYLACETOACETATE ISOMERASE)	GSTZ1
96336_AT	67092	GLYCINE AMIDINOTRANSFERASE (L-ARGININE:GLYCINE AMIDINOTRANSFERASE)	GATM
160628_AT	26912	GLYCINE C-ACETYLTRANSFERASE (2-AMINO-3-KETOBUTYRATE-COENZYME A LIGASE)	GCAT
99197_AT	14473	GROUP SPECIFIC COMPONENT	GC
101408_AT	14431	GUANIDINOACETATE METHYLTRANSFERASE	GAMT
93875_AT	193740	HEAT SHOCK PROTEIN 1B	HSPA1B
160639_AT	15451	HEPSIN	HPN
97658_F_AT; 97659_R_AT	16333	INSULIN I	INS1
100150_F_AT;	16334	INSULIN II	INS2

100150_F_AT; 98467_AT	16427	INTER ALPHA-TRYPSIN INHIBITOR, HEAVY CHAIN 4	ITIH4
104519_AT	16425	INTER-ALPHA TRYPSIN INHIBITOR, HEAVY CHAIN 2	ITIH2
100061_F_AT; 94716_F_AT	16612	KALLIKREIN 1	KLK1
94773_AT	18048	KALLIKREIN 1-RELATED PEPIDASE B4	KLK1B4
95775_F_AT	16623	KALLIKREIN 1-RELATED PEPTIDASE B1	KLK1B1
100719_F_AT	16615	KALLIKREIN 1-RELATED PEPTIDASE B16	KLK1B16
101289_F_AT	13646	KALLIKREIN 1-RELATED PEPTIDASE B22	KLK1B22
100060_I_AT; 104495_F_AT; 161637_F_AT	16622	KALLIKREIN 1-RELATED PEPTIDASE B5	KLK1B5
94716_F_AT	18050	KALLIKREIN 1-RELATED PEPTIDASE B9	KLK1B9
102693_F_AT; 161637_F_AT	16618	KALLIKREIN 1-RELATED PETIDASE B26	KLK1B26
101009_AT	16691	KERATIN COMPLEX 2, BASIC, GENE 8	KRT8
103023_AT	16816	LECITHIN CHOLESTEROL ACYLTRANSFERASE	LCAT
160083_AT; 95611_AT	16956	LIPOPROTEIN LIPASE	LPL
101082_AT	17436	MALIC ENZYME, SUPERNATANT	ME1
92601_AT	18946	PANCREATIC LIPASE RELATED PROTEIN 1	PNLIPRP1
160070_AT	18947	PANCREATIC LIPASE-RELATED PROTEIN 2	PNLIPRP2
95407_AT	18478	PHENYLALANINE HYDROXYLASE	PAH
160481_AT	18534	PHOSPHOENOLPYRUVATE CARBOXYKINASE 1, CYTOSOLIC	PCK1
160120_I_AT	18778	PHOSPHOLIPASE A2, GROUP IB, PANCREAS	PLA2G1B
101985_AT	18815	PLASMINOGEN	PLG
93208_AT; 101339_AT	547443	PROTEASE, SERINE, 1 (TRYPSIN 1)	PRSS1
92873_F_AT	22072	PROTEASE, SERINE, 2	PRSS2
101339_AT; 93208_AT	22073	PROTEASE, SERINE, 3; TRYPSINOGEN 12	PRSS3
99056_AT	13180	PTERIN 4 ALPHA CARBINOLAMINE DEHYDRATASE/DIMERIZATION COFACTOR OF HEPATOCYTE NUCLEAR FACTOR 1 ALPHA (TCF1) 1	PCBD1
160744_R_AT	109660	RIKEN CDNA 1810004D15 GENE	CTRL
92712_AT	73626	RIKEN CDNA 1810009J06 GENE	1810009J06RIK
160132_AT	109791	RIKEN CDNA 2200003J09 GENE	CLPS
100103_F_AT; 100104_R_AT	67373	RIKEN CDNA 2210010C04 GENE	2210010C04RIK
103887_AT	20202	S100 CALCIUM BINDING PROTEIN A9 (CALGRANULIN B)	S100A9
160250_AT	66286	SEC11-LIKE 3 (S. CEREVISIAE)	SEC11C
101339_AT	545562	SIMILAR TO ANIONIC TRYPSIN II PRECURSOR (PRETRYPSINOGEN II)	OTTMUSG00000022462
103562_F_AT	433762	SIMILAR TO RETROVIRUS-RELATED POL POLYPROTEIN (ENDONUCLEASE);	LOC433762
101338_F_AT	386551	TRY10-LIKE TRYPSINOGEN	EG386551
101043_F_AT; 101339_AT;	435889	TRYPSIN 4	1810049H19RIK

101338_F_AT			
92202_G_AT	235320	ZINC FINGER AND BTB DOMAIN CONTAINING 16	ZBTB16

Immune function GO & PANTHER

AFFY ID	LOCUSLINK ENTREZ_ID	GENENAME	GENE_SYMBOL
101578_F_AT; 95705_S_AT	11461	ACTIN, BETA, CYTOPLASMIC	ACTB
94777_AT	11657	ALBUMIN 1	ALB
99862_AT	11625	ALPHA-2-HS-GLYCOPROTEIN	AHSG
103389_AT	30956	AMINOADIPATE-SEMIALDEHYDE SYNTHASE	AASS
92768_S_AT	11656	AMINOLEVULINIC ACID SYNTHASE 2, ERYTHROID	ALAS2
101058_AT	11722	AMYLASE 1, SALIVARY	AMY1
97523_I_AT	11723	AMYLASE 2, PANCREATIC	AMY2
94392_F_AT	11727	ANGIOGENIN, RIBONUCLEASE A FAMILY, MEMBER 1	ANG
101539_F_AT	104158	CARBOXYLESTERASE 3	CES3
162276_I_AT	12260	COMPLEMENT COMPONENT 1, Q SUBCOMPONENT, BETA POLYPEPTIDE	C1QB
99671_AT	11537	COMPLEMENT FACTOR D (ADIPSIN)	CFD
101638_S_AT	13114	CYTOCHROME P450, FAMILY 3, SUBFAMILY A, POLYPEPTIDE 16	CYP3A16; CYP3A11
161046_AT	12931	CYTOKINE RECEPTOR-LIKE FACTOR 1	CRLF1
94037_AT	13706	ELASTASE 2A	ELA2
102774_AT	13645	EPIDERMAL GROWTH FACTOR	EGF
101553_AT	14161	FIBRINOGEN, ALPHA POLYPEPTIDE	FGA
98324_AT	15377	FORKHEAD BOX A3	FOXA3
98423_AT	14619	GAP JUNCTION MEMBRANE CHANNEL PROTEIN BETA 2	GJB2
160350_AT	14874	GLUTATHIONE TRANSFERASE ZETA 1 (MALEYLACETOACETATE ISOMERASE)	GSTZ1
93875_AT	193740	HEAT SHOCK PROTEIN 1B	HSPA1B
162457_F_AT; 94781_AT	15122	HEMOGLOBIN ALPHA, ADULT CHAIN 1	HBA-A1
101869_S_AT; 103534_AT	15127	HEMOGLOBIN BETA CHAIN COMPLEX	HBB-B2
100150_F_AT	16334	INSULIN II	INS2
98467_AT	16427	INTER ALPHA-TRYPSIN INHIBITOR, HEAVY CHAIN 4	ITIH4
104519_AT	16425	INTER-ALPHA TRYPSIN INHIBITOR, HEAVY CHAIN 2	ITIH2
94716_F_AT	16612	KALLIKREIN 1	KLK1
94716_F_AT; 94773_AT	18048	KALLIKREIN 1-RELATED PEPIDASE B4	KLK1B4
95775_F_AT	16623	KALLIKREIN 1-RELATED PEPTIDASE B1	KLK1B1
100719_F_AT	16615	KALLIKREIN 1-RELATED PEPTID B16	KLK1B16
94716_F_AT	16623	KALLIKREIN 1-RELATED PEPTIDASE B9	KLK1B9
94270_AT	16668	KERATIN COMPLEX 1, ACIDIC, GENE 18	KRT18
101009_AT	16691	KERATIN COMPLEX 2, BASIC, GENE 8	KRT8
95611_AT	16956	LIPOPROTEIN LIPASE	LPL
100086_AT	16976	LOW DENSITY LIPOPROTEIN RECEPTOR-RELATED PROTEIN ASSOCIATED PROTEIN 1	LRPAP1
101082_AT	17436	MALIC ENZYME, SUPERNATANT	ME1
93573_AT	17748	METALLOTHIONEIN 1	MT1
101561_AT	17750	METALLOTHIONEIN 2	MT2

102918_AT	17829	MUCIN 1, TRANSMEMBRANE	MUC1
96153_AT	18054	NEUTROPHILIC GRANULE PROTEIN	NGP
102169_AT	258051	OLFACTORY RECEPTOR 93	OLFR93
160070_AT	18947	PANCREATIC LIPASE-RELATED PROTEIN 2	PNLIPRP2
161890_F_AT; 96009_S_AT	18489	PANCREATITIS-ASSOCIATED PROTEIN	PAP
160481_AT	18534	PHOSPHOENOLPYRUVATE CARBOXYKINASE 1, CYTOSOLIC	PCK1
160120_I_AT	18778	PHOSPHOLIPASE A2, GROUP IB, PANCREAS	PLA2G1B
101985_AT	18815	PLASMINOGEN	PLG
96588_AT	19218	PROSTAGLANDIN E RECEPTOR 3 (EP3)	PTGER3
95786_AT	19693	REGENERATING ISLET-DERIVED 2	REG2
103954_AT; 161642_F_AT	19694	REGENERATING ISLET-DERIVED 3 ALPHA	REG3A
96064_AT	19695	REGENERATING ISLET-DERIVED 3 GAMMA	REG3G
96038_AT	58809	RIBONUCLEASE, RNASE A FAMILY 4	RNASE4
98041_AT	19752	RIBONUCLEASE, RNASE A FAMILY, 1 (PANCREATIC)	RNASE1
103562_F_AT	67527	RIKEN CDNA 1300007C21 GENE	LOC67527
94716_F_AT	76999	RIKEN CDNA 1700127D06 GENE	1700127D06RIK
160744_R_AT	109660	RIKEN CDNA 1810004D15 GENE	CTRL
160145_AT	69036	RIKEN CDNA 1810010M01 GENE	1810010M01RIK
161677_R_AT	80294	RIKEN CDNA 2310011G23 GENE	POFUT2
103257_AT	99887	RIKEN CDNA 4930577M16 GENE	TMEM56
94247_AT	109620	RIKEN CDNA 5730453H04 GENE	DSP
103448_AT	20201	S100 CALCIUM BINDING PROTEIN A8 (CALGRANULIN A)	S100A8
103887_AT	20202	S100 CALCIUM BINDING PROTEIN A9 (CALGRANULIN B)	S100A9
97519_AT	20750	SECRETED PHOSPHOPROTEIN 1	SPP1
97523_I_AT	545562	SIMILAR TO PANCREATIC ALPHA-AMYLASE PRECURSOR (PA) (1,4-ALPHA-D-GLUCAN GLUCANOHYDROLASE)	OTTMUSG00000022462
103562_F_AT	433762	SIMILAR TO RETROVIRUS-RELATED POL POLYPROTEIN (ENDONUCLEASE)	LOC433762
160306_AT	21835	THYROID HORMONE RESPONSIVE SPOT14 HOMOLOG (RATTUS)	THRSP
98549_AT	22370	VITRONECTIN	VTN
92202_G_AT	235320	ZINC FINGER AND BTB DOMAIN CONTAINING 16	ZBTB16

Proteolysis

AFFY ID	LOCUSLINK ENTREZ_ID	GENENAME	GENE_SYMBOL
101339_AT; 93208_AT	547443	TRYPSINOGEN 12	EG436523
101043_F_AT; 101338_F_AT	435889	TRYPSIN 4	1810049H19RIK
101338_F_AT	386551	TRY10-LIKE TRYPSINOGEN	EG386551
160250_AT	66286	SEC11-LIKE 3 (S. CEREVISIAE)	SEC11C
100103_F_AT	67373	RIKEN CDNA 2210010C04 GENE	2210010C04RIK
92712_AT	73626	RIKEN CDNA 1810009J06 GENE	1810009J06RIK
160744_R_AT	109660	RIKEN CDNA 1810004D15 GENE	CTRL
94716_F_AT	76999	RIKEN CDNA 1700127D06 GENE	1700127D06RIK
101339_AT; 93208_AT	22073	PROTEASE, SERINE, 3	PRSS3

92873_F_AT	22072	PROTEASE, SERINE, 2	PRSS2
101339_AT; 93208_AT	114228	PROTEASE, SERINE, 1 (TRYPSIN 1)	PRSS1
101985_AT	18815	PLASMINOGEN	PLG
102693_F_AT; 161637_F_AT	16618	KALLIKREIN 1-RELATED PETIDASE B26	KLK1B26
94716_F_AT	13648	KALLIKREIN 1-RELATED PEPTIDASE B9	KLK1B9
100060_I_AT; 104495_F_AT; 161637_F_AT	16622	KALLIKREIN 1-RELATED PEPTIDASE B5	KLK1B5
101289_F_AT	13646	KALLIKREIN 1-RELATED PEPTIDASE B22	KLK1B22
100719_F_AT	16615	KALLIKREIN 1-RELATED PEPTIDASE B16	KLK1B16
95775_F_AT; 94716_F_AT	16623	KALLIKREIN 1-RELATED PEPTIDASE B1	KLK1B1
94716_F_AT; 94773_AT	18048	KALLIKREIN 1-RELATED PEPIDASE B4	KLK1B4
100061_F_AT; 161637_F_AT; 94716_F_AT	16612	KALLIKREIN 1	KLK1
160639_AT	15451	HEPSIN	HPN
94037_AT	13706	ELASTASE 2A	ELA2
93783_AT	109901	ELASTASE 1, PANCREATIC	ELA1
99671_AT	11537	COMPLEMENT FACTOR D (ADIPSIN)	CFD
162276_I_AT	12260	COMPLEMENT COMPONENT 1, Q SUBCOMPONENT, BETA POLYPEPTIDE	C1QB
160421_R_AT	66473	CHYMOTRYPSINOGEN B1	CTRB1
103882_AT	93721	CARBOXYPEPTIDASE N	CPN1

Cell structure

AFFY ID	LOCUSLINK ENTREZ_ID	GENENAME	GENE_SYMBOL
101009_AT	16691	KERATIN COMPLEX 2, BASIC, GENE 8	KRT8
101338_F_AT	386551	TRY10-LIKE TRYPSINOGEN	EG386551
101339_AT; 93208_AT	11422817:1322073	PROTEASE, SERINE, 1/3 (TRYPSIN 1)	PRSS1; PRSS3
101578_F_AT; 95705_S_AT	11461	ACTIN, BETA, CYTOPLASMIC	ACTB
103023_AT	16816	LECITHIN CHOLESTEROL ACYLTRANSFERASE	LCAT
103531_F_AT	67475	ERO1-LIKE BETA (S. CEREVISIAE)	ERO1LB
103954_AT; 161642_F_AT	19694	REGENERATING ISLET-DERIVED 3 ALPHA	REG3A
160070_AT	18947	PANCREATIC LIPASE-RELATED PROTEIN 2	PNLIPRP2
160083_AT; 95611_AT	16956	LIPOPROTEIN LIPASE	LPL
160213_AT; 162312_F_AT	19692	REGENERATING ISLET-DERIVED 1	REG1
160481_AT	18534	PHOSPHOENOLPYRUVATE CARBOXYKINASE 1, CYTOSOLIC	PCK1
161149_R_AT	22626	SOLUTE CARRIER FAMILY 23 (NUCLE-OBASE TRANSPORTERS), MEMBER 3	SLC23A3
161221_F_AT; 95133_AT	27053	ASPARAGINE SYNTHETASE	ASNS
161641_AT	214917	CDNA SEQUENCE BC008155	BC008155
161677_R_AT	80294	RIKEN CDNA 2310011G23 GENE	POFUT2
162457_F_AT; 94781_AT	15122	HEMOGLOBIN ALPHA, ADULT CHAIN 1	HBA-A1

92252_AT	12425	CHOLECYSTOKININ A RECEPTOR	CCKAR
92871_AT	20338	SEL1 (SUPPRESSOR OF LIN-12) 1 HOMOLOG (C. ELEGANS)	SEL1L
94247_AT	109620	RIKEN CDNA 5730453H04 GENE	DSP
94270_AT	16668	KERATIN COMPLEX 1, ACIDIC, GENE 18	KRT18
94777_AT	11657	ALBUMIN 1	ALB
96336_AT	67092	GLYCINE AMIDINOTRANSFERASE (L-ARGININE:GLYCINE AMIDINOTRANSFERASE)	GATM
97450_S_AT	110695	DNA SEGMENT, CHR 18, WAYNE STATE UNIVERSITY 181, EXPRESSED	ALDH7A1
97519_AT	20750	SECRETED PHOSPHOPROTEIN 1	SPP1
98423_AT	14619	GAP JUNCTION MEMBRANE CHANNEL PROTEIN BETA 2	GJB2
99862_AT	11625	ALPHA-2-HS-GLYCOPROTEIN	AHSG

Lipid, fatty acid & steroid metabolism

AFFY ID	LOCUSLINK ENTREZ_ID	GENENAME	GENE_SYMBOL
101638_S_AT	13114	CYTOCHROME P450, FAMILY 3, SUBFAMILY A, POLYPEPTIDE 16	CYP3A16
101638_S_AT	13112	CYTOCHROME P450, FAMILY 3, SUBFAMILY A, POLYPEPTIDE 11	CYP3A11
101985_AT	18815	PLASMINOGEN	PLG
103023_AT	16816	LECITHIN CHOLESTEROL ACYLTRANSFERASE	LCAT
160070_AT	18947	PANCREATIC LIPASE-RELATED PROTEIN 2	PNLIPRP2
160083_AT	16956	LIPOPROTEIN LIPASE	LPL
160120_I_AT	18778	PHOSPHOLIPASE A2, GROUP IB, PANCREAS	PLA2G1B
160132_AT	109791	RIKEN CDNA 2200003J09 GENE	CLPS
160306_AT	21835	THYROID HORMONE RESPONSIVE SPOT14 HOMOLOG (RATTUS)	THRSP
92601_AT	18946	PANCREATIC LIPASE RELATED PROTEIN 1	PNLIPRP1
93575_AT	14590	GAMMA-GLUTAMYL HYDROLASE	GGH
93783_AT	109901	ELASTASE 1, PANCREATIC	ELA1
93996_AT	13106	CYTOCHROME P450, FAMILY 2, SUBFAMILY E, POLYPEPTIDE 1	CYP2E1
95611_AT	16956	LIPOPROTEIN LIPASE	LPL
98169_S_AT	14365	FRIZZLED HOMOLOG 3 (DROSOPHILA)	FZD3
99939_AT	12613	CARBOXYL ESTER LIPASE	CEL

Cell communication

AFFY ID	LOCUSLINK ENTREZ_ID	GENENAME	GENE_SYMBOL
101578_F_AT; 95705_S_AT	11461	ACTIN, BETA, CYTOPLASMIC	ACTB
162196_F_AT	16433	CUB AND ZONA PELLUCIDA-LIKE DOMAINS 1	CUZD1
99479_AT	12945	DELETED IN MALIGNANT BRAIN TUMORS 1	DMBT1
160083_AT; 95611_AT	16956	LIPOPROTEIN LIPASE	LPL
160213_AT; 162312_F_AT	19692	REGENERATING ISLET-DERIVED 1	REG1
95786_AT	19693	REGENERATING ISLET-DERIVED 2	REG2
96047_AT	19662	RETINOL BINDING PROTEIN 4, PLASMA	RBP4
103887_AT	20202	S100 CALCIUM BINDING PROTEIN A9 (CALGRANULIN B)	S100A9

96749_F_AT	238257	TRANSMEMBRANE PROTEIN 30B	TMEM30B

Digestion

AFFY ID	LOCUSLINK ENTREZ_ID	GENENAME	GENE_SYMBOL
100103_F_AT; 100104_R_AT	67373	RIKEN CDNA 2210010C04 GENE	2210010C04RIK
160070_AT	18947	PANCREATIC LIPASE-RELATED PROTEIN 2	PNLIPRP2
160132_AT	109791	RIKEN CDNA 2200003J09 GENE	CLPS
160421_R_AT	66473	CHYMOTRYPSINOGEN B1	CTRB1
160744_R_AT	109660	RIKEN CDNA 1810004D15 GENE	CTRL
92712_AT	73626	RIKEN CDNA 1810009J06 GENE	1810009J06RIK
92873_F_AT	22072	PROTEASE, SERINE, 2	PRSS2
99939_AT	12613	CARBOXYL ESTER LIPASE	CEL

Cellular homeostasis

AFFY ID	LOCUSLINK ENTREZ_ID	GENENAME	GENE_SYMBOL
92768_S_AT	11656	AMINOLEVULINIC ACID SYNTHASE 2, ERYTHROID	ALAS2
98324_AT	15377	FORKHEAD BOX A3	FOXA3
93573_AT	17748	METALLOTHIONEIN 1	MT1
101561_AT	17750	METALLOTHIONEIN 2	MT2
102197_AT	53322	NUCLEOBINDIN 2	NUCB2
101985_AT	18815	PLASMINOGEN	PLG
96588_AT	19218	PROSTAGLANDIN E RECEPTOR 3 (SUBTYPE EP3)	PTGER3

IMMUNE-RELATED PROCESS

Response to stress

AFFY ID	LOCUSLINK ENTREZ_ID	GENENAME	GENE_SYMBOL
94777_AT	11657	ALBUMIN 1	ALB
99862_AT	11625	ALPHA-2-HS-GLYCOPROTEIN	AHSG
103389_AT	30956	AMINOADIPATE-SEMIALDEHYDE SYNTHASE	AASS
92768_S_AT	11656	AMINOLEVULINIC ACID SYNTHASE 2, ERYTHROID	ALAS2
94392_F_AT	11727	ANGIOGENIN, RIBONUCLEASE A FAMILY, MEMBER 1	ANG
162276_I_AT	12260	COMPLEMENT COMPONENT 1, Q SUBCOMPONENT, BETA POLYPEPTIDE	C1QB
99671_AT	11537	COMPLEMENT FACTOR D (ADIPSIN)	CFD
101553_AT	14161	FIBRINOGEN, ALPHA POLYPEPTIDE	FGA
98324_AT	15377	FORKHEAD BOX A3	FOXA3
93875_AT	193740; 15511	HEAT SHOCK PROTEIN 1B	HSPA1B
100150_F_AT	16334	INSULIN II	INS2
161890_F_AT; 96009_S_AT	18489	PANCREATITIS-ASSOCIATED PROTEIN	PAP
160120_I_AT	18778	PHOSPHOLIPASE A2, GROUP IB, PANCREAS	PLA2G1B
101985_AT	18815	PLASMINOGEN	PLG
96588_AT	19218	PROSTAGLANDIN E RECEPTOR 3 (SUBTYPE EP3)	PTGER3
103954_AT; 161642_F_AT	19694	REGENERATING ISLET-DERIVED 3 ALPHA	REG3A
96064_AT	19695	REGENERATING ISLET-DERIVED 3 GAMMA	REG3G
97519_AT	20750	SECRETED PHOSPHOPROTEIN 1	SPP1

MHC-I-mediated Immunity

AFFY ID	LOCUSLINK ENTREZ_ID	GENENAME	GENE_SYMBOL
101578_F_AT; 95705_S_AT	11461	ACTIN, BETA, CYTOPLASMIC	ACTB
97523_I_AT	11723	AMYLASE 2, PANCREATIC	AMY2
162457_F_AT; 94781_AT	15122	HEMOGLOBIN ALPHA, ADULT CHAIN 1	HBA-A1
101869_S_AT; 103534_AT	15127; 15129; 15130	HEMOGLOBIN BETA CHAIN COMPLEX	HBB-B2
98467_AT	16427	INTER ALPHA-TRYPSIN INHIBITOR, HEAVY CHAIN 4	ITIH4
104519_AT	16425	INTER-ALPHA TRYPSIN INHIBITOR, HEAVY CHAIN 2	ITIH2
94716_F_AT; 95775_F_AT	16623; 18048; 18050	KALLIKREIN 1-RELATED PEPTIDASE B1	KLK1B1; KLK1B4; KLK1B9
100719_F_AT	16615	KALLIKREIN 1-RELATED PEPTIDASE B16	KLK1B16
101082_AT	17436	MALIC ENZYME, SUPERNATANT	ME1
96153_AT	18054	NEUTROPHILIC GRANULE PROTEIN	NGP
160070_AT	18947	PANCREATIC LIPASE-RELATED PROTEIN 2	PNLIPRP2
96038_AT	58809	RIBONUCLEASE, RNASE A FAMILY 4	RNASE4
98041_AT	19752	RIBONUCLEASE, RNASE A FAMILY, 1 (PANCREATIC)	RNASE1
94247_AT	109620	RIKEN CDNA 5730453H04 GENE	DSP
97523_I_AT	545562	SIMILAR TO PANCREATIC ALPHA-AMYLASE PRECURSOR (PA) (1,4-ALPHA-D-GLUCAN GLUCANOHYDROLASE)	OTTMUSG00000022462
103562_F_AT	433762	SIMILAR TO RETROVIRUS-RELATED POL POLYPROTEIN (ENDONUCLEASE)	LOC433762
92202_G_AT	235320	ZINC FINGER AND BTB DOMAIN CONTAINING 16	ZBTB16

Immunity & Defense

AFFY ID	LOCUSLINK ENTREZ_ID	GENENAME	GENE_SYMBOL
101058_AT	11722	AMYLASE 1, SALIVARY	AMY1
94392_F_AT	11727	ANGIOGENIN, RIBONUCLEASE A FAMILY, MEMBER 1	ANG
101539_F_AT	104158	CARBOXYLESTERASE 3	CES3
162276_I_AT	12260	COMPLEMENT COMPONENT 1, Q SUBCOMPONENT, BETA POLYPEPTIDE	C1QB
102774_AT	13645	EPIDERMAL GROWTH FACTOR	EGF
101553_AT	14161	FIBRINOGEN, ALPHA POLYPEPTIDE	FGA
160350_AT	14874	GLUTATHIONE TRANSFERASE ZETA 1 (MALEYLACETOACETATE ISOMERASE)	GSTZ1
102918_AT	17829	MUCIN 1, TRANSMEMBRANE	MUC1
96153_AT	18054	NEUTROPHILIC GRANULE PROTEIN	NGP
161890_F_AT; 96009_S_AT	18489	PANCREATITIS-ASSOCIATED PROTEIN	PAP
103954_AT; 161642_F_AT	19694	REGENERATING ISLET-DERIVED 3 ALPHA	REG3A
96064_AT	19695	REGENERATING ISLET-DERIVED 3 GAMMA	REG3G
160145_AT	69036	RIKEN CDNA 1810010M01 GENE	1810010M01RIK
103448_AT	20201	S100 CALCIUM BINDING PROTEIN A8 (CALGRANULIN A)	S100A8

103887_AT	20202	S100 CALCIUM BINDING PROTEIN A9 (CALGRANULIN B)	S100A9
98549_AT	22370	VITRONECTIN	VTN

MHC-II-mediated immunity

AFFY ID	LOCUSLINK ENTREZ_ID	GENENAME	GENE_SYMBOL
94777_AT	11657	ALBUMIN 1	ALB
92768_S_AT	11656	AMINOLEVULINIC ACID SYNTHASE 2, ERYTHROID	ALAS2
101058_AT	11722	AMYLASE 1, SALIVARY	AMY1
101638_S_AT	13114; 13112	CYTOCHROME P450, FAMILY 3, SUBFAMILY A, POLYPEPTIDE 16	CYP3A16; CYP3A11
102774_AT	13645	EPIDERMAL GROWTH FACTOR	EGF
98423_AT	14619	GAP JUNCTION MEMBRANE CHANNEL PROTEIN BETA 2	GJB2
162457_F_AT; 94781_AT	15122	HEMOGLOBIN ALPHA, ADULT CHAIN 1	HBA-A1
101869_S_AT	15127	HEMOGLOBIN BETA CHAIN COMPLEX	HBB-B2
98467_AT	16427	INTER ALPHA-TRYPSIN INHIBITOR, HEAVY CHAIN 4	ITIH4
94716_F_AT; 94773_AT	16623; 18048; 18050	KALLIKREIN 1-RELATED PEPTIDASE B1	KLK1B1; KLK1B4
94716_F_AT	13648; 16612	KALLIKREIN 1-RELATED PEPTIDASE B9	KLK1B9; KLK1
160083_AT; 95611_AT	16956	LIPOPROTEIN LIPASE	LPL
93573_AT	17748	METALLOTHIONEIN 1	MT1
94716_F_AT	76999	RIKEN CDNA 1700127D06 GENE	1700127D06RIK
160744_R_AT	109660	RIKEN CDNA 1810004D15 GENE	CTRL
160306_AT	21835	THYROID HORMONE RESPONSIVE SPOT14 HOMOLOG (RATTUS)	THRSP

Response to external stimulus

AFFY ID	LOCUSLINK ENTREZ_ID	GENENAME	GENE_SYMBOL
101553_AT	14161	FIBRINOGEN, ALPHA POLYPEPTIDE	FGA
101985_AT	18815	PLASMINOGEN	PLG
103448_AT	20201	S100 CALCIUM BINDING PROTEIN A8 (CALGRANULIN A)	S100A8
103887_AT	20202	S100 CALCIUM BINDING PROTEIN A9 (CALGRANULIN B)	S100A9
103954_AT; 161642_F_AT	19694	REGENERATING ISLET-DERIVED 3 ALPHA	REG3A
161890_F_AT; 96009_S_AT	18489	PANCREATITIS-ASSOCIATED PROTEIN	PAP
162276_I_AT	12260	COMPLEMENT COMPONENT 1, Q SUBCOMPONENT, BETA POLYPEPTIDE	C1QB
94777_AT	11657	ALBUMIN 1	ALB
96064_AT	19695	REGENERATING ISLET-DERIVED 3 GAMMA	REG3G
96588_AT	19218	PROSTAGLANDIN E RECEPTOR 3 (SUBTYPE EP3)	PTGER3
97519_AT	20750	SECRETED PHOSPHOPROTEIN 1	SPP1
98324_AT	15377	FORKHEAD BOX A3	FOXA3
99671_AT	11537	COMPLEMENT FACTOR D (ADIPSIN)	CFD
99862_AT	11625	ALPHA-2-HS-GLYCOPROTEIN	AHSG

Response to wounding

AFFY ID	LOCUSLINK ENTREZ_ID	GENENAME	GENE_SYMBOL

99862_AT	11625	ALPHA-2-HS-GLYCOPROTEIN	AHSG
162276_I_AT	12260	COMPLEMENT COMPONENT 1, Q SUBCOMPONENT, BETA POLYPEPTIDE	C1QB
99671_AT	11537	COMPLEMENT FACTOR D (ADIPSIN)	CFD
101553_AT	14161	FIBRINOGEN, ALPHA POLYPEPTIDE	FGA
161890_F_AT; 96009_S_AT	18489	PANCREATITIS-ASSOCIATED PROTEIN	PAP
101985_AT	18815	PLASMINOGEN	PLG
96588_AT	19218	PROSTAGLANDIN E RECEPTOR 3 (SUBTYPE EP3)	PTGER3
103954_AT; 161642_F_AT	19694	REGENERATING ISLET-DERIVED 3 ALPHA	REG3A
96064_AT	19695	REGENERATING ISLET-DERIVED 3 GAMMA	REG3G
97519_AT	20750	SECRETED PHOSPHOPROTEIN 1	SPP1

Inflammatory response

AFFY ID	LOCUSLINK ENTREZ_ID	GENENAME	GENE_SYMBOL
103954_AT; 161642_F_AT	19694	REGENERATING ISLET-DERIVED 3 ALPHA	REG3A
161890_F_AT; 96009_S_AT	18489	PANCREATITIS-ASSOCIATED PROTEIN	PAP
162276_I_AT	12260	COMPLEMENT COMPONENT 1, Q SUBCOMPONENT, BETA POLYPEPTIDE	C1QB
96064_AT	19695	REGENERATING ISLET-DERIVED 3 GAMMA	REG3G
96588_AT	19218	PROSTAGLANDIN E RECEPTOR 3 (SUBTYPE EP3)	PTGER3
97519_AT	20750	SECRETED PHOSPHOPROTEIN 1	SPP1
99671_AT	11537	COMPLEMENT FACTOR D (ADIPSIN)	CFD
99862_AT	11625	ALPHA-2-HS-GLYCOPROTEIN	AHSG

Cytokine & chemokine mediated signaling pathway

AFFY ID	LOCUSLINK ENTREZ_ID	GENENAME	GENE_SYMBOL
103257_AT	99887	RIKEN CDNA 4930577M16 GENE	TMEM56
160306_AT	21835	THYROID HORMONE RESPONSIVE SPOT14 HOMOLOG (RATTUS)	THRSP
160481_AT	18534	PHOSPHOENOLPYRUVATE CARBOXYKINASE 1, CYTOSOLIC	PCK1
161046_AT	12931	CYTOKINE RECEPTOR-LIKE FACTOR 1	CRLF1
95786_AT	19693	REGENERATING ISLET-DERIVED 2	REG2
98467_AT	16427	INTER ALPHA-TRYPSIN INHIBITOR, HEAVY CHAIN 4	ITIH4
99862_AT	11625	ALPHA-2-HS-GLYCOPROTEIN	AHSG

Other Immune defense

AFFY ID	LOCUSLINK ENTREZ_ID	GENENAME	GENE_SYMBOL
100086_AT	16976	LOW DENSITY LIPOPROTEIN RECEPTOR-RELATED PROTEIN ASSOCIATED PROTEIN 1	LRPAP1
102169_AT	258051	OLFACTORY RECEPTOR 93	OLFR93
102918_AT	17829	MUCIN 1, TRANSMEMBRANE	MUC1
160083_AT; 95611_AT	16956; 16956	LIPOPROTEIN LIPASE	LPL
161677_R_AT	80294	RIKEN CDNA 2310011G23 GENE	POFUT2
96153_AT	18054	NEUTROPHILIC GRANULE PROTEIN	NGP
98423_AT	14619	GAP JUNCTION MEMBRANE CHANNEL PROTEIN BETA 2	GJB2

Down-regulated genes

Metabolic Process

Antigen binding

AFFY ID	LOCUSLINK ENTREZ_ID	GENENAME	GENE_SYMBOL
102721_AT	380795	EXPRESSED SEQUENCE AI324046 IMMUNOGLOBULIN HEAVY CHAIN COMPLEX	IgG3
102824_G_AT	380794	EXPRESSED SEQUENCE AU044919	IGHG2A
103422_AT	12479	CD1D1 ANTIGEN	CD1D1
93086_AT	16071	IMMUNOGLOBULIN KAPPA CHAIN, CONSTANT REGION	IGK-C
93086_AT	243469	IMMUNOGLOBULIN KAPPA LIGHT CHAIN COMPLEX	IGK
93086_AT	16114	IMMUNOGLOBULIN KAPPA CHAIN VARIABLE 28 (V28)	IgK-V28
93638_S_AT	16142	IMMUNOGLOBULIN LAMBDA CHAIN, VARIABLE 1	IGL-V1

Metabolic process

AFFY ID	LOCUSLINK ENTREZ_ID	GENENAME	GENE_SYMBOL
101054_AT	16149	CD74 ANTIGEN (INVARIANT POLYPEPTIDE OF MAJOR HISTOCOMPATIBILITY COMPLEX, CLASS II ANTIGEN-ASSOCIATED)	CD74
101065_AT	18538	PROLIFERATING CELL NUCLEAR ANTIGEN	PCNA
101137_AT	27050	RIBOSOMAL PROTEIN S3	RPS3
102381_AT	50790	ACYL-COA SYNTHETASE LONG-CHAIN FAMILY MEMBER 4	ACSL4
103422_AT	12479	CD1D1 ANTIGEN	CD1D1
103454_AT	20728	SPI-C TRANSCRIPTION FACTOR (SPI-1/PU.1 RELATED)	SPIC
103617_AT	13136	DECAY ACCELERATING FACTOR 1	CD55
160199_AT	15381	HETEROGENEOUS NUCLEAR RIBONUCLEOPROTEIN C	HNRNPC
160234_AT	230484	UBIQUITIN SPECIFIC PEPTIDASE 1	USP1
161360_AT	18245	ORNITHINE DECARBOXYLASE ANTIZYME	OAZ1
92660_F_AT	22194	UBIQUITIN-CONJUGATING ENZYME E2E 1, UBC4/5 HOMOLOG (YEAST)	UBE2E1
92992_I_AT	20688	TRANS-ACTING TRANSCRIPTION FACTOR 4	SP4
94766_AT	13627	EUKARYOTIC TRANSLATION ELONGATION FACTOR 1 ALPHA 1	EEF1A1
96699_AT	15312	HIGH MOBILITY GROUP NUCLEOSOMAL BINDING DOMAIN 1	HMGN1
98968_AT	17918	MYOSIN VA	MYO5A
99462_AT	21974	TOPOISOMERASE (DNA) II BETA	TOP2B
99574_AT	387524	ZINC AND RING FINGER 2	ZNRF2

Biological Process

Immunity and Defense

AFFY ID	LOCUSLINK ENTREZ_ID	GENENAME	GENE_SYMBOL
101054_AT	16149	CD74 ANTIGEN (INVARIANT POLYPEPTIDE OF MAJOR HISTOCOMPATIBILITY COMPLEX, CLASS II ANTIGEN-ASSOCIATED)	CD74

102721_AT	380795	EXPRESSED SEQUENCE AI324046	AI324046
102824_G_AT	380794	EXPRESSED SEQUENCE AU044919	IGHG
103422_AT	12479	CD1D1 ANTIGEN	CD1D1
103518_AT	13025	CYTOTOXIC T LYMPHOCYTE-ASSOCIATED PROTEIN 2 BETA	CTLA2B
103617_AT	13136	DECAY ACCELERATING FACTOR 1	CD55
93086_AT	16071	IMMUNOGLOBULIN KAPPA CHAIN, CONSTANT REGION	IGK-C
93086_AT	243469	IMMUNOGLOBULIN KAPPA CHAIN COMPLEX	IGK
93086_AT	434039; 16114	IMMUNOGLOBULIN KAPPA CHAIN VARIABLE 28 (V28)	IgK-V28
93638_S_AT	623736	IMMUNOGLOBULIN LAMBDA CHAIN, VARIABLE 1	IGL-V1
95634_AT	104457	RIKEN CDNA 0610010K14 GENE	0610010K14RIK
96971_F_AT	545849	IMMUNOGLOBULIN LIGHT CHAIN VARIABLE REGION	IGKV4-78
99462_AT	21974	TOPOISOMERASE (DNA) II BETA	TOP2B

B-cell and Antibody mediated immunity

AFFY ID	LOCUSLINK ENTREZ_ID	GENENAME	GENE_SYMBOL
100048_AT	109905	RAS-RELATED PROTEIN-1A	RAP1A
102721_AT	380795	EXPRESSED SEQUENCE AI324046	AI324046
102824_G_AT	380794	EXPRESSED SEQUENCE AU044919	IGHG
93086_AT	545854	SIMILAR TO IG KAPPA CHAIN C REGION	IGK-C
93086_AT	16071	IMMUNOGLOBULIN KAPPA CHAIN, CONSTANT REGION	IGK
93086_AT	434039	IMMUNOGLOBULIN KAPPA CHAIN VARIABLE 28 (V28)	IGL-V1
96971_F_AT	545849	IMMUNOGLOBULIN LIGHT CHAIN VARIABLE REGION	IGKV4-78

IMMUNE RELATED PROCESS

Response to stress

AFFY ID	LOCUSLINK ENTREZ_ID	GENENAME	GENE_SYMBOL
101054_AT	16149	CD74 ANTIGEN (INVARIANT POLYPEPTIDE OF MAJOR HISTOCOMPATIBILITY COMPLEX, CLASS II ANTIGEN-ASSOCIATED)	CD74
101065_AT	18538	PROLIFERATING CELL NUCLEAR ANTIGEN	PCNA
103422_AT	12479	CD1D1 ANTIGEN	CD1D1
103617_AT	13136	DECAY ACCELERATING FACTOR 1	CD55
93638_S_AT	16142	IMMUNOGLOBULIN LAMBDA CHAIN, VARIABLE 1	IGL-V1
96699_AT	15312	HIGH MOBILITY GROUP NUCLEOSOMAL BINDING DOMAIN 1	HMGN1

Humoral Immune Response

AFFY ID	LOCUSLINK ENTREZ_ID	GENENAME	GENE_SYMBOL
101054_AT	16149	CD74 ANTIGEN (INVARIANT POLYPEPTIDE OF MAJOR HISTOCOMPATIBILITY COMPLEX, CLASS II ANTIGEN-ASSOCIATED)	CD74
103422_AT	12479	CD1D1 ANTIGEN	CD1D1
103617_AT	13136	DECAY ACCELERATING FACTOR 1	CD55
93638_S_AT	16142	IMMUNOGLOBULIN LAMBDA CHAIN, VARIABLE 1	IGL-V1

Antigen binding

AFFY ID	LOCUSLINK ENTREZ_ID	GENENAME	GENE_SYMBOL
102721_AT	380795	EXPRESSED SEQUENCE AI324046	AI324046
102824_G_AT	380794	EXPRESSED SEQUENCE AU044919	IGHG
103422_AT	12479	CD1D1 ANTIGEN	CD1D1
93086_AT	16071	IMMUNOGLOBULIN KAPPA CHAIN, CONSTANT REGION	IGK-C
93086_AT	243469	IMMUNOGLOBULIN KAPPA CHAIN COMPLEX	IGK
93086_AT	434039	IMMUNOGLOBULIN KAPPA CHAIN VARIABLE 28 (V28)	ENSMUSG00000076576
93638_S_AT	16142	IMMUNOGLOBULIN LAMBDA CHAIN, VARIABLE 1	IGL-V1

Lymphocyte mediated immunity

AFFY ID	LOCUSLINK ENTREZ_ID	GENENAME	GENE_SYMBOL
101054_AT	16149	CD74 ANTIGEN (INVARIANT POLYPEPTIDE OF MAJOR HISTOCOMPATIBILITY COMPLEX, CLASS II ANTIGEN-ASSOCIATED)	CD74
103422_AT	12479	CD1D1 ANTIGEN	CD1D1
103617_AT	13136	DECAY ACCELERATING FACTOR 1	CD55

Developmental process

AFFY ID	LOCUSLINK ENTREZ_ID	GENENAME	GENE_SYMBOL
100048_AT	109905	RAS-RELATED PROTEIN-1A	RAP1A
101137_AT	27050	RIBOSOMAL PROTEIN S3	RPS3
102818_AT	546282	SIMILAR TO XMR	EG546282
102818_AT	22526	XLR-RELATED, MEIOSIS REGULATED	ENSMUSG00000073257
102818_AT	546272	SIMILAR TO XLR-RELATED, MEIOSIS REGULATED	EG546272
102818_AT	382277	XMR PROTEIN	XMR
102824_G_AT	380794	EXPRESSED SEQUENCE AU044919	IGHG
92660_F_AT	22194	UBIQUITIN-CONJUGATING ENZYME E2E 1, UBC4/5 HOMOLOG (YEAST)	UBE2E1
94766_AT	13627	EUKARYOTIC TRANSLATION ELONGATION FACTOR 1 ALPHA 1	EEF1A1
99149_AT	66949	TRIPARTITE MOTIF-CONTAINING 59	TRIM59
