# Supplementary material for: Early over expression of messenger RNA for multiple genes, including insulin, in the Pancreatic Lymph Nodes of NOD mice is associated with Islet Autoimmunity
Source: BMC Med Genomics. 2009 Oct 2;2:63. doi: 10.1186/1755-8794-2-63 (PMC2763872; doi:10.1186/1755-8794-2-63)
Supplement: Additional file 6 — Assignment of functional categories as annotated by literature data mining, for genes and ESTs differentially expressed in the PLN of E-IAA NOD sub-phenotype, as described in the text (Table 3). References, corresponding to bibliographic search for functions for each gene, are given. [file 1755-8794-2-63-S6.PDF]

Functional categories for genes and ESTs differentially expressed in the PLN of E-IAA NOD sub phenotype. Each gene is represented once in each functional category. When more than one Affymetrix probe sets, corresponding to the same gene, have been hybridized with the target, all the corresponding probe sets have been grouped. Gene functional category assignment has been realized by manual data mining as described in the text for genes on Table 3. References corresponding to bibliographic search for functions for each gene are shown.

Inflammation & Infection

Putative Function	Gene name	Fold change	GO biological process	GO molecular function	GO cellular component	Affymetrix ID	Public ID	Gene symbol [Reference]
Inflammation, regeneration	Regenerating islet-derived 2	58,44	---	sugar binding	extracellular	95786_at	D14011	Reg2 [1]
Inflammation, regeneration	Regenerating islet-derived 1	57,02	---	sugar binding	extracellular	160213_at; 162312_f_at	D14010	Reg1 [1, 2]
Inflammation, autoimmunity	Trypsin 4	52,9	proteolysis	serine-type	---	101043_f_at	AE000664	Try4 [3]
Inflammation, regeneration	Pancreatitis-associated protein	45,63	acute-phase/ inflammatory response	sugar binding	extracellular	161890_f_at; 96009_s_at	AV371861	Pap [4]; [5]
Infection *H. Pylori*	Trefoil factor 2 (spasmolytic protein 1)	39,97	---	---	extracellular	93302_at	U78770	Tff2 [6]; [7]; [8]; [9]
Inflammation, autoimmunity	Kallikrein 1	37,92	proteolysis	serine-type/growth factor activity	extracellular	100061_f_at	M13500	Klk1b5 [10]; [11, 12]
Inflammation, autoimmunity	Trypsin 10	28,39	proteolysis	serine-type	---	101338_f_at	AE000664	Try10 [13]
Inflammation, regeneration	Regenerating islet-derived 3 alpha	28,09	acute-phase/ inflammatory response	sugar binding	extracellular	103954_at; 161642_f_at	D63357	Reg3a [14], [2]
Inflammation, autoimmunity	Kallikrein 1-related peptidase b22	21,55	proteolysis	serine-type	extracellular	101289_f_at	M17979	Klk1b22 [10-12]
Inflammation, autoimmunity	Kallikrein 1-related peptidase b9	19,71	proteolysis	serine-type	extracellular	94716_f_at	M17962	Klk1b9 [10-12]
Inflammation, autoimmunity	Kallikrein 1-related peptidase b5	17,34	proteolysis	serine-type	extracellular	104495_f_at	Y00500	Klk1b5 related; [10]; [10-12]
Inflammation, autoimmunity	Similar to Kallikrein 1-related peptidase b9 (KlK1rb9)	14,27	---	---	---	100681_f_at	V00829	mGK2 fragment similar [15]
Inflammation, autoimmunity	Kallikrein 1-related peptidase b1	13,04	proteolysis	serine-type	extracellular	95775_f_at	V00829	Klk1b1 [16]
Inflammation, pancreatitis	CUB and zona pellucida-like domains1	11,73	cell cycle & division	protein binding	extracellular	162196_f_at	AV059956	Cuzd1 [17],
Response to stress	Phospholipase A2, group IB, pancreas	9,69	response to stress/ cell proliferation	phospholipase A2activity /calcium ion binding	extracellular	160120_i_at	AI327450	Pla2g1b [18], [19]; [20]
Inflammation	Metallothionein 1	6,81	nitric oxide mediated signal transduction	metal ion binding	lysosome	93573_at	V00835	Mt1 [21]

Inflammation, transcription factor, stress induced	Nuclear protein 1 (p8 family)	6,49	cell growth	---	nucleus	160108_at	AI852641	Nupr1 [22-25],[26]
Inflammation	Metallothionein 2	6,11	nitric oxide mediated signal transduction	metal ion binding	---	101561_at	K02236	Mt2 [27]
Inflammation, cancer autoimmunity, infection	Secreted phosphoprotein 1	5,68	anti-apoptosis /inflammatory response /T-helper 1 type immune response	cytokine activity	extracellular	97519_at	X13986	Spp1, [28]; [29], [30]; [31]; [32]; [33]
Inflammation, autoimmunity	Kallikrein 1-related peptidase b16	3,44	proteolysis	serine-type/growth factor activity	extracellular	100719_f_at	J03877	Klk1b16 [11]
Inflammation, cancer	Keratin 18	3,15	cytoskeleton organization and biogenesis	structural constituent of cytoskeleton	mitochondrion	94270_at	M22832	Krt18 [34]
Inflammation, cancer, infection	Alpha-2-HS-glycoprotein	3	acute-phase response/regulation of inflammatory response	cysteine protease inhibitor activity	extracellular	99862_at	AF025821	Ahsg [35]; [36]; [37]
Inflammation, cancer	Keratin 8	2,15	cellular morphogenesis/ cytoskeleton organization and biogenesis/response to other organism	protein kinase activity	intermediate filament	101009_at	X15662	Krt8 [34]; [38]
Inflammation	Vitronectin	2,13	cell adhesion	protein binding	extracellular	98549_at	M77123	Vtn [39]; for opn & vtn see [40]
Inflammation, autoimmunity	Kallikrein 1	2,1	proteolysis positive regulation of phosphoinositide 3-kinase activity	serine-type/growth factor activity	extracellular	100060_i_at	M13500	Klk1 [41], [42]
Inflammation, wound, cancer, hemostasis	Plasminogen	2,08	apoptosis/induction of apoptosis/tissue development /negative regulation of angiogenesis/tissue regeneration	serine-type	extracellular	101985_at	J04766	Plg [43]
Inflammation, autoimmunity	Kallikrein 1-related petidase b26	1,98	proteolysis	serine-type	extracellular	102693_f_at	J00389	Klk1b26 [41], [42]
Inflammation, cancer, metastasis	S100 calcium binding protein A9 (calgranulin B)	1,81	leukocyte chemotaxis/actin cytoskeleton reorganization	calcium ion binding	---	103887_at	M83219	S100a9 [44]
Inflammation, regeneration	Regenerating islet-derived 3 gamma	1,77	acute-phase response/inflammatory response	sugar binding	extracellular	96064_at	D63362	Reg3g [1], [2]
Inflammation, autoimminity	Heat shock protein 1A	1,75	telomere maintenance/DNA repair/protein folding/response to heat	nucleotide binding	---	93875_at	M12571	Hspa1a [45]; [46]
Inflammation	Resistin	1,73	---	hormone activity	extracellular	102366_at	AA718169	Retn [47], [48]; [49]; [50]

Inflammation, cancer, metastasis	S100 calcium binding protein A8 (calgranulin A)	1,49	chemotaxis	calcium ion binding	---	103448_at	M83218	S100a8 [44]
Inflammation, cancer, autoim, wound, infect	Solute carrier family 23 (nucleobase transporters), member 3	1,48	transport	transporter activity	membrane	161149_r_at	AV222871	Slc23a3 [51]
Infection, Endocytosis	Growth hormone receptor	1,44	endocytosis	receptor activity	nucleus	99108_s_at	U15012	Ghr [52]

Immune Function

Putative Function	Gene title	Fold change	GO biological process term	GO molecular function term	GO cellular component term	Affymetrix ID	Representa tive Public ID	Gene symbol [Reference]
Immune function	Ribonuclease, RNase A family, 1 (pancreatic)	93,56	---	nucleic acid binding	extracellular	98041_at	X60103	Rnase1 [53]
Infection	Syncollin	57,36	exocytosis	---	extracellular	95509_at	AA607809	Sycn [54]; [55]
Inflammation, regeneration, malignacy	Deleted in malignant brain tumors 1	41,28	positive regulation of epithelial cell differentiation	scavenger receptor activity	extracellular matrix	99479_at	U37438	Dmbt1 [56]
Expressed in macrophages & Leukocytes	Elastase 1, pancreatic	34,91	proteolysis	serine-type	---	93783_at	M27347	Ela1 [3]; [57], 2005 ; [58]; [59]
Inflammation	Elastase 2A	11,8	proteolysis	serine-type	extracellular	94037_at	X04573	Ela2A [60]
Autoimmunity	Epidermal growth factor binding protein type B	10,26	proteolysis	serine-type	extracellular	161637_f_at	AV053185	Egfbp2 [15]; [12]; [61]; [16]; with opn see [33]; [12]
Vascularization, Viral infection	Angiogenin, ribonuclease, RNase A family, 5	3,42	angiogenesis/response to hypoxia /proliferation/cell differentiation/activation of phospholipase A2 /positive regulation of protein secretion	nucleic acid binding /actin binding	extracellular	94392_f_at	U22516	Ang [62]
Metastasis, hemostasis	Fibrinogen, alpha polypeptide	2,66	signal transduction/platelet activation	receptor binding	extracellular	101553_at	AI876446	Fga [63]; [64]; [43]
Immune function	Cytokine receptor-like factor 1	2,45	---	receptor activity / cytokine binding	extracellular	161046_at	AA270365	Crlf1 [65]
Immune response, innate	Complement component 1, q subcomponent, beta polypeptide	2,23	Innate immune response/complement activation	---	extracellular	162276_i_at	AV367855	C1qb [66]
Immune function, vacscularisation	Ribonuclease, RNase A family 4	2,12	angiogenesis/response to hypoxia /activation of phospholipase A2/ positive regulation of protein secretion	nucleic acid binding	extracellular	96038_at	AI840339	Rnase4 [67]
Innate immune response	Complement factor D (adipsin)	2	proteolysis/complement activation, alternative pathway/innate immune response	complement factor D activity/serine-type	extracellular	99671_at	X04673	Cfd [68], [69, 70], [71]

TGFβ Signaling_ Pathway	Epidermal growth factor	1,94	activation of MAPKK activity / positive regulation of cell proliferation	calcium ion binding /growth factor activity	extracellular	102774_at	V00741	Egf [72]
Defence response, homeostasis	Neutrophilic granule protein	1,5	defense response	cysteine protease inhibitor activity	extracellular	96153_at	L37297	Ngp [73]
Humoral immune response	Immunoglobulin kappa chain variable 28 (V28)	-0,59	---	---	---	96971_f_at	X00652	Similar to Ig
Humoral immune response, antigen binding	Immunoglobulin kappa chain, constant region	-1,36	humoral immune response	antigen binding	---	93086_at	M18237	Cr1
Immune function protein binding cytokine binding MHC class II protein binding	CD74 antigen (invariant polypeptide of major histocompatibility complex, class II antigen-associated)	-1,42	immune response /immunoglobulin mediated /antigen processing &presentation /regulation of T cell differentiation	cytokine binding /MHC class II protein binding	lysosome / membrane	101054_at	X00496	Cd74 [74]
Immune function	Myosin Va	-1,49	cytoskeleton organization and biogenesis/insulin secretion /regulation of IP3 receptor activity/ synapse organization and biogenesis	nucleotide binding/actin binding	cytoplasm	98968_at	X57377	Myo5a [75]; [76]; [77], [78]; [79]
Autoimmunity, cancer, infection	CD1d1 antigen	-1,53	antigen processing &presentation/positive thymic T cell selection & NK T cell activation	protein binding/endogenous lipid antigen binding	lysosome	103422_at	M63695	Cd1d1 [80], [81], [81], [82]
Immune system T cells	Cytotoxic T lymphocyte-associated protein 2 beta	-1,56	---	cysteine protease inhibitor activity/protein binding	---	103518_at	X15592	Ctla2b [83]
Immune response	CD55 antigen	-1,65	innate immune response/complement activation, classical pathway	GPI anchor binding	extracellular	103617_at	D63679	Cd55 [84],
Immune function	Cytokine receptor-like factor 3	-1,69	---	---	---	98415_at	AF046060	Crlf3 [85]
Immune related, Signal transduction	Membrane-spanning 4-domains, subfamily A, member 6B	-1,78	signal transduction	receptor activity	membrane	97322_at	AI835093	Ms4a6b [86]
Role in the regulation of integrins	RAS-related protein-1a	-1,81	cell cycle	nucleotide binding	intracellular /// membrane	100048_at	AW049685	Rap1a [87]
Humoral immune response	Expressed sequence AI324046	-1,85	---	---	---	102721_at	D14625	IgG1c [88]
Humoral immune response	Immunoglobulin lambda chain, variable 1	-1,96	humoral immune response	antigen binding	---	93638_s_at	J00579	Igl-V1 [88]
Humoral immune response	Immunoglobulin heavy chain (gamma polypeptide)	-2,17	humoral immune response/immunoglobulin mediated immune response/phagocytosis	antigen binding	membrane	102824_g_at	X67210	Ighg [88]

Cancer

Putative Function	Gene title	Fold change	GO biological process term	GO molecular function term	GO cellular component term	Affymetrix ID	Representa tive Public ID	Gene symbol [Reference]
Cancer, infection	Mucin 1, trans membrane	5,23	---	actin binding	extracellular	102918_at	M84683	Muc1 [89];[90]
Cancer	Gamma-glutamyl hydrolase	4,76	glutamine metabolic process	catalytic activity	extracellular	93575_at	AF051102	Ggh [91]
Cancer involved but unknown function	Trans membrane protein 97	3,78	---	---	extracellular	95137_at	AI852985	Tmem97 [92]
Cancer	Cytochrome P450, family 3, subfamily a, polypeptide 11	3,42	electron transport	heme binding / metal ion binding	endoplasmic reticulum	101638_s_at	D26137	Cyp3a11 [93]
Cancer, viral infection, ER, ubiquitination	Sel-1 suppressor of lin-12-like (C. elegans)	2,27	Notch signaling pathway	binding	extracellular	92871_at	AW121840	Sel1l [94], [95],[9]
Cancer, metastasis	Hepsin	2,25	proteolysis	serine-type	plasma membrane	160639_at	AF030065	Hpn [96], [97]
Cancer	Actin, beta, cytoplasmic	2,13	---	structural molecule activity	cytoplasm	101578_f_at; 95705_s_at	M12481	Actb[98]
Cell (intercell) communication, cancer in LN	Gap junction membrane channel protein beta 2	1,96	cell-cell signaling & communication	protein binding	membrane	98423_at	M81445	Gjb2 [99]; [100]
Cancer	Asparagine synthetase	1,84	metabolic process	ligase activity	soluble fraction	95133_at; 161221_f_at	AV253908; U38940	Asns [101]
Cancer, wound	Desmoplakin	1,8	---	---	mitochondrion	94247_at	AA600542	Dsp [102]
Cancer involved unknown function	Trans membrane protein 56	1,76	---	---	membrane	103257_at	AA690483	Tmem56 [103]
Cytoskeleton organization and biogenesis actin, nucleotide binding, cancer	Ras homolog gene family, member U	1,74	G1/S transition of mitotic cell cycle	nucleotide binding	intracellular	96747_at	AW121294	Rhou [104]
Cancer, stress response	similar to N-myc downstream regulated 2	1,62	---	---	---	161610_at	AV349686	Ndrg2, EST [105]; [106]
Cancer	Trans membrane protein 30B	1,38	---	---	membrane	96749_f_at	AA619554	Tmem30b [92]
Cancer	Cytochrome P450, family 2, subfamily e, polypeptide 1	1,33	electron transport	monooxygenase activity	extracellular	93996_at	X01026	Cyp2e1 [93]
Cancer, Colorectal	Ribosomal protein S3	-1,44	translation	nucleic acid binding/structural constituent of ribosome	cytoplasm / ribosome	101137_at	X76772	Rps3 [107]
Cancer, leukemia	Topoisomerase (DNA) II beta	-1,81	DNA metabolic process / development	nucleotide binding	nucleus	99462_at	D38046	Top2b [108]

Enzyme

Putative Function	Gene title	Fold change	GO biological process term	GO molecular function term	GO cellular component term	Affymetrix ID	Representa tive Public ID	Gene symbol [Reference]
TCRβ locus Unknown	Protease, serine, 3	68,26	proteolysis	serine-type	mitochondrion	93208_at	AE000665	Prss3 [13], [109], [110]
TCRβ locus Unknown	Protease, serine, 2	48,73	proteolysis	serine-type	extracellular	92873_f_at	X04574	Prss2 [111]

Proteolysis	Chymotrypsin-like	33,97	proteolysis	serine-type	extracellular	160744_r_at	AA244542	Ctrl [65]; [112]
Proteolysis	Chymotrypsin ogen B1	29,23	proteolysis	serine-type	extracellular	160421_r_at	AA590358	Ctrb1 [112] [3]
Unknown, Highly polymorphic locus	Phenylalanine hydroxylase	9,73	metabolic process	catalytic activity	---	95407_at	X51942	Pah [113]
TCRβ locus, Unknown	Protease, serine, 3	2,43	proteolysis	serine-type	mitochondrion	101339_at	AE000665	Prss3 [114]
Mitochondria, Metabolism	Aldehyde dehydrogenase family 7, member A1	2,42	metabolic process	oxidoreductase activity	mitochondrion	97450_s_at	AA986258	Aldh7a1 [115]
Mitochondria, Metabolism	Glycine C-acetyl transferase (2-amino-3-ketobutyrate-coenzyme A ligase)	2,4	metabolic process	catalytic activity	mitochondrion	160628_at	AI849354	Gcat [116]
Catalytic activity, mitochondria	Malic enzyme, supernatant	2,25	metabolic process/response to hormone stimulus	enzyme activity	cytoplasm	101082_at	J02652	Mod1 [117]
Mitochondrion, catalytic activity, heme binding	Aminolevulinic acid synthase 2, erythroid	2,06	metabolic process	catalytic activity	mitochondrion	92768_s_at	M15268	Alas2 [118]
Lipid metabolic process, Gluconeogenesis	Phosphoenol pyruvate carboxykinase 1, cytosolic	1,93	gluconeogenesis/ lipid metabolic process	nucleotide binding	cytoplasm	160481_at	AF009605	Pck1 [119]
Proteolysis, found in T2D	Carboxy peptidase N, polypeptide 1	1,9	proteolysis	carboxypeptid ase activity metal ion binding	---	103882_at	AI182588	Cpn1 [115]
Catalytic activity, fatty acid degradation	Lipoprotein lipase	1,87	lipid metabolic process	catalytic activity	extracellular	160083_at; 95611_at	M63335	Lpl [120, 121]; [122]
Catalytic activity, metabolic process, response to toxin	Carboxylesterase 3	1,58	response to toxin	catalytic activity	extracellular	101539_f_at	AW226939	Ces3 [123]
Mitochondrion response to oxidative stress	Aminoadipate-semialdehyde synthase	1,52	response to oxidative stress	catalytic activity	mitochondrion	103389_at	AJ224761	Aass [124]
Catalytic activity, mitochondrion	Acyl-CoA synthetase long-chain family member 4	-1,59	lipid metabolic process	magnesium ion binding/cataly-tic activity	mitochondrion	102381_at	AA619207	Acsl4 [125]

Diabetes

Putative Function	Gene title	Fold change	GO biological process term	GO molecular function term	GO cellular component term	Affymetrix ID	Representa tive Public ID	Gene symbol [Reference]
Lipase Diabetes	Pancreatic lipase related protein 1	70,76	lipid metabolic process	catalytic activity	extracellular	92601_at	AA674409	Pnliprp1 [126]
Diabetes related	Amylase 2-1, pancreatic	56,49	metabolic process	catalytic activity	extracellular	97523_i_at; 97524_f_at	X02578	Amy2-1 [127], [128]
Diabetes	Insulin II	45,73	glucose transport	hormone activity	extracellular	100150_f_at	X04724	Ins2

Lipase Diabetes	Carboxyl ester lipase	40,2	lipid catabolic process	carboxylesterase activity	extracellular	99939_at	U37386	Cel [129]; [130]
T2D	Colipase, pancreatic	33,99	digestion/lipid catabolic process	enzyme activator activity	extracellular	160132_at	AA710635	56. Clps [131], [132]
Diabetes	Insulin I	16,28	glucose metabolic process	hormone activity	extracellular	97658_f_at; 97659_r_at	X04725	Ins1
Diabetes related	Amylase 1, salivary	13,63	metabolic process	catalytic activity	extracellular	101058_at	J00356	Amy1
Lipase, Diabetes	Pancreatic lipase-related protein 2	11,15	lipid metabolic process/cellular defense response/digestion	catalytic activity	extracellular	160070_at	M30687	Pnliprp2 [126]; [133, 134]
Apoptosis, vascularization & diabetes	Albumin	7,52	negative regulation of apoptosis	binding/transmembrane transporter activity	extracellular	94777_at	X13060	Alb [113],
Related to T2D, NK cells, protein folding	FK506 binding protein 11	3,11	protein folding	isomerase activity	extracellular	97964_at	AW122851	Fkbp11 [135], [136], [137]
Insulin resistance	Retinol binding protein 4, plasma	2,18	transport	transporter activity	extracellular	96047_at	U63146	Rbp4 [138]

Regeneration & remodeling

Putative Function	Gene title	Fold change	GO biological process term	GO molecular function term	GO cellular component term	Affymetrix ID	Representative Public ID	Gene symbol [Reference]
Remodeling, Regeneration liver	Inter alpha-trypsin inhibitor, heavy chain 4	3,73	hyaluronan metabolic process	serine-type endopeptidase inhibitor activity	cytoplasm	98467_at	AF023919	Itih4 [139]; [13]
Regeneration	Hemoglobin, beta adult minor chain	3,02	transport/oxygen transport	oxygen transporter activity	hemoglobin complex	103534_at; 101869_s_at	V00722	Hbb-b2
Regeneration	Hemoglobin alpha, adult chain 1	2,82	oxygen transport	oxygen transporter activity	mitochondrion	94781_at; 162457_f_at	V00714	70. Hba-a2
Remodeling tissue, tumor invasion	Inter-alpha trypsin inhibitor, heavy chain 2	2,31	hyaluronan metabolic process	serine-type endopeptidase inhibitor activity	extracellular	104519_at	X70392	Itih2 [139], [13],
Wound healing & angiogenesis	Prostaglandin E receptor 3 (subtype EP3)	1,86	signal transduction/G-protein signaling, coupled to IP3 second messenger (phospholipase C activating)/elevation of cytosolic calcium ion concentration	receptor activity	plasma membrane	96588_at	D10204	Ptger3 [140]; [141]
Cell proliferation, myeloid cell differentiation	Zinc finger and BTB domain containing 16	1,42	development/negative regulation of cell proliferation/embryonic pattern specification & morphogenesis/positive regulation of apoptosis/ regulation of transcription	nucleic acid binding/specific transcriptional repressor activity	nucleus	92202_g_at	AI553024	Zbtb16 [142]
Cell proliferation & transformation	Ornithine decarboxylase antizyme	-1,32	polyamine metabolic process	enzyme inhibitor activity	---	161360_at	AV212241	Oaz1 [143]
Cell proliferation	Ecotropic viral integration site 2a	-1,79	cell proliferation	transmembrane receptor activity	membrane	98025_at	M34896	Evi2a [144]

Other

Putative Function	Gene title	Fold change	GO biological process term	GO molecular function term	GO cellular component term	Affymetrix ID	Representative Public ID	Gene symbol [Reference]
Cytoskeleton, Actin binding Immune related	Group specific component	3,07	transport/vitamin D metabolic process	actin binding/vitamin D binding	extracellular	99197_at	M55413	Gc [145], [146]
Homeostasis, calcium	Nucleobindin 2	2,48	cellular calcium ion homeostasis	DNA binding/calcium ion binding	extracellular	102197_at	AJ222586	Nucb2 [147]
MHC locus, pherormone for non same MHC selection	Olfactory receptor 93	2,31	signal transduction/sensory perception of smell	signal transducer activity/receptor activity	membrane	102169_at	AL078630	Olfr93 [148]
Metabolic process	Carbonic anhydrase 3	2,23	one-carbon compound metabolic process	carbonate dehydratase activity	cytoplasm	160375_at	AJ006474	Car3 [149]
Electron transport/ protein folding Obesity	ERO1-like beta (S. cerevisiae)	2,2	electron transport	oxidoreductase activity	endoplasmic reticulum	103531_f_at	AI049144	Ero1lb [150], [151]
Protein binding, vesicle transport	Coatomer protein complex, subunit zeta 2	1,96	protein complex assembly/intracellular protein transport/ER to Golgi vesicle-mediated transport	protein binding	cytoplasm	104143_at	AI843212	Copz2 [152]
Lipoprotein, Receptor activity	Low density lipoprotein receptor-related protein associated protein 1	1,94	---	low-density lipoprotein receptor binding	extracellular	100086_at	D00622	Lrpap1 [153]
Role in Notch/NFkB in neurons	kallikrein 1-related pepidase b4 or alpha-NGF	1,89	proteolysis/small GTPase mediated signal transduction/positive regulation of phosphoinositide 3-kinase activity	serine-type	extracellular	94773_at	X01801	Klk1b4; NGFalpha [41], [10] [42]
Homeostasis	Glutathione transferase zeta 1 (maleylacetoacetate isomerase)	1,87	L-phenylalanine catabolic process	catalytic activity	cytoplasm/mitochondrion	160350_at	AW060750	Gstz1 [154]
Metabolism	Aldehyde dehydrogenase family 1, subfamily A7	1,86	metabolic process	oxidoreductase activity	cytoplasm	94778_at	U96401	Aldh1a7 [115], [155]
Metabolic process	Gysteine dioxygenase 1, cytosolic	1,84	L-cysteine metabolic process	iron ion binding	cytosol	96346_at	AI854020	Cdo1 [156]
Hematopoiesis and Wnt-beta catenin pathway in the brain Ulcerative colitis, cancer	Frizzled homolog 3 (Drosophila)	1,84	signal transduction /multicellular organismal development/Wnt receptor signaling pathway	signal transducer activity/receptor activity	extracellular	98169_s_at	AU020229	Fzd3 [157]
Homeostasis, Gut	Lecithin cholesterol acyltransferase	1,78	lipid metabolic process	catalytic activity	extracellular	103023_at	J05154	Lcat [158]
Cytoskeleton organization and biogenesis nucleotide binding	Actin, alpha 2, smooth muscle, aorta	1,77	cytoskeleton organization and biogenesis	nucleotide binding	cytoplasm	93100_at	X13297	Acta2 [159]
Chromosome	Inactive X specific transcripts	1,6	dosage compensation, by inactivation of X chromosome	---	chromosome	99126_at	L04961	Xist

Apoptosis, translation	Eukaryotic translation elongation factor 1 alpha 1	-1,25	translational elongation	nucleotide binding	cytoplasm	94766_at	M17878	Eef1a1 [160]
DNA synthesis	Proliferating cell nuclear antigen	-1,73	DNA replication/intracellular protein transport	DNA binding	nucleus	101065_at	X57800	Pcna [161]
Development	SLAIN motif family, member 1	-1,76	---	---	---	160236_at	AI835624	Slain1 [162]
Repair of nucleotide-excision establishment and/or maintenance of chromatin architecture	High mobility group nucleosomal binding domain 1	-2	transcription-couplednucleotide-excision repair/establishment &/or maintenance of chromatin architecture	DNA binding/chromatin binding	nucleus	96699_at	X53476	Hmgn1 [163-165]
Cell Differentiation, vesicle transport, expressed in the brain	Nuclear distribution gene E homolog 1 (A nidulans)	-2,12	cell cycle/mitosis/development/cell differentiation and division	microtubule binding/identical protein binding	cytoplasm	94910_at	AW120739	Nde1 [166]
Meiosis regulated novel Xlr-related (Xmr) family member	Xmr protein	-2,14	---	---	---	102818_at	X72697	Slx, Xmr [167]

Unknown Function

Putative Function	Gene title	Fold change	GO biological process term	GO molecular function term	GO cellular component term	Affymetrix ID	Representative Public ID	Gene symbol [Reference]
Unknown	RIKEN cDNA 1810010M01, Similar to Spink3 gene	42,36	---	sugar binding	---	160145_at	AI593999	1810010M01Rik (EST)
Unknown	RIKEN cDNA 2210010C04 gene, Similar to Trypsinogen 7	20,03	proteolysis	serine-type	extracellular	100103_f_at; 100104_r_at	AE000663	2210010C04Rik (EST)
Unknown, gastrointestinal tract	Cholecystokinin A receptor	11,91	signal transduction /G-protein coupled receptor protein signaling pathway	cholecystokinin receptor activity	membrane	92252_at	D85605	Cckar [168]
Unknown	RIKEN cDNA 1810009J06 gene, TCR V beta locus	3,96	proteolysis	serine-type	extracellular	92712_at; 101336_at	AE000663	TCRVb [110]
Cancer Unknown function in the PaLN	Rho GDP dissociation inhibitor (GDI) gamma	3,85	---	Rho GDP-dissociation inhibitor activity/GTPase activator activity	cytoplasm	102677_at	U73198	Arhgdig [169]
Unknown function in the PaLN	Glycine N-methyl transferase	3,62	---	catalytic activity	cytoplasm	96828_at	D89664	Gnmt [169]
Unknown function in the PaLN, Cofactor for HNF1	Pterin 4 alpha carbinolamine dehydratase/dimerization cofactor of hepatocyte nuclear factor 1 alpha (TCF1) 1	3,37	positive regulation of transcription	transcription coactivator activity	nucleus	99056_at	AW046590	Pcbd1 [170]
Unknown	Murine leukemia retrovirus	2,64	DNA integration/virus assembly	nucleic acid binding/ribonuclease H activity	integral to membrane	103562_f_at	M26005	Gv-1 [171], [172]; [173]

Unknown	cDNA sequence BC003494	2,63	---	---	---	161677_r_at	AV171666	EST
Unknown	Guanidino acetate methyltransfer ase	2,52	biosynthetic process/organ morphogenesis/r egulation of body size	methyltransfer ase activity	---	101408_at	AF010499	Gamt, [174]; [175]
Unknown function	Glycine amidinotransfe rase	2,2	transport	receptor activity	mitochondrion	96336_at	AI844626	Gatm, [176]
Unkown, some role in adipocytes	Thyroid hormone responsive SPOT14 homolog (Rattus)	1,88	---	---	---	160306_at	X95279	Thrsp [177]
Unknown	EST	1,87	---	---	---	161131_r_at	AV250694	EST
Unknown	RIKEN cDNA 1810029G24 gene Homologue to Sec11c yeast	1,73	signal peptide processing/prote olysis	serine-type peptidase activity	membrane	160250_at	AW049643	EST
Unknown	cDNA sequence BC008155	1,57	---	---	---	161641_at	AV067171	EST
Unknown	RIKEN cDNA 0610010K14 gene	-0,47	---	DNA binding	nucleus	95634_at	AI848107	0610010K14Rik (EST)
Unknown	RIKEN cDNA 2510049I19 gene	-0,61	---	---	nucleolus	97866_at	AI842858	2510049I19Rik (EST)
Unknown function	RIKEN cDNA C330027C09 gene	-1,44	---	binding	cytoplasm	160973_at	NM_17261 6 AA590345	RTRtv
Unknown function	PCI domain containing 2	-1,65	---	---	---	96545_s_at	AI447510	Pcid2 [178]
Unknown function	Gene trap ROSA 26, Philippe Soriano	-1,68	---	---	---	160620_at	U83174	Gt(ROSA)26S

Ubiquitination

Putative Function	Gene title	Fold change	GO biological process term	GO molecular function term	GO cellular component term	Affymetrix ID	Representa tive Public ID	Gene symbol [Reference]
Ubiquitination	Heterogeneous nuclear ribonucleo-protein C	-1,62	mRNA processing and splicing	nucleotide binding	nucleus	160199_at	AF095257	Hnrpc [177]
Related to ubiquitin	Heterogeneous nuclear ribonucleo-protein R	-1,76	---	nucleotide binding	nucleus	97491_at	AW123032	Hnrpr [179]
Ubiquitination, Degradation enzyme	Ubiquitin-conjugating enzyme E2E 1, UBC4/5 homolog (yeast)	-1,74	ubiquitin cycle	ubiquitin-protein ligase activity	---	92660_f_at	X92665	Ube2e1 [180], [181]
Ubiquitination Oncogenesis immune system	Similar to mouse RING finger 1	-1,77	---	protein binding	intracellular	99149_at	AI851230	Trim59 [182]
Ubiquitination, found in diabeticity	Ubiquitin specific peptidase 1	-1,8	ubiquitin-dependent protein catabolic process	ubiquitin thiolesterase activity	---	160234_at	AI848382	Usp1 [115]
Ubiquitin cycle	Zinc and ring finger 2	-2,69	ubiquitin cycle	protein binding	membrane	99574_at	AI850887	Znrf2 [183]

Transcription

Putative Function	Gene title	Fold change	GO biological process term	GO molecular function term	GO cellular component term	Affymetrix ID	Representa tive Public ID	Gene symbol [Reference]
Transcription, development gut & pancreas	Forkhead box A3	1,76	cell glucose homeostasis/transcription/cellular response to starvation	transcription factor activity	nucleus/transcription factor complex	98324_at	X74938	Foxa3 [184]; [185]
Transcription factor	Trans-acting transcription factor 4	-1,36	regulation of transcription	transcription factor activity	transcription factor complex	92992_i_at	AI324972	Sp4 [186]
Transcription factor in B cells, Ig	Spi-C transcription factor (Spi-1/PU.1 related)	-1,69	transcription	transcription factor activity	nucleus	103454_at	AA182189	Spic [187]

Bibliography

1. Cash HL, Whitham CV, Behrendt CL, Hooper LV. Symbiotic bacteria direct expression of an intestinal bactericidal lectin. Science 2006; **313**:1126-30.
2. Abe M, Nata K, Akiyama T, Shervani NJ, Kobayashi S, Tomioka-Kumagai T, Ito S, Takasawa S, Okamoto H. Identification of a novel Reg family gene, Reg IIIdelta, and mapping of all three types of Reg family gene in a 75 kilobase mouse genomic region. Gene 2000; **246**:111-22.
3. Stevenson BJ, Hagenbuchle O, Wellauer PK. Sequence organisation and transcriptional regulation of the mouse elastase II and trypsin genes. Nucleic Acids Res 1986; **14**:8307-30.
4. Algul H, Treiber M, Lesina M, Nakhai H, Saur D, Geisler F, Pfeifer A, Paxian S, Schmid RM. Pancreas-specific RelA/p65 truncation increases susceptibility of acini to inflammation-associated cell death following cerulein pancreatitis. J Clin Invest 2007; **117**:1490-501.
5. Otonkoski T, Mally MI, Hayek A. Opposite effects of beta-cell differentiation and growth on reg expression in human fetal pancreatic cells. Diabetes 1994; **43**:1164-6.
6. Fox JG, Rogers AB, Whary MT, Ge Z, Ohtani M, Jones EK, Wang TC. Accelerated progression of gastritis to dysplasia in the pyloric antrum of TFF2 -/- C57BL6 x Sv129 Helicobacter pylori-infected mice. Am J Pathol 2007; **171**:1520-8.
7. Ribieras S, Lefebvre O, Tomasetto C, Rio MC. Mouse Trefoil factor genes: genomic organization, sequences and methylation analyses. Gene 2001; **266**:67-75.
8. Kurt-Jones EA, Cao L, Sandor F, Rogers AB, Whary MT, Nambiar PR, Cerny A, Bowen G, Yan J, Takaishi S, Chi AL, Reed G, Houghton J, Fox JG, Wang TC. Trefoil family factor 2 is expressed in murine gastric and immune cells and controls both gastrointestinal inflammation and systemic immune responses. Infect Immun 2007; **75**:471-80.
9. Schelhaas M, Malmstrom J, Pelkmans L, Haugstetter J, Ellgaard L, Grunewald K, Helenius A. Simian Virus 40 depends on ER protein folding and quality control factors for entry into host cells. Cell 2007; **131**:516-29.
10. Lundwall A, Band V, Blaber M, Clements JA, Courty Y, Diamandis EP, Fritz H, Lilja H, Malm J, Maltais LJ, Olsson AY, Petraki C, Scorilas A, Sotiropoulou G, Stenman UH, Stephan C, Talieri M, Yousef GM. A comprehensive nomenclature for serine proteases with homology to tissue kallikreins. Biol Chem 2006; **387**:637-41.
11. Lundwall A, Lilja H. Molecular cloning of human prostate specific antigen cDNA. FEBS Lett 1987; **214**:317-22.
12. Olsson AY, Lundwall A. Organization and evolution of the glandular kallikrein locus in Mus musculus. Biochem Biophys Res Commun 2002; **299**:305-11.
13. Pileggi A, Molano RD, Song S, Zahr E, Sanjose S, Villate S, Wasserfall C, Ricordi C, Atkinson MA, Inverardi L. Alpha-1 antitrypsin treatment of spontaneously diabetic nonobese diabetic mice receiving islet allografts. Transplant Proc 2008; **40**:457-8.
14. Cash HL, Whitham CV, Hooper LV. Refolding, purification, and characterization of human and murine RegIII proteins expressed in Escherichia coli. Protein Expr Purif 2006; **48**:151-9.
15. Christophi GP, Isackson PJ, Blaber S, Blaber M, Rodriguez M, Scarisbrick IA. Distinct promoters regulate tissue-specific and differential expression of kallikrein 6 in CNS demyelinating disease. J Neurochem 2004; **91**:1439-49.
16. Takada K, Takiguchi M, Konno A, Inaba M. Autoimmunity against a tissue kallikrein in IQI/Jic Mice: a model for Sjogren's syndrome. J Biol Chem 2005; **280**:3982-8.
17. Imamura T, Asada M, Vogt SK, Rudnick DA, Lowe ME, Muglia LJ. Protection from pancreatitis by the zymogen granule membrane protein integral membrane-associated protein-1. J Biol Chem 2002; **277**:50725-33.
18. Costanzi S, Neumann S, Gershengorn MC. Seven transmembrane-spanning receptors for free fatty acids as therapeutic targets for diabetes mellitus: pharmacological, phylogenetic and drug discovery aspects. J Biol Chem 2008.
19. Labonte ED, Kirby RJ, Schildmeyer NM, Cannon AM, Huggins KW, Hui DY. Group 1B phospholipase A2-mediated lysophospholipid absorption directly contributes to postprandial hyperglycemia. Diabetes 2006; **55**:935-41.
20. Agassandian M, Miakotina OL, Andrews M, Mathur SN, Mallampalli RK. Pseudomonas aeruginosa and sPLA2 IB stimulate ABCA1-mediated phospholipid efflux via ERK-activation of PPARalpha-RXR. Biochem J 2007; **403**:409-20.
21. Muller M, Trocme C, Lardy B, Morel F, Halimi S, Benhamou PY. Matrix metalloproteinases and diabetic foot ulcers: the ratio of MMP-1 to TIMP-1 is a predictor of wound healing. Diabet Med 2008; **25**:419-26.
22. Vasseur S, Mallo GV, Garcia-Montero A, Ortiz EM, Fiedler F, Canepa E, Moreno S, Iovanna JL. Structural and functional characterization of the mouse p8 gene: promotion of transcription by the CAAT-enhancer binding protein alpha (C/EBPalpha) and C/EBPbeta trans-acting factors involves a C/EBP cis-acting element and other regions of the promoter. Biochem J 1999; **343 Pt 2**:377-83.
23. Vasseur S, Folch-Puy E, Hlouschek V, Garcia S, Fiedler F, Lerch MM, Dagorn JC, Closa D, Iovanna JL. p8 improves pancreatic response to acute pancreatitis by enhancing the expression of the anti-inflammatory protein pancreatitis-associated protein I. J Biol Chem 2004; **279**:7199-207.
24. Vasseur S, Hoffmeister A, Garcia S, Bagnis C, Dagorn JC, Iovanna JL. p8 is critical for tumour development induced by rasV12 mutated protein and E1A oncogene. EMBO Rep 2002; **3**:165-70.
25. Vasseur S, Hoffmeister A, Garcia-Montero A, Mallo GV, Feil R, Kuhbandner S, Dagorn JC, Iovanna JL. p8-deficient fibroblasts grow more rapidly and are more resistant to adriamycin-induced apoptosis. Oncogene 2002; **21**:1685-94.
26. Plant SR, Wang Y, Vasseur S, Thrash JC, McMahon EJ, Bergstralh DT, Arnett HA, Miller SD, Carson MJ, Iovanna JL, Ting JP. Upregulation of the stress-associated gene p8 in mouse models of demyelination and in multiple sclerosis tissues. Glia 2006; **53**:529-37.
27. Greenlee KJ, Corry DB, Engler DA, Matsunami RK, Tessier P, Cook RG, Werb Z, Kheradmand F. Proteomic identification of in vivo substrates for matrix metalloproteinases 2 and 9 reveals a mechanism for resolution of inflammation. J Immunol 2006; **177**:7312-21.
28. Mori R, Shaw TJ, Martin P. Molecular mechanisms linking wound inflammation and fibrosis: knockdown of osteopontin leads to rapid repair and reduced scarring. J Exp Med 2008; **205**:43-51.
29. Kon S, Ikesue M, Kimura C, Aoki M, Nakayama Y, Saito Y, Kurotaki D, Diao H, Matsui Y, Segawa T, Maeda M, Kojima T, Uede T. Syndecan-4 protects against osteopontin-mediated acute hepatic injury by masking functional domains of osteopontin. J Exp Med 2008; **205**:25-33.
30. McDonald B, McAvoy EF, Lam F, Gill V, de la Motte C, Savani RC, Kubes P. Interaction of CD44 and hyaluronan is the dominant mechanism for neutrophil sequestration in inflamed liver sinusoids. J Exp Med 2008; **205**:915-27.
31. Arafat HA, Katakam AK, Chipitsyna G, Gong Q, Vancha AR, Gabbeta J, Dafoe DC. Osteopontin protects the islets and beta-cells from interleukin-1 beta-mediated cytotoxicity through negative feedback regulation of nitric oxide. Endocrinology 2007; **148**:575-84.

32. Bidder M, Shao JS, Charlton-Kachigian N, Loewy AP, Semenkovich CF, Towler DA. Osteopontin transcription in aortic vascular smooth muscle cells is controlled by glucose-regulated upstream stimulatory factor and activator protein-1 activities. J Biol Chem 2002; **277**:44485-96.
33. Mo B, Vendrov AE, Palomino WA, DuPont BR, Apparao KB, Lessey BA. ECC-1 cells: a well-differentiated steroid-responsive endometrial cell line with characteristics of luminal epithelium. Biol Reprod 2006; **75**:387-94.
34. Casanova ML, Bravo A, Ramirez A, Morreale de Escobar G, Were F, Merlino G, Vidal M, Jorcano JL. Exocrine pancreatic disorders in transsgenic mice expressing human keratin 8. J Clin Invest 1999; **103**:1587-95.
35. Demetriou M, Binkert C, Sukhu B, Tenenbaum HC, Dennis JW. Fetuin/alpha2-HS glycoprotein is a transforming growth factor-beta type II receptor mimic and cytokine antagonist. J Biol Chem 1996; **271**:12755-61.
36. Woltje M, Tschoke B, von Bulow V, Westenfeld R, Denecke B, Graber S, Jahnen-Dechent W. CCAAT enhancer binding protein beta and hepatocyte nuclear factor 3beta are necessary and sufficient to mediate dexamethasone-induced up-regulation of alpha2HS-glycoprotein/fetuin-A gene expression. J Mol Endocrinol 2006; **36**:261-77.
37. Tsujimura T, Morii E, Nozaki M, Hashimoto K, Moriyama Y, Takebayashi K, Kondo T, Kanakura Y, Kitamura Y. Involvement of transcription factor encoded by the mi locus in the expression of c-kit receptor tyrosine kinase in cultured mast cells of mice. Blood 1996; **88**:1225-33.
38. Treiber M, Schulz HU, Landt O, Drenth JP, Castellani C, Real FX, Akar N, Ammann RW, Bargetzi M, Bhatia E, Demaine AG, Battagia C, Kingsnorth A, O'Reilly D, Truninger K, Koudova M, Spicak J, Cerny M, Menzel HJ, Moral P, Pignatti PF, Romanelli MG, Rickards O, De Stefano GF, Zarnescu NO, Choudhuri G, Sikora SS, Jansen JB, Weiss FU, Pietschmann M, Teich N, Gress TM, Ockenga J, Schmidt H, Kage A, Halangk J, Rosendahl J, Groneberg DA, Nickel R, Witt H. Keratin 8 sequence variants in patients with pancreatitis and pancreatic cancer. J Mol Med 2006; **84**:1015-22.
39. Tsuruta Y, Park YJ, Siegal GP, Liu G, Abraham E. Involvement of vitronectin in lipopolysaccaride-induced acute lung injury. J Immunol 2007; **179**:7079-86.
40. Kilic G, Wang J, Sosa-Pineda B. Osteopontin is a novel marker of pancreatic ductal tissues and of undifferentiated pancreatic precursors in mice. Dev Dyn 2006; **235**:1659-67.
41. Fukushima D, Kitamura N, Nakanishi S. Nucleotide sequence of cloned cDNA for human pancreatic kallikrein. Biochemistry 1985; **24**:8037-43.
42. Schedlich LJ, Bennetts BH, Morris BJ. Primary structure of a human glandular kallikrein gene. DNA 1987; **6**:429-37.
43. Shanmukhappa K, Mourya R, Sabla GE, Degen JL, Bezerra JA. Hepatic to pancreatic switch defines a role for hemostatic factors in cellular plasticity in mice. Proc Natl Acad Sci U S A 2005; **102**:10182-7.
44. Hiratsuka S, Watanabe A, Aburatani H, Maru Y. Tumour-mediated upregulation of chemoattractants and recruitment of myeloid cells predetermines lung metastasis. Nat Cell Biol 2006; **8**:1369-75.
45. Yu X, Bauer K, Wernhoff P, Koczan D, Moller S, Thiesen HJ, Ibrahim SM. Fine mapping of collagen-induced arthritis quantitative trait loci in an advanced intercross line. J Immunol 2006; **177**:7042-9.
46. Hirsh MI, Hashiguchi N, Chen Y, Yip L, Junger WG. Surface expression of HSP72 by LPS-stimulated neutrophils facilitates gammadeltaT cell-mediated killing. Eur J Immunol 2006; **36**:712-21.
47. Coghill EL, Hugill A, Parkinson N, Davison C, Glenister P, Clements S, Hunter J, Cox RD, Brown SD. A gene-driven approach to the identification of ENU mutants in the mouse. Nat Genet 2002; **30**:255-6.
48. Nakata M, Okada T, Ozawa K, Yada T. Resistin induces insulin resistance in pancreatic islets to impair glucose-induced insulin release. Biochem Biophys Res Commun 2007; **353**:1046-51.
49. Lu SC, Chang SF, Chen HL, Chou YY, Lan YH, Chuang CY, Yu WH, Chen CL. A novel role for Oct-2 in the lipopolysaccharide-mediated induction of resistin gene expression in RAW264.7 cells. Biochem J 2007; **402**:387-95.
50. Steppan CM, Bailey ST, Bhat S, Brown EJ, Banerjee RR, Wright CM, Patel HR, Ahima RS, Lazar MA. The hormone resistin links obesity to diabetes. Nature 2001; **409**:307-12.
51. De Franco M, Carneiro Pdos S, Peters LC, Vorraro F, Borrego A, Ribeiro OG, Starobinas N, Cabrera WK, Ibanez OM. Slc11a1 (Nramp1) alleles interact with acute inflammation loci to modulate wound-healing traits in mice. Mamm Genome 2007; **18**:263-9.
52. Dejkhamron P, Thimmarayappa J, Kotlyarevska K, Sun J, Lu C, Bonkowski EL, Denson LA, Menon RK. Lipopolysaccharide (LPS) directly suppresses growth hormone receptor (GHR) expression through MyD88-dependent and -independent Toll-like receptor-4/MD2 complex signaling pathways. Mol Cell Endocrinol 2007; **274**:35-42.
53. Crow YJ, Leitch A, Hayward BE, Garner A, Parmar R, Griffith E, Ali M, Semple C, Aicardi J, Babul-Hirji R, Baumann C, Baxter P, Bertini E, Chandler KE, Chitayat D, Cau D, Dery C, Fazzi E, Goizet C, King MD, Klepper J, Lacombe D, Lanzi G, Lyall H, Martinez-Frias ML, Mathieu M, McKeown C, Monier A, Oade Y, Quarrell OW, Rittey CD, Rogers RC, Sanchis A, Stephenson JB, Tacke U, Till M, Tolmie JL, Tomlin P, Voit T, Weschke B, Woods CG, Lebon P, Bonthron DT, Ponting CP, Jackson AP. Mutations in genes encoding ribonuclease H2 subunits cause Aicardi-Goutieres syndrome and mimic congenital viral brain infection. Nat Genet 2006; **38**:910-6.
54. Bach JP, Borta H, Ackermann W, Faust F, Borchers O, Schrader M. The secretory granule protein syncollin localizes to HL-60 cells and neutrophils. J Histochem Cytochem 2006; **54**:877-88.
55. Edwardson JM, An S, Jahn R. The secretory granule protein syncollin binds to syntaxin in a Ca2(+)-sensitive manner. Cell 1997; **90**:325-33.
56. Kang W, Reid KB. DMBT1, a regulator of mucosal homeostasis through the linking of mucosal defense and regeneration? FEBS Lett 2003; **540**:21-5.
57. Wiesner O, Litwiller RD, Hummel AM, Viss MA, McDonald CJ, Jenne DE, Fass DN, Specks U. Differences between human proteinase 3 and neutrophil elastase and their murine homologues are relevant for murine model experiments. FEBS Lett 2005; **579**:5305-12.
58. Thongboonkerd V, Barati MT, McLeish KR, Pierce WM, Epstein PN, Klein JB. Proteomics and diabetic nephropathy. Contrib Nephrol 2004; **141**:142-54.
59. Snider GL. Clinical relevance summary: Collagen vs elastin in pathogenesis of emphysema; cellular origin of elastases; bronchiolitis vs emphysema as a cause of airflow obstruction. Chest 2000; **117**:244S-6S.
60. Raptis SZ, Shapiro SD, Simmons PM, Cheng AM, Pham CT. Serine protease cathepsin G regulates adhesion-dependent neutrophil effector functions by modulating integrin clustering. Immunity 2005; **22**:679-91.
61. Mason AJ, Evans BA, Cox DR, Shine J, Richards RI. Structure of mouse kallikrein gene family suggests a role in specific processing of biologically active peptides. Nature 1983; **303**:300-7.
62. Beintema JJ, Kleineidam RG. The ribonuclease A superfamily: general discussion. Cell Mol Life Sci 1998; **54**:825-32.
63. Hill JA, Bell DA, Brintnell W, Yue D, Wehrli B, Jevnikar AM, Lee DM, Hueber W, Robinson WH, Cairns E. Arthritis induced by posttranslationally modified (citrullinated) fibrinogen in DR4-IE transgenic mice. J Exp Med 2008; **205**:967-79.
64. Palumbo JS, Talmage KE, Massari JV, La Jeunesse CM, Flick MJ, Kombrinck KW, Jirouskova M, Degen JL. Platelets and fibrin(ogen) increase metastatic potential by impeding natural killer cell-mediated elimination of tumor cells. Blood 2005; **105**:178-85.
65. Elson GC, Graber P, Losberger C, Herren S, Gretener D, Menoud LN, Wells TN, Kosco-Vilbois MH, Gauchat JF. Cytokine-like factor-1, a novel soluble protein, shares homology with members of the cytokine type I receptor family. J Immunol 1998; **161**:1371-9.
66. Miura-Shimura Y, Nakamura K, Ohtsuji M, Tomita H, Jiang Y, Abe M, Zhang D, Hamano Y, Tsuda H, Hashimoto H, Nishimura H, Taki S, Shirai T, Hirose S. C1q regulatory region polymorphism down-regulating murine c1q protein levels with linkage to lupus nephritis. J Immunol 2002; **169**:1334-9.
67. Dyer KD, Rosenberg HF. The mouse RNase 4 and RNase 5/ang 1 locus utilizes dual promoters for tissue-specific expression. Nucleic Acids Res 2005; **33**:1077-86.
68. Abrera-Abeleda MA, Xu Y, Pickering MC, Smith RJ, Sethi S. Mesangial immune complex glomerulonephritis due to complement factor D deficiency. Kidney Int 2007; **71**:1142-7.
69. Elliott JF, Marlin KL, Couch RM. Effect of bacille Calmette-Guerin vaccination on C-peptide secretion in children newly diagnosed with IDDM. Diabetes Care 1998; **21**:1691-3.
70. Elliott MK, Jarmi T, Ruiz P, Xu Y, Holers VM, Gilkeson GS. Effects of complement factor D deficiency on the renal disease of MRL/lpr mice. Kidney Int 2004; **65**:129-38.
71. Min HY, Spiegelman BM. Adipsin, the adipocyte serine protease: gene structure and control of expression by tumor necrosis factor. Nucleic Acids Res 1986; **14**:8879-92.

72. Michaylira CZ, Ramocki NM, Simmons JG, Tanner CK, McNaughton KK, Woosley JT, Greenhalgh CJ, Lund PK. Haplotype insufficiency for suppressor of cytokine signaling-2 enhances intestinal growth and promotes polyp formation in growth hormone-transgenic mice. Endocrinology 2006; **147**:1632-41.
73. Michaylira CZ, Simmons JG, Ramocki NM, Scull BP, McNaughton KK, Fuller CR, Lund PK. Suppressor of cytokine signaling-2 limits intestinal growth and enterotrophic actions of IGF-I in vivo. Am J Physiol Gastrointest Liver Physiol 2006; **291**:G472-81.
74. Beswick EJ, Bland DA, Suarez G, Barrera CA, Fan X, Reyes VE. Helicobacter pylori binds to CD74 on gastric epithelial cells and stimulates interleukin-8 production. Infect Immun 2005; **73**:2736-43.
75. Andzelm MM, Chen X, Krzewski K, Orange JS, Strominger JL. Myosin IIA is required for cytolytic granule exocytosis in human NK cells. J Exp Med 2007; **204**:2285-91.
76. Yoshizaki T, Imamura T, Babendure JL, Lu JC, Sonoda N, Olefsky JM. Myosin 5a is an insulin-stimulated Akt2 (protein kinase Bbeta) substrate modulating GLUT4 vesicle translocation. Mol Cell Biol 2007; **27**:5172-83.
77. Altmann K, Frank M, Neumann D, Jakobs S, Westermann B. The class V myosin motor protein, Myo2, plays a major role in mitochondrial motility in Saccharomyces cerevisiae. J Cell Biol 2008; **181**:119-30.
78. Mehta AD, Rock RS, Rief M, Spudich JA, Mooseker MS, Cheney RE. Myosin-V is a processive actin-based motor. Nature 1999; **400**:590-3.
79. Morin NA, Oakes PW, Hyun YM, Lee D, Chin YE, King MR, Springer TA, Shimaoka M, Tang JX, Reichner JS, Kim M. Nonmuscle myosin heavy chain IIA mediates integrin LFA-1 de-adhesion during T lymphocyte migration. J Exp Med 2008; **205**:195-205.
80. Kwok WW, Domeier ML, Raymond FC, Byers P, Nepom GT. Allele-specific motifs characterize HLA-DQ interactions with a diabetes-associated peptide derived from glutamic acid decarboxylase. J Immunol 1996; **156**:2171-7.
81. Borg NA, Wun KS, Kjer-Nielsen L, Wilce MC, Pellicci DG, Koh R, Besra GS, Bharadwaj M, Godfrey DI, McCluskey J, Rossjohn J. CD1d-lipid-antigen recognition by the semi-invariant NKT T-cell receptor. Nature 2007; **448**:44-9.
82. Stronge VS, Salio M, Jones EY, Cerundolo V. A closer look at CD1d molecules: new horizons in studying NKT cells. Trends Immunol 2007; **28**:455-62.
83. Kahn J, Mehraban F, Ingle G, Xin X, Bryant JE, Vehar G, Schoenfeld J, Grimaldi CJ, Peale F, Draksharapu A, Lewin DA, Gerritsen ME. Gene expression profiling in an in vitro model of angiogenesis. Am J Pathol 2000; **156**:1887-900.
84. Liu J, Miwa T, Hilliard B, Chen Y, Lambris JD, Wells AD, Song WC. The complement inhibitory protein DAF (CD55) suppresses T cell immunity in vivo. J Exp Med 2005; **201**:567-77.
85. Dang C, Gottschling M, Manning K, O'Currain E, Schneider S, Sterry W, Stockfleth E, Nindl I. Identification of dysregulated genes in cutaneous squamous cell carcinoma. Oncol Rep 2006; **16**:513-9.
86. Aoki CA, Dawson K, Kenny TP, Gershwin ME, Bowlus CL. Gene expression by PBMC in primary sclerosing cholangitis: evidence for dysregulation of immune mediated genes. Clin Dev Immunol 2006; **13**:265-71.
87. Duchniewicz M, Zemojtel T, Kolanczyk M, Grossmann S, Scheele JS, Zwartkruis FJ. Rap1A-deficient T and B cells show impaired integrin-mediated cell adhesion. Mol Cell Biol 2006; **26**:643-53.
88. Criscitiello MF, Flajnik MF. Four primordial immunoglobulin light chain isotypes, including lambda and kappa, identified in the most primitive living jawed vertebrates. Eur J Immunol 2007; **37**:2683-94.
89. Mukherjee P, Tinder TL, Basu GD, Gendler SJ. MUC1 (CD227) interacts with lck tyrosine kinase in Jurkat lymphoma cells and normal T cells. J Leukoc Biol 2005; **77**:90-9.
90. Malmberg EK, Noaksson KA, Phillipson M, Johansson ME, Hinojosa-Kurtzberg M, Holm L, Gendler SJ, Hansson GC. Increased levels of mucins in the cystic fibrosis mouse small intestine, and modulator effects of the Muc1 mucin expression. Am J Physiol Gastrointest Liver Physiol 2006; **291**:G203-10.
91. Masumoto N, Chen J, Sirotnak FM. Regulation of transcription of the murine gamma-glutamyl hydrolase gene. Delineation of core promoter A and the role of LYF-1, E2F and ETS-1 in determining tumor-specific expression. Gene 2002; **291**:169-76.
92. Wilcox CB, Feddes GO, Willet-Brozick JE, Hsu LC, Deloia JA, Baysal BE. Coordinate up-regulation of TMEM97 and cholesterol biosynthesis genes in normal ovarian surface epithelial cells treated with progesterone: implications for pathogenesis of ovarian cancer. BMC Cancer 2007; **7**:223.
93. Fenske TS, McMahon C, Edwin D, Jarvis JC, Cheverud JM, Minn M, Mathews V, Bogue MA, Province MA, McLeod HL, Graubert TA. Identification of candidate alkylator-induced cancer susceptibility genes by whole genome scanning in mice. Cancer Res 2006; **66**:5029-38.
94. Mueller B, Lilley BN, Ploegh HL. SEL1L, the homologue of yeast Hrd3p, is involved in protein dislocation from the mammalian ER. J Cell Biol 2006; **175**:261-70.
95. Ye Y, Shibata Y, Yun C, Ron D, Rapoport TA. A membrane protein complex mediates retro-translocation from the ER lumen into the cytosol. Nature 2004; **429**:841-7.
96. Moran P, Li W, Fan B, Vij R, Eigenbrot C, Kirchhofer D. Pro-urokinase-type plasminogen activator is a substrate for hepsin. J Biol Chem 2006; **281**:30439-46.
97. Klezovitch O, Chevillet J, Mirosevich J, Roberts RL, Matusik RJ, Vasioukhin V. Hepsin promotes prostate cancer progression and metastasis. Cancer Cell 2004; **6**:185-95.
98. Dundr M, Ospina JK, Sung MH, John S, Upender M, Ried T, Hager GL, Matera AG. Actin-dependent intranuclear repositioning of an active gene locus in vivo. J Cell Biol 2007; **179**:1095-103.
99. Uchikado Y, Inoue H, Haraguchi N, Mimori K, Natsugoe S, Okumura H, Aikou T, Mori M. Gene expression profiling of lymph node metastasis by oligomicroarray analysis using laser microdissection in esophageal squamous cell carcinoma. Int J Oncol 2006; **29**:1337-47.
100. de Zwart-Storm EA, Hamm H, Stoevesandt J, Steijlen PM, Martin PE, van Geel M, van Steensel MA. A novel missense mutation in GJB2 disturbs gap junction protein transport and causes focal palmoplantar keratoderma with deafness. J Med Genet 2008; **45**:161-6.
101. Cui H, Darmanin S, Natsuisaka M, Kondo T, Asaka M, Shindoh M, Higashino F, Hamuro J, Okada F, Kobayashi M, Nakagawa K, Koide H. Enhanced expression of asparagine synthetase under glucose-deprived conditions protects pancreatic cancer cells from apoptosis induced by glucose deprivation and cisplatin. Cancer Res 2007; **67**:3345-55.
102. Zhou X, Stuart A, Dettin LE, Rodriguez G, Hoel B, Gallicano GI. Desmoplakin is required for microvascular tube formation in culture. J Cell Sci 2004; **117**:3129-40.
103. Mink M, Fogelgren B, Olszewski K, Maroy P, Csiszar K. A novel human gene (SARM) at chromosome 17q11 encodes a protein with a SAM motif and structural similarity to Armadillo/beta-catenin that is conserved in mouse, Drosophila, and Caenorhabditis elegans. Genomics 2001; **74**:234-44.
104. Schmidt RL, Park CH, Ahmed AU, Gundelach JH, Reed NR, Cheng S, Knudsen BE, Tang AH. Inhibition of RAS-mediated transformation and tumorigenesis by targeting the downstream E3 ubiquitin ligase seven in absentia homologue. Cancer Res 2007; **67**:11798-810.
105. Choi SC, Kim KD, Kim JT, Kim JW, Lee HG, Kim JM, Jang YS, Yoon DY, Kim KI, Yang Y, Cho DH, Lim JS. Expression of human NDRG2 by myeloid dendritic cells inhibits down-regulation of activated leukocyte cell adhesion molecule (ALCAM) and contributes to maintenance of T cell stimulatory activity. J Leukoc Biol 2008; **83**:89-98.
106. Burchfield JG, Lennard AJ, Narasimhan S, Hughes WE, Wasinger VC, Corthals GL, Okuda T, Kondoh H, Biden TJ, Schmitz-Peiffer C. Akt mediates insulin-stimulated phosphorylation of Ndrg2: evidence for cross-talk with protein kinase C theta. J Biol Chem 2004; **279**:18623-32.
107. Chu CC, Paul WE. Expressed genes in interleukin-4 treated B cells identified by cDNA representational difference analysis. Mol Immunol 1998; **35**:487-502.
108. Azarova AM, Lyu YL, Lin CP, Tsai YC, Lau JY, Wang JC, Liu LF. Roles of DNA topoisomerase II isozymes in chemotherapy and secondary malignancies. Proc Natl Acad Sci U S A 2007; **104**:11014-9.
109. Bucan M, Yang-Feng T, Colberg-Poley AM, Wolgemuth DJ, Guenet JL, Francke U, Lehrach H. Genetic and cytogenetic localisation of the homeo box containing genes on mouse chromosome 6 and human chromosome 7. EMBO J 1986; **5**:2899-905.
110. Chen JM, Piepoli Bis A, Le Bodic L, Ruszniewski P, Robaszkiewicz M, Deprez PH, Raguenes O, Quere I, Andriulli A, Ferec C. Mutational screening of the cationic trypsinogen gene in a large cohort of subjects with idiopathic chronic pancreatitis. Clin Genet 2001; **59**:189-93.
111. Witt H, Sahin-Toth M, Landt O, Chen JM, Kahne T, Drenth JP, Kukor Z, Szepessy E, Halangk W, Dahm S, Rohde K, Schulz HU, Le Marechal C, Akar N, Ammann RW, Truninger K, Bargetzi M, Bhatia E, Castellani C, Cavestro GM, Cerny M, Destro-Bisol G, Spedini G, Eiberg H, Jansen JB, Koudova M, Rausova E, Macek M, Jr., Malats N, Real FX, Menzel HJ, Moral P, Galavotti R, Pignatti PF, Rickards O, Spicak J, Zarnescu NO, Bock W, Gress TM, Friess H, Ockenga J, Schmidt H, Pfutzer R, Lohr M, Simon P, Weiss FU, Lerch MM, Teich N, Keim V, Berg T, Wiedenmann B,

Luck W, Groneberg DA, Becker M, Keil T, Kage A, Bernardova J, Braun M, Guldner C, Halangk J, Rosendahl J, Witt U, Treiber M, Nickel R, Ferec C. A degradation-sensitive anionic trypsinogen (PRSS2) variant protects against chronic pancreatitis. Nat Genet 2006; **38**:668-73.
112. Tomomura A, Fukushige T, Noda T, Noikura T, Saheki T. Serum calcium-decreasing factor (caldecrin) from porcine pancreas has proteolytic activity which has no clear connection with the calcium decrease. FEBS Lett 1992; **301**:277-81.
113. Scriver CR. The PAH gene, phenylketonuria, and a paradigm shift. Hum Mutat 2007; **28**:831-45.
114. Rowen L, Koop BF, Hood L. The complete 685-kilobase DNA sequence of the human beta T cell receptor locus. Science 1996; **272**:1755-62.
115. Brown AC, Olver WI, Donnelly CJ, May ME, Naggert JK, Shaffer DJ, Roopenian DC. Searching QTL by gene expression: analysis of diabesity. BMC Genet 2005; **6**:12.
116. Aida K, Tawata M, Negishi M, Onaya T. Mouse glycine N-methyltransferase is sexually dimorphic and regulated by growth hormone. Horm Metab Res 1997; **29**:646-9.
117. Choi MS, Jung UJ, Yeo J, Kim MJ, Lee MK. Genistein and daidzein prevent diabetes onset by elevating insulin level and altering hepatic gluconeogenic and lipogenic enzyme activities in non-obese diabetic (NOD) mice. Diabetes Metab Res Rev 2008; **24**:74-81.
118. Hofer T, Wenger RH, Kramer MF, Ferreira GC, Gassmann M. Hypoxic up-regulation of erythroid 5-aminolevulinate synthase. Blood 2003; **101**:348-50.
119. Nandan SD, Beale EG. Regulation of phosphoenolpyruvate carboxykinase mRNA in mouse liver, kidney, and fat tissues by fasting, diabetes, and insulin. Lab Anim Sci 1992; **42**:473-7.
120. Kim SJ, Nian C, McIntosh CH. Activation of lipoprotein lipase by glucose-dependent insulinotropic polypeptide in adipocytes. A role for a protein kinase B, LKB1, and AMP-activated protein kinase cascade. J Biol Chem 2007; **282**:8557-67.
121. Kim SJ, Nian C, McIntosh CH. Resistin is a key mediator of glucose-dependent insulinotropic polypeptide (GIP) stimulation of lipoprotein lipase (LPL) activity in adipocytes. J Biol Chem 2007; **282**:34139-47.
122. Brownstein DG, Bhatt PN, Gras L, Jacoby RO. Chromosomal locations and gonadal dependence of genes that mediate resistance to ectromelia (mousepox) virus-induced mortality. J Virol 1991; **65**:1946-51.
123. Soni KG, Lehner R, Metalnikov P, O'Donnell P, Semache M, Gao W, Ashman K, Pshezhetsky AV, Mitchell GA. Carboxylesterase 3 (EC 3.1.1.1) is a major adipocyte lipase. J Biol Chem 2004; **279**:40683-9.
124. Mootha VK, Bunkenborg J, Olsen JV, Hjerrild M, Wisniewski JR, Stahl E, Bolouri MS, Ray HN, Sihag S, Kamal M, Patterson N, Lander ES, Mann M. Integrated analysis of protein composition, tissue diversity, and gene regulation in mouse mitochondria. Cell 2003; **115**:629-40.
125. de Jong H, Neal AC, Coleman RA, Lewin TM. Ontogeny of mRNA expression and activity of long-chain acyl-CoA synthetase (ACSL) isoforms in Mus musculus heart. Biochim Biophys Acta 2007; **1771**:75-82.
126. Grusby MJ, Nabavi N, Wong H, Dick RF, Bluestone JA, Schotz MC, Glimcher LH. Cloning of an interleukin-4 inducible gene from cytotoxic T lymphocytes and its identification as a lipase. Cell 1990; **60**:451-9.
127. Keller SA, Rosenberg MP, Johnson TM, Howard G, Meisler MH. Regulation of amylase gene expression in diabetic mice is mediated by a cis-acting upstream element close to the pancreas-specific enhancer. Genes Dev 1990; **4**:1316-21.
128. Koyama I, Komine S, Iino N, Hokari S, Igarashi S, Alpers DH, Komoda T. alpha-Amylase expressed in human liver is encoded by the AMY-2B gene identified in tumorous tissues. Clin Chim Acta 2001; **309**:73-83.
129. Lidmer AS, Kannius M, Lundberg L, Bjursell G, Nilsson J. Molecular cloning and characterization of the mouse carboxyl ester lipase gene and evidence for expression in the lactating mammary gland. Genomics 1995; **29**:115-22.
130. Raeder H, Johansson S, Holm PI, Haldorsen IS, Mas E, Sbarra V, Nermoen I, Eide SA, Grevle L, Bjorkhaug L, Sagen JV, Aksnes L, Sovik O, Lombardo D, Molven A, Njolstad PR. Mutations in the CEL VNTR cause a syndrome of diabetes and pancreatic exocrine dysfunction. Nat Genet 2006; **38**:54-62.
131. Zhang J, Stanley RA, Melton LD. Lipid peroxidation inhibition capacity assay for antioxidants based on liposomal membranes. Mol Nutr Food Res 2006; **50**:714-24.
132. Lindner I, Helwig U, Rubin D, Li Y, Fisher E, Boeing H, Mohlig M, Spranger J, Pfeiffer A, Hampe J, Schreiber S, Doring F, Schrezenmeir J. Putative association between a new polymorphism in exon 3 (Arg109Cys) of the pancreatic colipase gene and type 2 diabetes mellitus in two independent Caucasian study populations. Mol Nutr Food Res 2005; **49**:972-6.
133. Ae Park S, Choi MS, Cho SY, Seo JS, Jung UJ, Kim MJ, Sung MK, Park YB, Lee MK. Genistein and daidzein modulate hepatic glucose and lipid regulating enzyme activities in C57BL/KsJ-db/db mice. Life Sci 2006; **79**:1207-13.
134. Lowe ME, Kaplan MH, Jackson-Grusby L, D'Agostino D, Grusby MJ. Decreased neonatal dietary fat absorption and T cell cytotoxicity in pancreatic lipase-related protein 2-deficient mice. J Biol Chem 1998; **273**:31215-21.
135. Lu H, Yang Y, Allister EM, Wijesekara N, Wheeler MB. The identification of potential factors associated with the development of type 2 diabetes: A quantitative proteomic approach. Mol Cell Proteomics 2008.
136. Dybkaer K, Iqbal J, Zhou G, Geng H, Xiao L, Schmitz A, d'Amore F, Chan WC. Genome wide transcriptional analysis of resting and IL2 activated human natural killer cells: gene expression signatures indicative of novel molecular signaling pathways. BMC Genomics 2007; **8**:230.
137. Rulten SL, Kinloch RA, Tateossian H, Robinson C, Gettins L, Kay JE. The human FK506-binding proteins: characterization of human FKBP19. Mamm Genome 2006; **17**:322-31.
138. Yang Q, Graham TE, Mody N, Preitner F, Peroni OD, Zabolotny JM, Kotani K, Quadro L, Kahn BB. Serum retinol binding protein 4 contributes to insulin resistance in obesity and type 2 diabetes. Nature 2005; **436**:356-62.
139. Bhanumathy CD, Tang Y, Monga SP, Katuri V, Cox JA, Mishra B, Mishra L. Itih-4, a serine protease inhibitor regulated in interleukin-6-dependent liver formation: role in liver development and regeneration. Dev Dyn 2002; **223**:59-69.
140. Hofstetter AO, Saha S, Siljehav V, Jakobsson PJ, Herlenius E. The induced prostaglandin E2 pathway is a key regulator of the respiratory response to infection and hypoxia in neonates. Proc Natl Acad Sci U S A 2007; **104**:9894-9.
141. Kamoshita E, Ikeda Y, Fujita M, Amano H, Oikawa A, Suzuki T, Ogawa Y, Yamashina S, Azuma S, Narumiya S, Unno N, Majima M. Recruitment of a prostaglandin E receptor subtype, EP3-expressing bone marrow cells is crucial in wound-induced angiogenesis. Am J Pathol 2006; **169**:1458-72.
142. Zhang T, Xiong H, Kan LX, Zhang CK, Jiao XF, Fu G, Zhang QH, Lu L, Tong JH, Gu BW, Yu M, Liu JX, Licht J, Waxman S, Zelent A, Chen E, Chen SJ. Genomic sequence, structural organization, molecular evolution, and aberrant rearrangement of promyelocytic leukemia zinc finger gene. Proc Natl Acad Sci U S A 1999; **96**:11422-7.
143. Keren-Paz A, Bercovich Z, Porat Z, Erez O, Brener O, Kahana C. Overexpression of antizyme-inhibitor in NIH3T3 fibroblasts provides growth advantage through neutralization of antizyme functions. Oncogene 2006; **25**:5163-72.
144. Ivakine EA, Fox CJ, Paterson AD, Mortin-Toth SM, Canty A, Walton DS, Aleksa K, Ito S, Danska JS. Sex-specific effect of insulin-dependent diabetes 4 on regulation of diabetes pathogenesis in the nonobese diabetic mouse. J Immunol 2005; **174**:7129-40.
145. Szathmary EJ. The effect of Gc genotype on fasting insulin level in Dogrib Indians. Hum Genet 1987; **75**:368-72.
146. Yamamoto N, Homma S, Millman I. Identification of the serum factor required for in vitro activation of macrophages. Role of vitamin D3-binding protein (group specific component, Gc) in lysophospholipid activation of mouse peritoneal macrophages. J Immunol 1991; **147**:273-80.
147. Lin P, Li F, Zhang YW, Huang H, Tong G, Farquhar MG, Xu H. Calnuc binds to Alzheimer's beta-amyloid precursor protein and affects its biogenesis. J Neurochem 2007; **100**:1505-14.
148. Amadou C, Younger RM, Sims S, Matthews LH, Rogers J, Kumanovics A, Ziegler A, Beck S, Lindahl KF. Co-duplication of olfactory receptor and MHC class I genes in the mouse major histocompatibility complex. Hum Mol Genet 2003; **12**:3025-40.
149. Pan PW, Rodriguez A, Parkkila S. A systematic quantification of carbonic anhydrase transcripts in the mouse digestive system. BMC Mol Biol 2007; **8**:22.
150. Dias-Gunasekara S, Gubbens J, van Lith M, Dunne C, Williams JA, Kataky R, Scoones D, Lapthorn A, Bulleid NJ, Benham AM. Tissue-specific expression and dimerization of the endoplasmic reticulum oxidoreductase Ero1beta. J Biol Chem 2005; **280**:33066-75.
151. Pagani M, Fabbri M, Benedetti C, Fassio A, Pilati S, Bulleid NJ, Cabibbo A, Sitia R. Endoplasmic reticulum oxidoreductin 1-lbeta (ERO1-Lbeta), a human gene induced in the course of the unfolded protein response. J Biol Chem 2000; **275**:23685-92.
152. Wegmann D, Hess P, Baier C, Wieland FT, Reinhard C. Novel isotypic gamma/zeta subunits reveal three coatomer complexes in mammals. Mol Cell Biol 2004; **24**:1070-80.

153. Deane R, Sagare A, Hamm K, Parisi M, LaRue B, Guo H, Wu Z, Holtzman DM, Zlokovic BV. IgG-assisted age-dependent clearance of Alzheimer's amyloid beta peptide by the blood-brain barrier neonatal Fc receptor. J Neurosci 2005; **25**:11495-503.
154. Blackburn AC, Matthaei KI, Lim C, Taylor MC, Cappello JY, Hayes JD, Anders MW, Board PG. Deficiency of glutathione transferase zeta causes oxidative stress and activation of antioxidant response pathways. Mol Pharmacol 2006; **69**:650-7.
155. Schwab KR, Patterson LT, Hartman HA, Song N, Lang RA, Lin X, Potter SS. Pygo1 and Pygo2 roles in Wnt signaling in mammalian kidney development. BMC Biol 2007; **5**:15.
156. McCoy JG, Bailey LJ, Bitto E, Bingman CA, Aceti DJ, Fox BG, Phillips GN, Jr. Structure and mechanism of mouse cysteine dioxygenase. Proc Natl Acad Sci U S A 2006; **103**:3084-9.
157. You J, Nguyen AV, Albers CG, Lin F, Holcombe RF. Wnt pathway-related gene expression in inflammatory bowel disease. Dig Dis Sci 2008; **53**:1013-9.
158. Li L, Naples M, Song H, Yuan R, Ye F, Shafi S, Adeli K, Ng DS. LCAT-null mice develop improved hepatic insulin sensitivity through altered regulation of transcription factors and suppressors of cytokine signaling. Am J Physiol Endocrinol Metab 2007; **293**:E587-94.
159. Sauter A, Machura K, Neubauer B, Kurtz A, Wagner C. Development of renin expression in the mouse kidney. Kidney Int 2008; **73**:43-51.
160. Chang R, Wang E. Mouse translation elongation factor eEF1A-2 interacts with Prdx-I to protect cells against apoptotic death induced by oxidative stress. J Cell Biochem 2007; **100**:267-78.
161. Woo M, Hakem R, Furlonger C, Hakem A, Duncan GS, Sasaki T, Bouchard D, Lu L, Wu GE, Paige CJ, Mak TW. Caspase-3 regulates cell cycle in B cells: a consequence of substrate specificity. Nat Immunol 2003; **4**:1016-22.
162. Hirst CE, Ng ES, Azzola L, Voss AK, Thomas T, Stanley EG, Elefanty AG. Transcriptional profiling of mouse and human ES cells identifies SLAIN1, a novel stem cell gene. Dev Biol 2006; **293**:90-103.
163. Belova GI, Postnikov YV, Furusawa T, Birger Y, Bustin M. Chromosomal protein HMGN1 enhances the heat shock-induced remodeling of Hsp70 chromatin. J Biol Chem 2008; **283**:8080-8.
164. Birger Y, Catez F, Furusawa T, Lim JH, Prymakowska-Bosak M, West KL, Postnikov YV, Haines DC, Bustin M. Increased tumorigenicity and sensitivity to ionizing radiation upon loss of chromosomal protein HMGN1. Cancer Res 2005; **65**:6711-8.
165. Cherukuri S, Hock R, Ueda T, Catez F, Rochman M, Bustin M. Cell Cycle-dependent Binding of HMGN Proteins to Chromatin. Mol Biol Cell 2008; **19**:1816-24.
166. Feng Y, Walsh CA. Mitotic spindle regulation by Nde1 controls cerebral cortical size. Neuron 2004; **44**:279-93.
167. Stoffel A, Chaurushiya M, Singh B, Levine AJ. Activation of NF-kappaB and inhibition of p53-mediated apoptosis by API2/mucosa-associated lymphoid tissue 1 fusions promote oncogenesis. Proc Natl Acad Sci U S A 2004; **101**:9079-84.
168. Ibarz G, Oiry C, Carnazzi E, Crespy P, Escrieut C, Fourmy D, Galleyrand JC, Gagne D, Martinez J. Cholecystokinin 1 receptor modulates the MEKK1-induced c-Jun trans-activation: structural requirements of the receptor. Br J Pharmacol 2006; **147**:951-8.
169. Adra CN, Iyengar AR, Syed FA, Kanaan IN, Rilo HL, Yu W, Kheraj R, Lin SR, Horiuchi T, Khan S, Weremowicz S, Lim B, Morton CC, Higgs DR. Human ARHGDIG, a GDP-dissociation inhibitor for Rho proteins: genomic structure, sequence, expression analysis, and mapping to chromosome 16p13.3. Genomics 1998; **53**:104-9.
170. Koster S, Thony B, Macheroux P, Curtius HC, Heizmann CW, Pfleiderer W, Ghisla S. Human pterin-4 alpha-carbinolamine dehydratase/dimerization cofactor of hepatocyte nuclear factor-1 alpha. Characterization and kinetic analysis of wild-type and mutant enzymes. Eur J Biochem 1995; **231**:414-23.
171. Levy DE, Lerner RA, Wilson MC. The Gv-1 locus coordinately regulates the expression of multiple endogenous murine retroviruses. Cell 1985; **41**:289-99.
172. Stockert E, Boyse EA, Sato H, Itakura K. Heredity of the GIX thymocyte antigen associated with murine leukemia virus: segregation data simulating genetic linkage. Proc Natl Acad Sci U S A 1976; **73**:2077-81.
173. Obata Y, Stockert E, Boyse EA, Tung JS, Litman GW. Spontaneous autoimmunization to GIX cell surface antigen in hybrid mice. J Exp Med 1976; **144**:533-42.
174. Vitarius JA, Sehayek E, Breslow JL. Identification of quantitative trait loci affecting body composition in a mouse intercross. Proc Natl Acad Sci U S A 2006; **103**:19860-5.
175. Schmidt A, Marescau B, Boehm EA, Renema WK, Peco R, Das A, Steinfeld R, Chan S, Wallis J, Davidoff M, Ullrich K, Waldschutz R, Heerschap A, De Deyn PP, Neubauer S, Isbrandt D. Severely altered guanidino compound levels, disturbed body weight homeostasis and impaired fertility in a mouse model of guanidinoacetate N-methyltransferase (GAMT) deficiency. Hum Mol Genet 2004; **13**:905-21.
176. Sandell LL, Guan XJ, Ingram R, Tilghman SM. Gatm, a creatine synthesis enzyme, is imprinted in mouse placenta. Proc Natl Acad Sci U S A 2003; **100**:4622-7.
177. Grillasca JP, Gastaldi M, Khiri H, Dace A, Peyrol N, Reynier P, Torresani J, Planells R. Cloning and initial characterization of human and mouse Spot 14 genes. FEBS Lett 1997; **401**:38-42.
178. Whitfield ML, Sherlock G, Saldanha AJ, Murray JI, Ball CA, Alexander KE, Matese JC, Perou CM, Hurt MM, Brown PO, Botstein D. Identification of genes periodically expressed in the human cell cycle and their expression in tumors. Mol Biol Cell 2002; **13**:1977-2000.
179. Mizutani A, Fukuda M, Ibata K, Shiraishi Y, Mikoshiba K. SYNCRIP, a cytoplasmic counterpart of heterogeneous nuclear ribonucleoprotein R, interacts with ubiquitous synaptotagmin isoforms. J Biol Chem 2000; **275**:9823-31.
180. Matuschewski K, Hauser HP, Treier M, Jentsch S. Identification of a novel family of ubiquitin-conjugating enzymes with distinct amino-terminal extensions. J Biol Chem 1996; **271**:2789-94.
181. Wang J, Wang CE, Orr A, Tydlacka S, Li SH, Li XJ. Impaired ubiquitin-proteasome system activity in the synapses of Huntington's disease mice. J Cell Biol 2008; **180**:1177-89.
182. Chang R, Xu X, Li MD. Molecular cloning, mapping and characterization of a novel mouse RING finger gene, Mrf1. Gene 2002; **291**:241-9.
183. Araki T, Milbrandt J. ZNRF proteins constitute a family of presynaptic E3 ubiquitin ligases. J Neurosci 2003; **23**:9385-94.
184. Kaestner KH, Lee KH, Schlondorff J, Hiemisch H, Monaghan AP, Schutz G. Six members of the mouse forkhead gene family are developmentally regulated. Proc Natl Acad Sci U S A 1993; **90**:7628-31.
185. Clevidence DE, Overdier DG, Tao W, Qian X, Pani L, Lai E, Costa RH. Identification of nine tissue-specific transcription factors of the hepatocyte nuclear factor 3/forkhead DNA-binding-domain family. Proc Natl Acad Sci U S A 1993; **90**:3948-52.
186. Gollner H, Bouwman P, Mangold M, Karis A, Braun H, Rohner I, Del Rey A, Besedovsky HO, Meinhardt A, van den Broek M, Cutforth T, Grosveld F, Philipsen S, Suske G. Complex phenotype of mice homozygous for a null mutation in the Sp4 transcription factor gene. Genes Cells 2001; **6**:689-97.
187. Schweitzer BL, Huang KJ, Kamath MB, Emelyanov AV, Birshtein BK, DeKoter RP. Spi-C has opposing effects to PU.1 on gene expression in progenitor B cells. J Immunol 2006; **177**:2195-207.
